# Supplementary material for: Tumor evolution and immune microenvironment dynamics in primary and relapsed mantle cell lymphoma
Source: Cell Rep Med. 2025 Aug 27;6(9):102318. doi: 10.1016/j.xcrm.2025.102318 (PMC12490240; doi:10.1016/j.xcrm.2025.102318)
Supplement: Document S1. Figures S1–S9 and Tables S1 and S2 [file mmc1.pdf]

Cell Reports Medicine, Volume *6*

Supplemental information

Tumor evolution and immune microenvironment dynamics in primary and relapsed mantle cell lymphoma

Hui Wan, Weicheng Ren, Mingyu Yang, Man Nie, Agata M. Wasik, Likun Du, Leire de Campos-Mata, Rui Sun, Zhiliang Bai, Archibald Enninful, Yating Wang, Mattias Berglund, Rose-Marie Amini, Xiaobo Li, Chunli Yang, Xiaofei Ye, Zhi-Zhang Yang, Stephen M. Ansell, Dongbing Liu, Mirjam van der Burg, Rong Fan, Kui Wu, Birgitta Sander, and Qiang Pan-Hammarström

SUPPLEMENTARY FIGURES

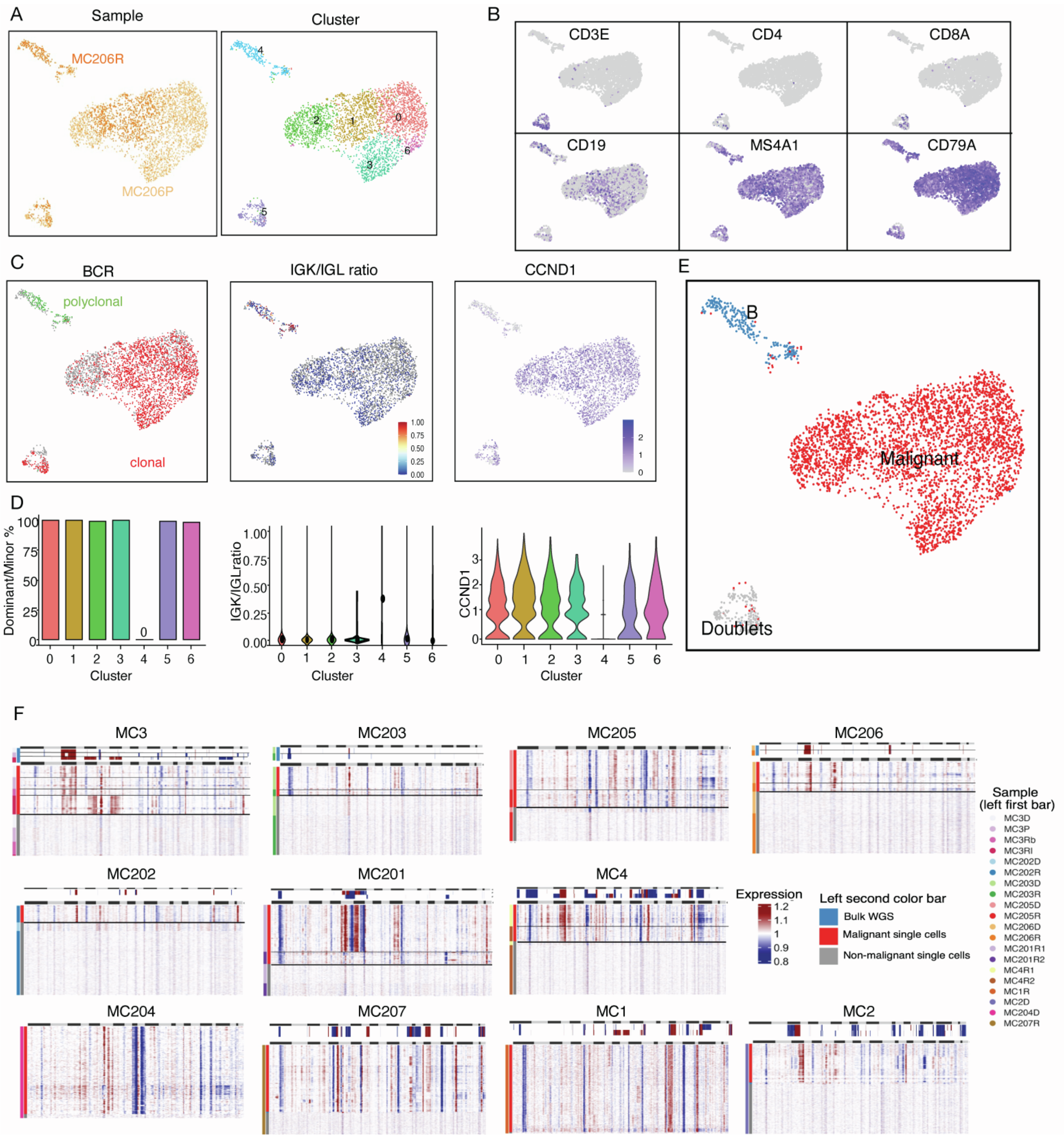

Figure S1. Inferring malignant cells. Related to Figure 1.
Inferring malignant cells using the tumors derived from patient MC206 as an example (A-E). **(A)** UMAP of B cells. Each cell is colored according to the sample origin and clustering number. **(B)** Identification of doublets by checking the gene expression of cell lineage markers. **(C)** UMAP of B cells. Each cell was colored based on the IGK/IGL ratio and clonal BCR. **(D)** Proportion of clonal BCRs and the IGK/IGL ratio for each cell cluster. Each violin plot shows the distribution of IGK/IGL ratio (width represents density), with a central dot indicating the median. **(E)** UMAP of inferred malignant cells and normal B cells. **(F)** Chromosomal landscape CNVs for all MCL samples. The inferred single-cell CNVs are shown with single cells (rows) and chromosomal regions (columns) for each patient. The color in the heatmap represents CNV gain (red) or loss (blue). The cells were grouped into malignant cells and nonmalignant cells, followed by sample origins, which are color-coded on the left. The WGS CNV for matched samples is shown at the top.

We first reclustered all B cells from the two samples derived from this patient and identified seven subclusters (C0-6) (panel A). One subcluster (C5) expressed canonical markers of both B cells and other cell types and was defined as a doublet cluster; therefore, it was excluded from the following analysis (panel B). On the basis of the known features of MCL tumor cells, we annotated five subclusters as malignant cells (C0-3, C6) and one subcluster as nonmalignant cells (C4) (panels C-E). As expected, copy number variants (CNVs), inferred from scRNA-seq data, displayed distinct patterns in malignant cells compared with nonmalignant cells (panel F). Importantly, these CNVs derived from the scRNA-seq data were highly consistent with the CNVs detected from the matched bulk WGS data, confirming the effectiveness of our strategy in identifying malignant B cells.

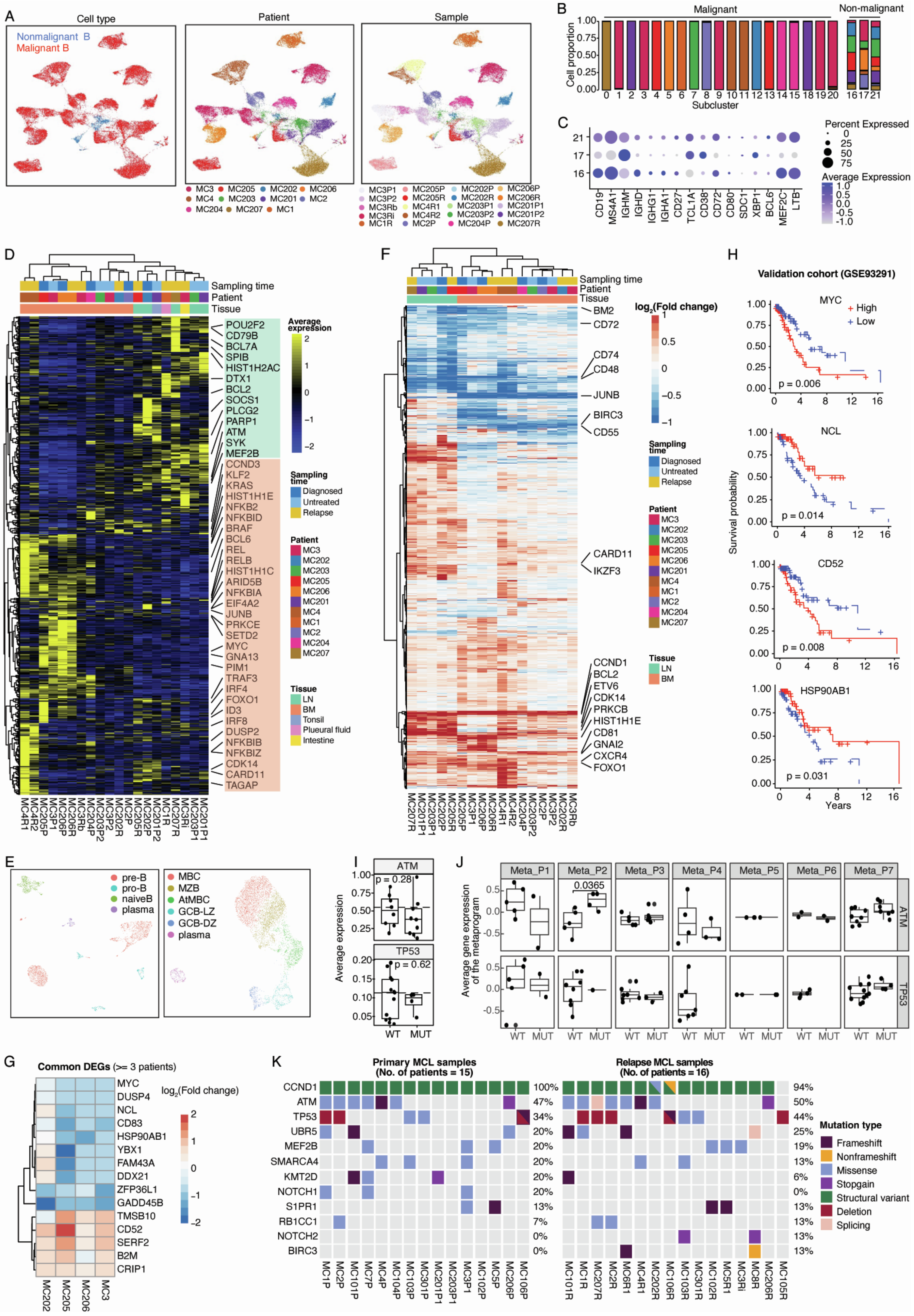
A
Cell type
Nonmalignant B
Malignant B
Patient
MC3 MC205 MC202 MC206
MC4 MC203 MC201 MC2
MC204 MC207 MC1
Sample
MC3P1 MC205P MC202P MC206P
MC3P2 MC205R MC202R MC206R
MC3Rb MC4R1 MC203P1 MC201P1
MC3Ri MC4R2 MC203P2 MC201P2
MC1R MC2P MC204P MC207R
B
Malignant
Non-malignant
Cell proportion
Subcluster
C
Percent Expressed
Average Expression
CD19 MS4A1 IGHM IGHD IGHG1 IGHA1 CD27 TCL1A CD38 CD72 CD80 SDC1 XBP1 BCL6 MEF2C LTB
D
Sampling time
Patient
Tissue
Average expression
POU2F2 CD79B BCL7A SPIB HIST1H2AC DTX1 BCL2 SOCS1 PLCG2 PARP1 ATM SYK MEF2B CCND3 KLF2 KRAS HIST1H1E NFKB2 NFKBID BRAF BCL6 REL RELB HIST1H1C ARID5B NFKBIA EIF4A2 JUNB PRKCE SETD2 MYC GNA13 PIM1 TRAF3 IRF4 FOXO1 ID3 IRF8 DUSP2 NFKBIB NFKBIZ CDK14 CARD11 TAGAP
Diagnosed
Untreated
Relapse
LN
BM
Tonsil
Plueural fluid
Intestine
F
log2(Fold change)
BM2 CD72 CD74 CD48 JUNB BIRC3 CD55 CARD11 IKZF3 CCND1 BCL2 ETV6 CDK14 PRKCB HIST1H1E CD81 GNAI2 CXCR4 FOXO1
H
Validation cohort (GSE93291)
MYC
High
Low
p = 0.006
NCL
p = 0.014
CD52
p = 0.008
HSP90AB1
p = 0.031
Survival probability
Years
E
pre-B
pro-B
naiveB
plasma
MBC
MZB
AtMBC
GCB-LZ
GCB-DZ
plasma
I
ATM
p = 0.28
TP53
p = 0.62
Average expression
WT
MUT
J
Meta_P1 Meta_P2 Meta_P3 Meta_P4 Meta_P5 Meta_P6 Meta_P7
0.0365
Average gene expression of the metaprogram
ATM
TP53
WT MUT
G
Common DEGs (>= 3 patients)
log2(Fold change)
MYC DUSP4 NCL CD83 HSP90AB1 YBX1 FAM43A DDX21 ZFP36L1 GADD45B TMSB10 CD52 SERF2 B2M CRIP1
MC202 MC205 MC206 MC3
K
Primary MCL samples
(No. of patients = 15)
Relapse MCL samples
(No. of patients = 16)
CCND1 ATM TP53 UBR5 MEF2B SMARCA4 KMT2D NOTCH1 S1PR1 RB1CC1 NOTCH2 BIRC3
Mutation type
Frameshift
Nonframeshift
Missense
Stopgain
Structural variant
Deletion
Splicing
MC1P MC2P MC101P MC7P MC4P MC104P MC103P MC301P MC201P1 MC203P1 MC3P1 MC102P MC5P MC206P MC106P
MC101R MC1R MC207R MC2R MC6R1 MC4R1 MC202R MC106R MC103R MC301R MC102R MC5R1 MC3Ri MC8R MC206R MC105R

Figure S2. B cells in MCL patients. Related to Figure 2.
(**A**) UMAP of B cells after removing the human leukocyte antigen and immunoglobulin genes, colored according to the malignant type (left), patient (middle), and sample (right). (**B**) Patient origin of each subcluster. Colors indicate individual patients, consistent with the legend in the middle panel of A. (**C**) The average expression of B-cell markers in nonmalignant B subclusters. **(D)** Highly variable genes (HGVs) across tumors with unsupervised clustering of samples. FindAllMarkers was used to identify HVGs with the following criteria: minimal cell percentage > 0.1, fold change > 1.5 and p.adjust < 0.05. Each column represents a sample, and all the samples were clustered. Each row represents a gene, and the genes related to lymphomagenesis are labeled and colored according to enriched tissues (pink represents bone marrow, green represents non-bone marrow). **(E)** Identification of B-cell subtypes in normal bone marrow and reactive lymph node samples. (**F**) Differentially expressed genes (DEGs) between malignant B cells and normal B cells from matched tissues. Given that MCL tumor cells are thought to originate from naïve B cells or memory B cells, we performed tissue-specific comparisons: malignant cells in BM samples were analyzed against normal BM-derived naïve B cells, while LN tumor cells were compared with normal B cells from RLN (germinal center B cell and plasma cells were excluded). Red indicates upregulated genes in malignant cells, and blue indicates downregulated genes in malignant cells (fold change > 1.5 and p.adjust < 0.05). The common upregulated DEGs and downregulated genes are labeled. **(G)** DEGs were identified between paired primary and relapse samples from individual patients using pseudobulk RNA-seq data. **(H)** Kaplan–Meier survival analysis of DEGs in an independent MCL validation cohort. The p value was calculated from the Log-rank test. (**I**) Comparison of gene expression between wild-type (WT) and mutant (MUT) high-frequency mutated genes in pseudobulk MCL samples. The dashed line represents the average expression of nonmalignant B cells in MCL samples. The p value was calculated from the Mann–Whitney U test. (**J**) Association between gene alterations and meta-program expression in MCL. The p value was calculated from the Mann–Whitney U test. (**K)** Genes with recurrent structural variants or mutations in primary or relapsed MCL tumors. We compared the mutational frequencies between the primary and relapse groups by including initial samples taken from primary and relapse patients. The genes mutated in at least two patients with primary MCL or with relapsed MCL are displayed. The percentage was the frequency of nonsilent mutations in the primary or relapse patient groups. Structural variants in *CCND1* represent *IGH-CCND1* translocations.

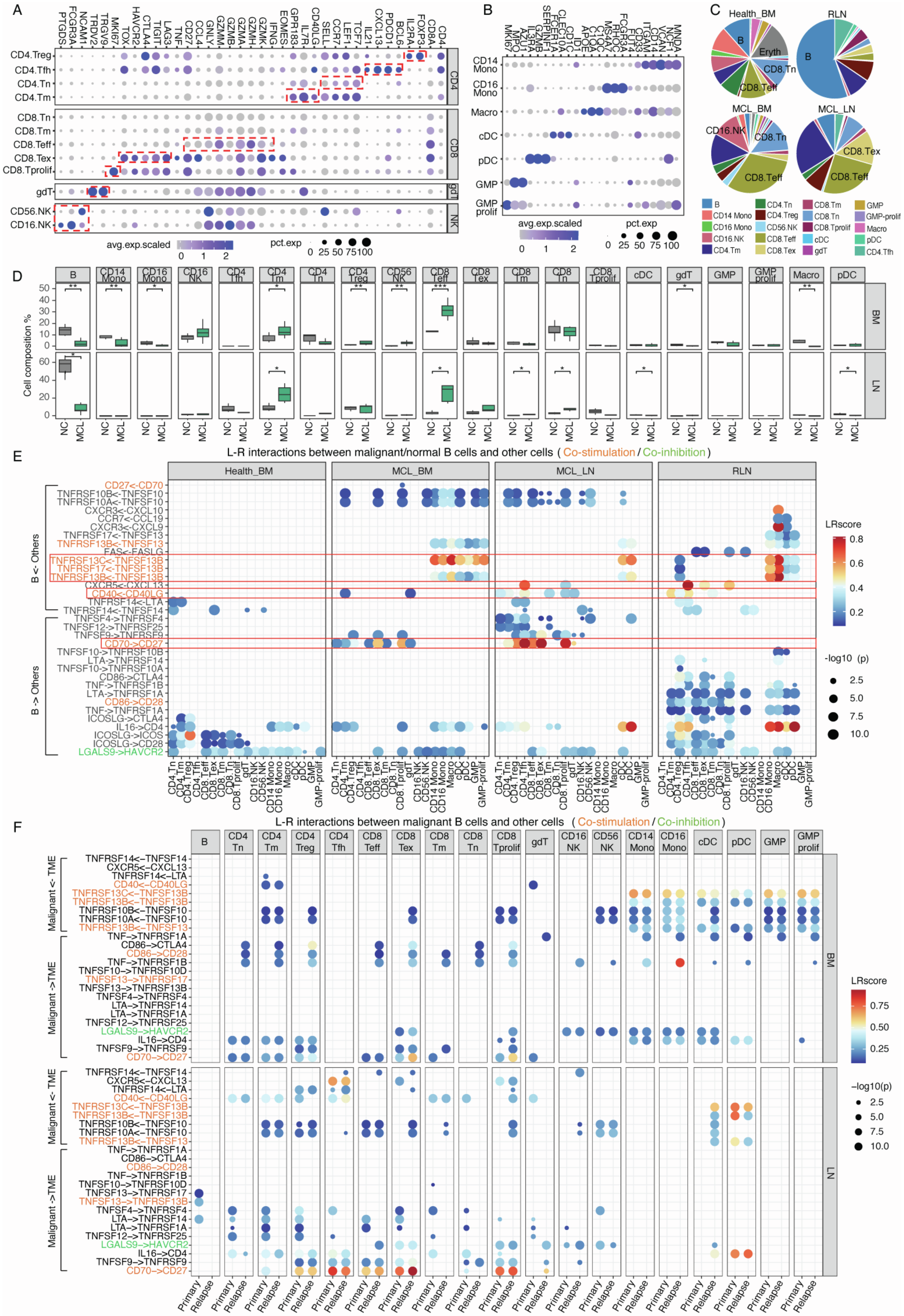

Figure S3. The tumor microenvironment in MCLs. Related to Figure 3.

(A) Dot plot showing marker gene expression across defined T and NK cell clusters. **(B)** Dot plot showing marker gene expression across defined myeloid cell clusters. **(C)** Comparison of the cell population in normal controls and the TME composition in MCLs. Cell populations exceeding 10% of the total composition are labeled. **(D)** Comparison of the relative abundance of each cell subtype between normal controls and MCLs. The p value was calculated from the Mann–Whitney U test. **(E)** Interactions between malignant cells and TMEs in MCL tumors and interactions between B cells and other cells in normal controls. **(F)** Interactions between malignant cells and TME cells at different sampling times in different tumor tissues. One bone marrow control enriched with B cells was excluded from the analysis of cell composition.

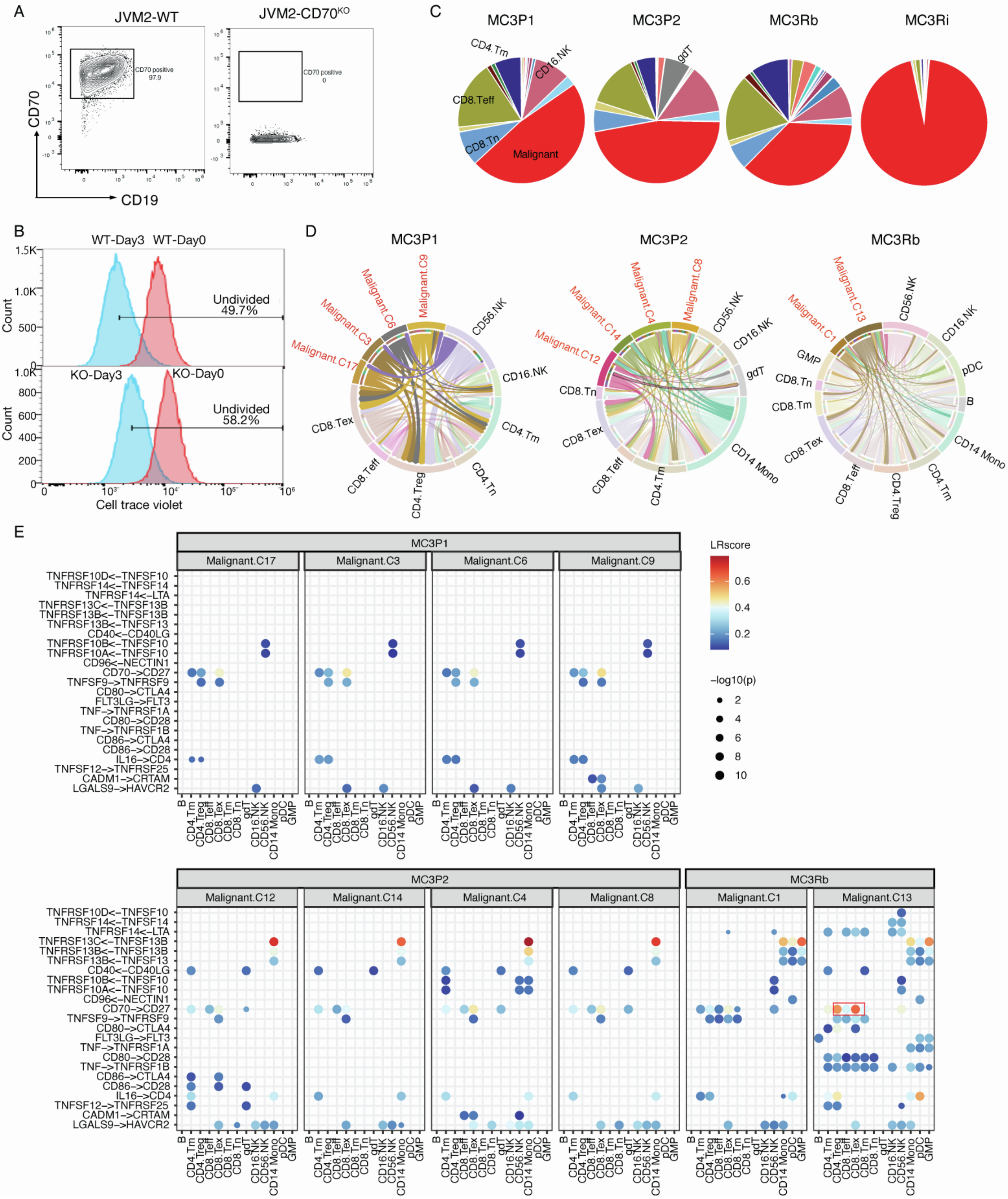

Figure S4. Impact of CD70 expression on MCL proliferation and increased CD70-CD27 interaction between MCL and TME cells in the relapsed tumor of patient MC3. Related to Figure 3 and Figure 6.

(A) Flow cytometry plots of CD70 expression in JVM2 wild-type (JVM2-WT) cells and JVM2-CD70^{KO} cells. (**B**) Proliferation of JVM2-WT and JVM2-CD70^{KO} cells after 3 days in culture. Cell proliferation was assessed using a CellTrace Violet fluorescent dye-based assay, with day 0 undivided cells serving as the baseline control. **(C)** Pie chart showing the cell type composition of each tumor sample. **(D)** Chord diagram showing the crosstalk between cells in each sample. The link width reflects the frequency of interaction between communicating cell types. Only cell subtypes with a cell proportion exceeding 1% are shown. **(E)** Interactions between malignant subclusters and infiltrating immune cells.

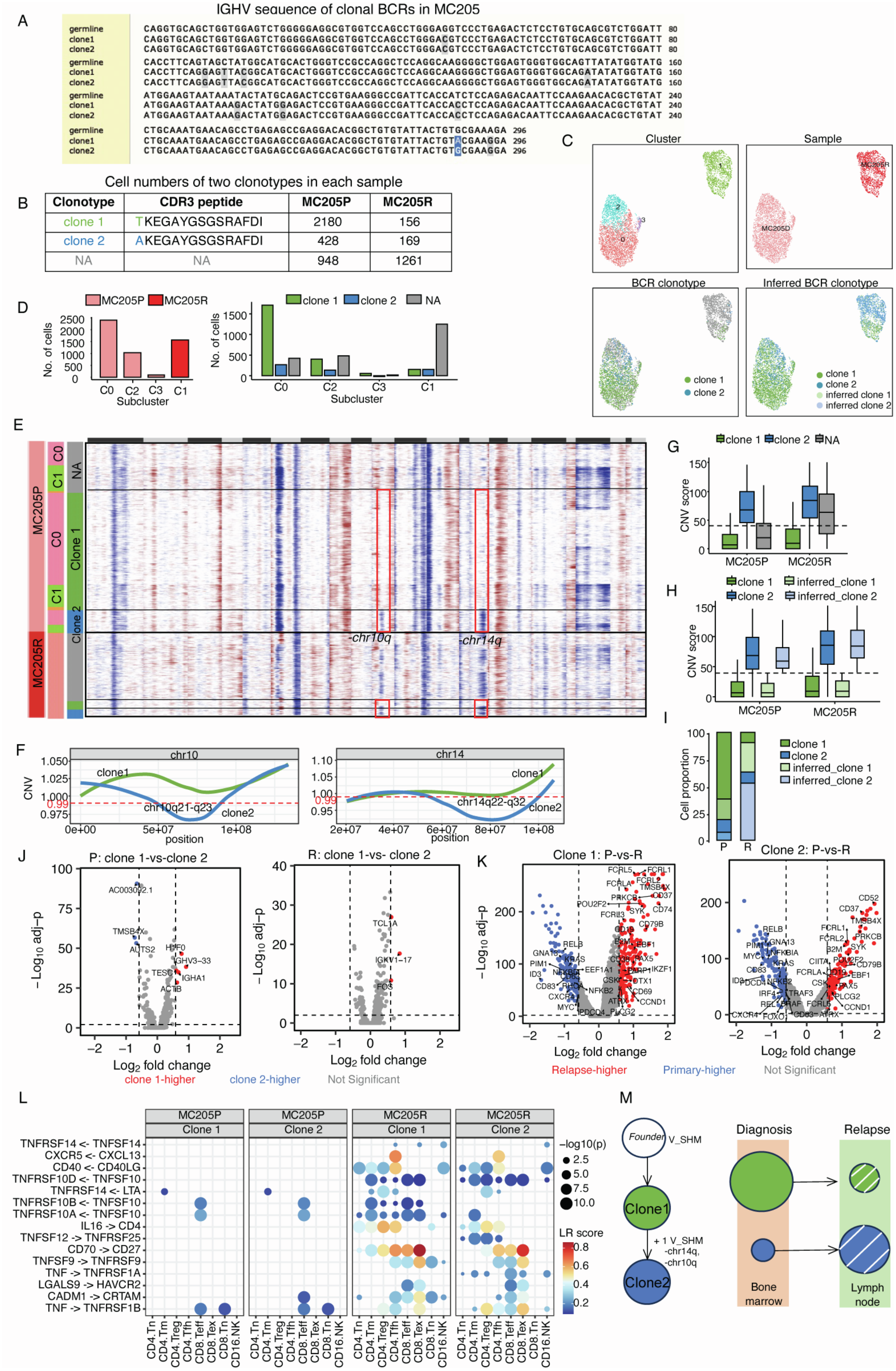
A
IGHV sequence of clonal BCRs in MC205
germline
clone1
clone2
B
Cell numbers of two clonotypes in each sample
Clonotype | CDR3 peptide | MC205P | MC205R
clone 1 | TKEGAYGSGSRAFDI | 2180 | 156
clone 2 | AKEGAYGSGSRAFDI | 428 | 169
NA | NA | 948 | 1261
C
Cluster
Sample
MC205R
MC205D
BCR clonotype
clone 1
clone 2
Inferred BCR clonotype
clone 1
clone 2
inferred clone 1
inferred clone 2
D
MC205P
MC205R
No. of cells
Subcluster
clone 1
clone 2
NA
E
MC205P
MC205R
Clone 1
Clone 2
NA
-chr10q
-chr14q
F
chr10
chr14
CNV
clone1
clone2
chr10q21-q23
chr14q22-q32
position
G
CNV score
MC205P
MC205R
H
inferred_clone 1
inferred_clone 2
I
Cell proportion
J
P: clone 1-vs-clone 2
R: clone 1-vs- clone 2
−Log10 adj−p
Log2 fold change
clone 1-higher
clone 2-higher
Not Significant
K
Clone 1: P-vs-R
Clone 2: P-vs-R
Relapse-higher
Primary-higher
Not Significant
L
MC205P Clone 1
MC205P Clone 2
MC205R Clone 1
MC205R Clone 2
TNFRSF14 <- TNFSF14
CXCR5 <- CXCL13
CD40 <- CD40LG
TNFRSF10D <- TNFSF10
TNFRSF14 <- LTA
TNFRSF10B <- TNFSF10
TNFRSF10A <- TNFSF10
IL16 -> CD4
TNFSF12 -> TNFRSF25
CD70 -> CD27
TNFSF9 -> TNFRSF9
TNF -> TNFRSF1A
LGALS9 -> HAVCR2
CADM1 -> CRTAM
TNF -> TNFRSF1B
−log10(p)
LR score
CD4.Tn CD4.Tm CD4.Treg CD4.Tfh CD8.Teff CD8.Tex CD8.Tn CD16.NK
M
Founder
V_SHM
Clone1
+ 1 V_SHM
-chr14q,
-chr10q
Clone2
Diagnosis
Relapse
Bone marrow
Lymph node

Figure S5. Intratumor heterogeneity and tumor evolution from primary to relapsed tumors in MC205. Related to Figure 5.

(**A**) Alignment of IGHV nucleotide sequences identified from the clonal BCR. (**B**) The number of BCR clonotype cells in each sample. (**C**) UMAPs of malignant cells colored according to transcriptional subclusters, samples, detected BCR clonotypes and inferred BCR clonotypes. (**D**) The cell number (left) and the composition of subclones (right) in each transcriptional subcluster. (**E**) The inferred CNV of malignant cells. The cell identities (samples, subclusters, and subclones) are annotated on the left. (**F**) The CNV changed along chr10 and chr14 in the two clones. CNV=0.99 was used to identify the deletion region in clone 2. (**G**) CNV score of the deletion region in each tumor clone. A CNV score = 40 was used as a threshold to separate clone 1 and clone 2. (**H**) CNV score of the deletion region after inferring the clone type of cells without BCR detection. (**I**) The composition of subclones (right) in each sample. (**J**) Volcano plot of DEGs from clone 1 versus clone 2 in the primary (left) and relapse (right) samples. (**K**) Volcano plot of DEGs between primary and relapse samples in clone 1 (left) and clone 2 (right). (**L**) L-R interaction between malignant subclones and TME cells. (**M**) The inferred clone evolution from diagnosis to relapse. Shading indicates transcriptional changes.

In patient MC205 (treated with ASCT), approximately 73% and 20% of malignant cells were successfully sequenced with BCR clonotypes in primary and relapse samples, respectively. Two BCR clonotypes (clone 1 and clone 2) were detected, distinguished by one mutation in the CDR3 region that generated peptide changes (panel A-B). At the transcriptional level, four distinct subclusters were identified: three (C0, C2, and C3) exclusive to the primary tumor (from the bone marrow) and one (C1) solely present in the relapsed tumor (from the lymph node) (panel C-D). Both BCR clonotypes were found across all subclusters (panel D, right). Interestingly, clone 2 consistently showed deletions of chr14q22-q32 and chr10q21-q23 in both the primary and relapse samples (panels E-F), suggesting a distinct feature of this clone. To further distinguish clone 2 from clone 1, the CNV score of the two deletion regions in each cell was calculated: 86% of clone 1 had a low CNV score (<40), whereas 81% of clone 2 exhibited a higher CNV score (>40) (panel G). Using this approach, we inferred the clonotypes of malignant cells lacking BCR information (panel H), revealing that clone 1 was predominant in the primary tumor, while the proportion of clone 2 increased from 20% in the primary sample to 64% in the relapse sample (panel I). However, these two clones exhibited relatively similar expression patterns, with only minor differences, including decreased expression of BCR (*IGHV3-33*, *IGKV1-17*), *TCL1A*, and *FOS* in clone 2 in both primary and relapsed samples (panel J). We then compared the transcriptional changes in clones 1 and 2 between the primary and relapsed tumors. In the relapsed tumors, both clone 1 and clone 2 exhibited increased expression of genes involved in BCR signaling (*CD19, CD69, CD79B, SYK, FCRL1, FCRL2*), the IFN-γ response (*SP110*, *CD74*), and antigen presentation (*HLA-DQA1, B2M, CIITA*) and downregulated expression of genes involved in the NF-κB (*RELB, NFKB2*) and MYC (*MYC*) signaling pathways, with clone 2 additionally showing downregulation of G2M checkpoint genes (*DMD, HIF1A, NOLC1, YTHDC1, SMAD3*) (panel 6K). Similarly, cell–cell crosstalk analysis revealed greater differences between primary and relapse samples than between clone 1 and clone 2 samples (panel L). Notably, malignant cells in the relapse samples strongly interacted with CD8.Tex cells via CD70–CD27, which might promote T-cell exhaustion. In summary, MC205 tumors displayed two subclones with different genetic alterations but without significant transcriptional changes. However, both subclones exhibited convergent changes in transcriptional activity and interactions with the TME from diagnosis to relapse, indicating that tumor cells may migrate into a favorable TME, altering their cell state and leading to disease relapse (panel M).

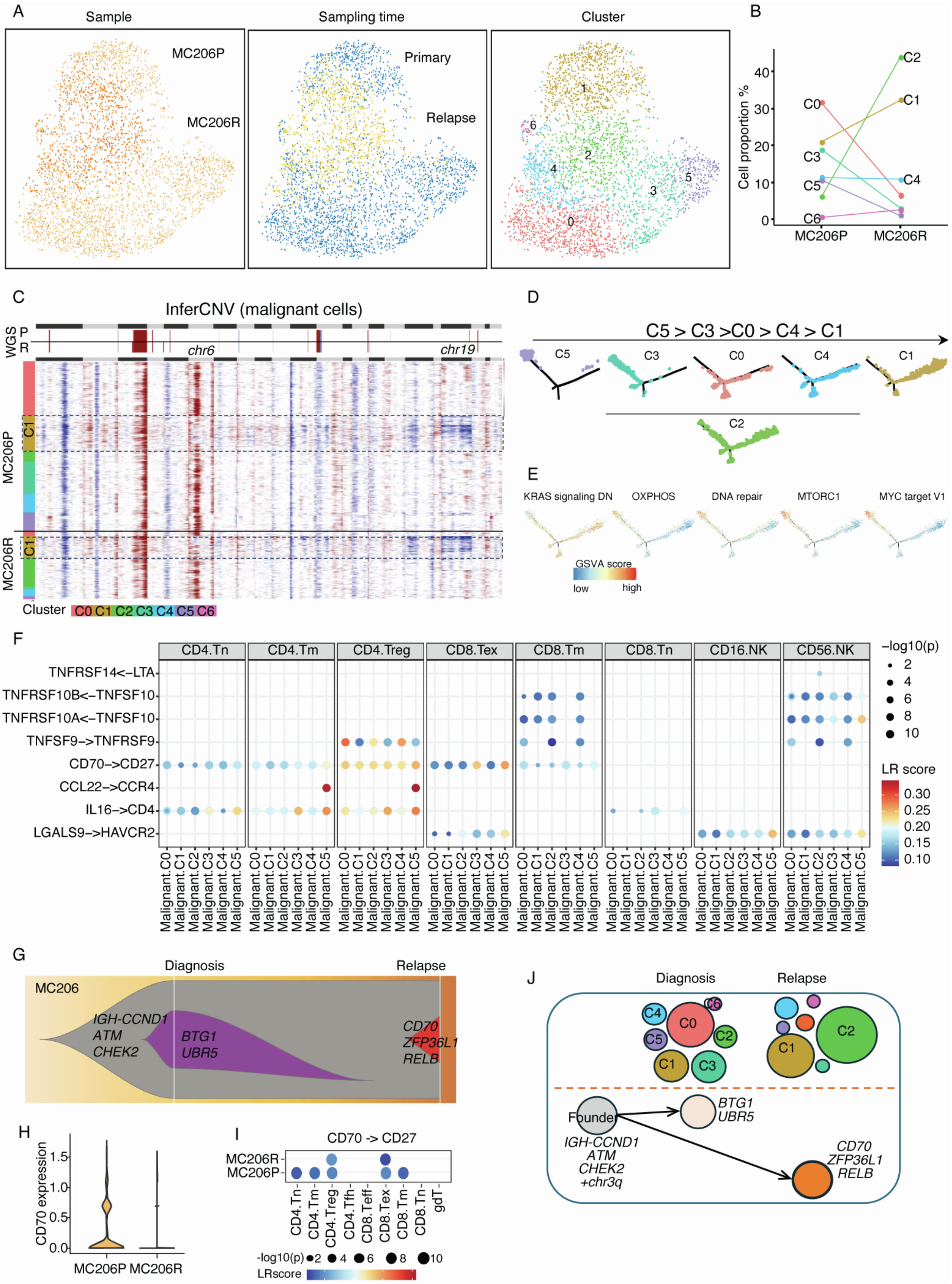

Figure S6. Tumor evolution from diagnosis to relapse in MC206. Related to Figure 5.
(**A**) UMAP of malignant cells colored according to samples, sampling times, and transcriptional subclusters. (**B**) Changes in the malignant subcluster composition between primary and relapsed tumors. (**C**) Large-scale CNVs inferred from WGS (top) and

scRNA-seq (middle) data. Red indicates copy number gain, and blue indicates copy number loss. The root was inferred by starting with primary cells. (**D**) Developmental trajectory representation of malignant cell subclusters inferred by Monocle2. (**E**) The trajectories of malignant cells are colored according to the GSVA score of hallmark pathways. (**F**) L–R interactions between malignant subcluster cells and TME cells. (**G**) Fish plot showing patterns of tumor clonal evolution inferred based on somatic mutations according to WGS data. The representative mutated genes are labeled. (**H**) *CD70* expression in primary and relapse samples. (**I**) CD70–CD27 interaction between malignant cells and T cells in primary and relapse samples. (**J**) The inferred clonal evolution from diagnosis to relapse. The top panel illustrates the changes in the transcriptional subclusters, while the bottom panel shows the clonal evolution based on genetic alterations.

MC206 (treated with R-chemotherapy) presented only one unique BCR clonotype and showed similar gene expression patterns between primary and relapsed tumors. This was also supported by a high SI and non-sample-specific cluster (panel A). Seven transcriptional subclusters (C0-C6) were, however, identified from both samples, with an increased prevalence of C1 and C2 in the relapse sample (panel B). Subcluster C1 displayed additional chr19 deletions and no chr6p amplifications, which is distinct from the other six subclusters (panel C). Trajectory analysis revealed subclusters aligning along a path, starting from C5 and sequentially passing through C3, C0, and C4 and ending at C1 (panel D). Additionally, we observed increased *KRAS* signaling in relapsed tumors and decreased activity of the MYC-targeted, mTORC1, OXPHOS, and DNA repair signaling pathways (panel E). Most subclusters exhibited similar cell crosstalk with TME cells, with C5 showing additional interactions with CD4.Treg and CD4.Tm cells via the CCL22–CCR4 complex (panel F). Clonal evolution analysis based on DNA mutations revealed a founding clone with *IGH-CCND1* translocation, and mutations in *ATM* and *CHEK2*, along with a therapy-eradicated subclone carrying *BTG1* and *UBR5* mutations in the primary tumor, and a subclone with *CD70*, *ZFP36L1*, and *RELB* mutations emerged and expanded in the relapsed tumor (panel G). The relapse sample harbored two small insertions in the coding region of *CD70*, which might contribute to decreased CD70 expression and a reduction in the CD70-CD27 interaction in the relapse sample (panel H-I). Combining genetic and transcriptional data suggested that MC206 tumors carried *IGH-CCND1* translocation and mutations in *ATM* and *CHEK2* and developed into different tumor clones with various mutations (*BTG1* and *UBR5*) and/or transcriptional activities. After treatment, some residual tumor cells acquired additional mutations (*CD70, RELB* and *ZFP36L1*), leading to disease relapse (panel J).

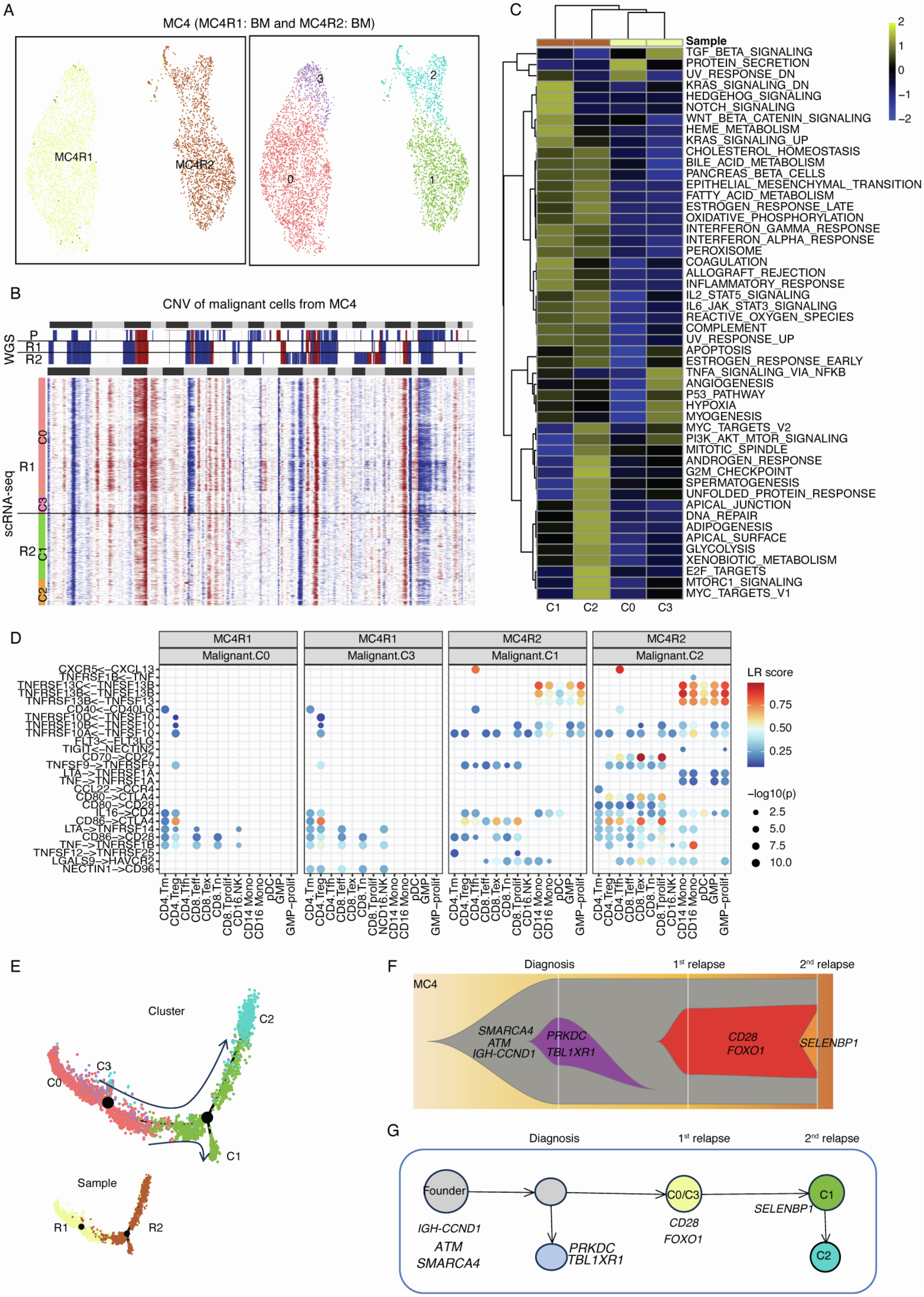

Figure S7. Tumor evolution from the 1st relapse to the 2nd relapse in MC4. Related to Figure 5.

(**A**) UMAP of malignant cells colored according to samples and transcriptional subclusters. (**B**) The inferred CNVs from WGS (top) and scRNA-seq (middle) data. Red indicates copy number gain, and blue indicates copy number loss. (**C**) GSVA of hallmark pathways in each transcriptional subcluster. (**D**) L–R interactions between malignant subcluster cells and TME cells. (**E**) Trajectories of malignant cells, which are colored according to subclusters and samples. The root was inferred from the 1st relapse tumor cells. (**F**) Fish plot showing patterns of tumor clonal evolution inferred based on somatic mutations by WGS data. The representative mutated genes are labeled. (**G**) Inferred clonal evolution in MC4.

In addition to primary-relapse pairs, we analyzed two relapse samples from MC4: one ten years after initial ASCT (MC4R1) and another 6 years after subsequent treatment with R-Bendamustine (MC4R2). Despite both being obtained from bone marrow biopsies, the tumor cells exhibited distinct transcriptional clusters, with four transcriptional subclusters identified (panel A). These subclusters presented similar CNV patterns but exhibited different gene expression pathway enrichment profiles (panel B-C). Cell–cell interaction analysis revealed that subclusters from the same tumor had similar cell crosstalk patterns, with both interacting with CD4.Treg cells via the CD86-CTLA4 axis, while the 2nd relapse led to additional survival support being gained from myeloid cells via BAFF signaling and Tfh cells via CXCR5-CXCL13 interactions (panel D). To infer clonal evolution, trajectory analysis was first performed and showed that the tumor cells from the 1st relapse evolved into C1 tumor cells in the 2nd relapse, which further developed into C2 cells that interacted more with T cells via CD70–CD27 interactions (panel D-E). Moreover, we were able to perform WGS on an additional DNA sample collected at diagnosis and showed that the primary clone, which carried *PRKDC* and *TBL1XR1*, was eliminated by ASCT treatment (panel F). However, some of the founder clone cells developed into relapsed tumors via the acquisition of mutations in *CD28* and *FOXO1*. Subsequently, the tumor clone accumulated the *SELENBP1* mutation, gained survival signals from myeloid cells, and differentiated into a subclone that triggered T-cell exhaustion via CD70 (panel F-G). These data suggest that in MC4, the inferred dominant clone in the primary tumor was eliminated by the treatment, but a subclone surged during the first relapse and developed in the second relapse via linear clonal evolution.

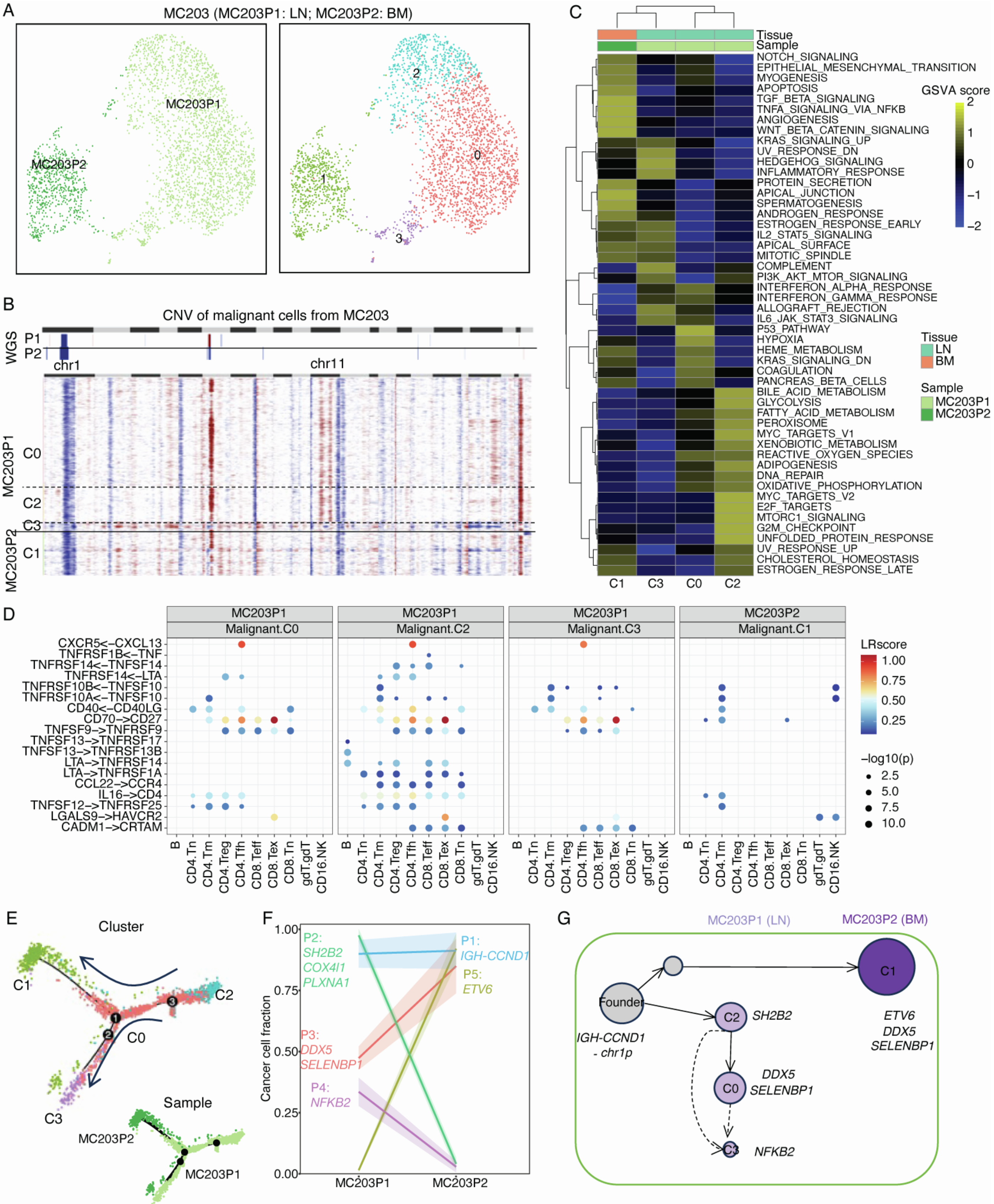

Figure S8. Tumor evolution in the untreated patient MC203. Related to Figure 5.
(A) UMAP of malignant cells identified from the tumors of patient MC203. (**B**) The inferred CNVs from WGS (top) and scRNA-seq (middle) in MC203 samples. Red indicates copy number gain, and blue indicates copy number loss. (**C**) GSVA of hallmark pathways in each transcriptional subcluster. (**D**) L–R interactions between malignant subcluster cells and TME cells. **(E)** Trajectory of malignant cells in MC203, which are colored according to subclusters. The root was inferred from the first sampling of tumor cells. (**F**) Clustering of somatic mutations by cancer cell fraction between two tumor samples was performed by PyClone-VI. The representative mutated genes are labeled. (**G**) Inferred clonal evolution in MC203. The light purple and dark purple represent the subclusters detected in the two tumor samples. The gray represents subclusters undetected by scRNA-seq.

In patient MC203, two tumors were sequentially sampled from the lymph nodes (MC203P1) and bone marrow (MC203P2) at five-year intervals. Four transcriptional subclusters were identified from the two samples, comprising three subclusters (C0, C2, and C3) that were dominant in the lymph node tumor and one subcluster (C1) in the bone marrow tumor (panel A). Subclusters C0 and C2 showed similar CNV patterns, with additional amplifications on chr11 compared with C1, and were distinct from those of subcluster C3 (panel B). These subclusters also presented distinct transcriptional activities: the P53 pathway was enriched in C0, the MYC-targeted and MTORC1 pathways were enriched in C2, the inflammatory response pathway was enriched in C3, and the NOTCH and NF-κB signaling pathways were enriched in C1 (panel C). In addition, subclusters in the MC203P1 tumors interacted with CD8.Tex and CD4.Treg cells via the CD70-CD27 signaling, and interacted with CD4.Tfh cells via CXCR5-CXCL13, while subcluster C2 cells interacted more strongly with T cells via *TNFSF13* and *LTA* interactions (panel D). Trajectory analysis revealed a path starting from C2, branching into C3 or C1, with C0 acting as an intermediate stage (panel E). However, when we further explored clonal evolution using genetic mutations, ClonEvol failed to infer the clonal architecture, indicating that the two tumors may have evolved from separate subclone populations. According to the PyClone-VI results, five clone populations were identified (panel F). All cells from the 1st lymph node tumor (MC203P1) had *IGH-CCND1* (P1) and *SHM2B2*/*COX4I1*/*PLXNA1* (P2) mutations with a CCF of 1, whereas the 2nd bone marrow tumor cells had *IGH-CCND1* (P1) plus *ETV6* (P5) and *DDX5/SELENBP1* (P3) mutations but lacked P2 mutations (panel F). We then correlated the genetic subclones with transcriptional subclusters and observed that genetic clonal evolution (P1+P2 > P3 > P4) mirrored the trajectory branch (C2 > C0 > C3) in the MC203P1 tumor (panel G). These findings suggest the presence of multiple subclones in the primary tumor, with some evolving in the 1st lymph node tumor and others acquiring additional mutations and expanding in the 2nd bone marrow tumor.

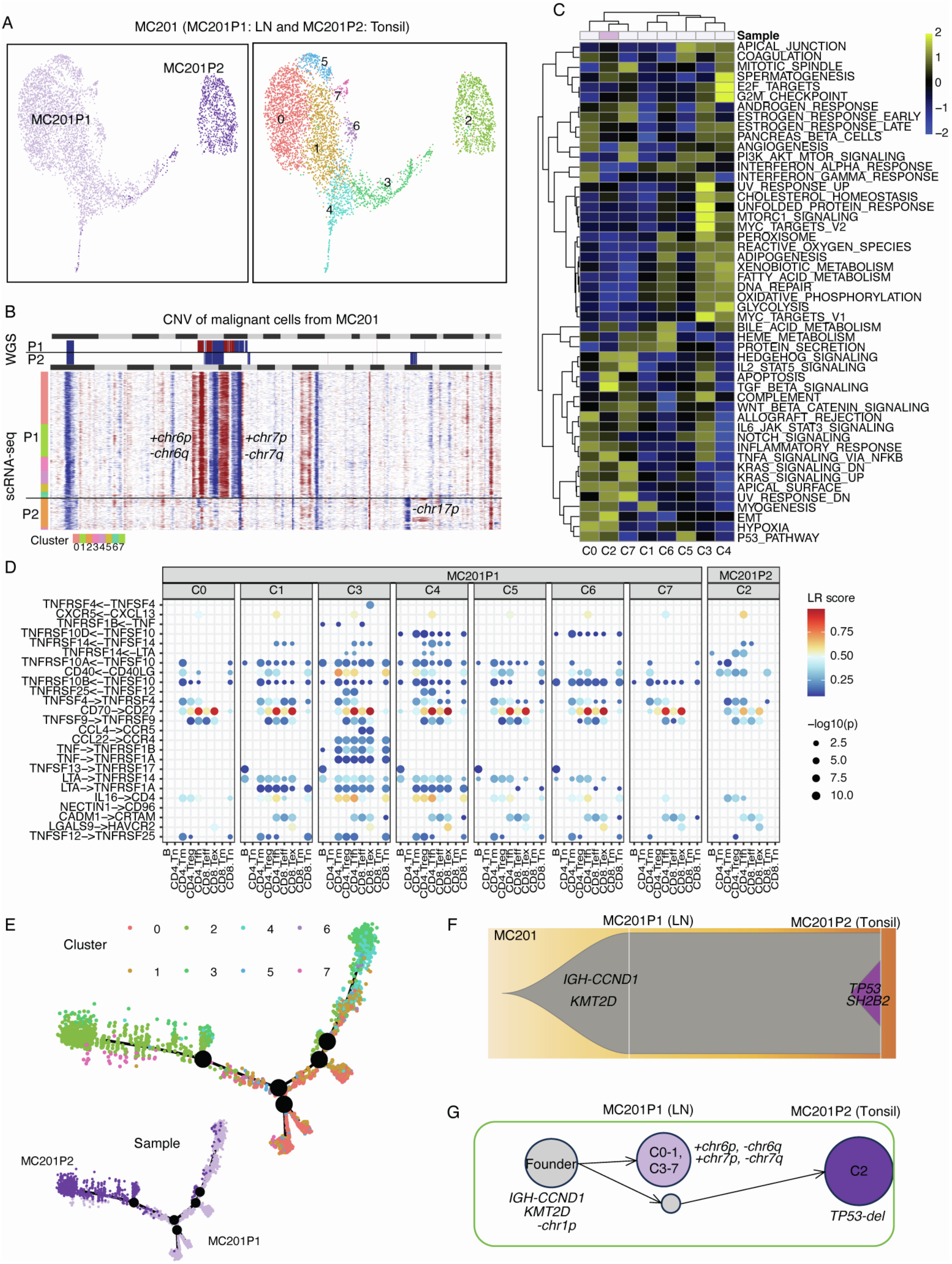

Figure S9. Tumor evolution in the untreated patient MC201. Related to Figure 5.
(**A**) UMAP of malignant cells. (**B**) The inferred CNVs from WGS (top) and scRNA-seq (middle) data. Red indicates copy number gain, and blue indicates copy number loss. (**C**) GSVA of hallmark pathways in each transcriptional subcluster. (**D**) L–R interactions between malignant subcluster cells and TME cells. (**E**) Trajectory of malignant cells, which are colored according to

subclusters and samples. (**F**) Fish plot showing patterns of tumor clonal evolution inferred based on somatic mutations from WGS data. The representative mutated genes are labeled. (**G**) Inferred clonal evolution in MC201.

MC201, without treatment, also exhibited distinct transcriptional subclusters between the two tumor samples. The 2nd tonsil tumor (MC201P2, collected 1.5 years after the 1st lymph node tumor (MC201P1)) was dominated by subcluster C2, whereas the other six subclusters were in the 1st lymph node tumor (panel A). Interestingly, there was a significant difference in the CNV patterns between the two tumors in both the bulk WGS data and the scRNA-seq data. Specifically, +chr6p, -chr6q, +chr7p, and -chr7q were detected in the 1st lymph node tumor, while -chr17q was detected in the 2nd tonsil tumor (panel B). Moreover, subclusters from different tumors also exhibited different enrichments of hallmark pathways (panel C). Further cell crosstalk analysis revealed similar cell–cell interactions among most subclusters, with strong interactions with T cells via the CD70–CD27, IL16–CD4, and CD40–CD40LG complexes (panel D). The trajectory analysis revealed multiple branches in the 1st lymph node tumor and a distinct branch in the 2nd tonsil tumor (panel E). Genetically, the tumors derived from tonsils exhibited additional *TP53* deletions (-chr17p) and *SH2B2* mutations (panel F). This finding suggested that the 2nd tonsil tumor may not have evolved from the 1st lymph node-dominant tumor clone. Instead, it likely evolved from the founding tumor cells that existed before clinical diagnosis, possibly owing to additional *TP53* deletions acquired during progression (panel G).

Table S1. Sample information, sequencing tissue and methods, and classical mutations for each sample, related to Figure 1.

Patient and sample information									Tumor tissue source*			Highgly frequent somatic alterations		
**DonorID**	**Gender**	**Health status**	**Age at Diagnosis**	**Sample**	**Type**	**Time to diagnosis (months)**	**Treatment****	**Data source**	**WGS**	**scRNAseq**	**scBCRseq**	**IGH-CCND1_SV**	**ATM_mut**	**TP53_mut/del**
MC1	male	MCL	77	**MC1P**	Diagnostic	0	/	This study	Tongue base	/	/	yes	yes	yes
				**MC1R**	Relapse	48	CHOP + RadioX	This study	Pleural fluid	Pleural fluid	Pleural fluid	yes	yes	yes
MC2	male	MCL	82	**MC2P**	Diagnostic	0	/	This study	LN	LN	LN	yes	yes	yes
				**MC2R**	Relapse	22	R-CHOP	This study	Tonsil	/	/	yes	yes	yes
MC3	male	MCL	56	**MC3P1**	Untreated	68	watchful waiting	This study	LN	BM	BM	yes	no	no
				**MC3P2**	Untreated	78	watchful waiting	This study	BM	BM	BM	yes	no	no
				**MC3Ri**	Relapse	164	ASCT	This study	Intestine	Intestine	Intestine	yes	no	no
				**MC3Rb**	Relapse	164	ASCT	This study	/	BM	BM	/	/	/
MC4	male	MCL	52	**MC4P**	Diagnostic	0	/	This study	LN	/	/	yes	yes	no
				**MC4R1**	Relapse	121	ASCT	This study	LN	BM	BM	yes	yes	no
				**MC4R2**	Relapse	179	BR	This study	BM	BM	BM	yes	yes	no
MC201	male	MCL	51	**MC201P1**	Untreated	16	watchful waiting	This study	LN	LN	/	yes	no	no
				**MC201P2**	Untreated	33	watchful waiting	This study	Tonsil	Tonsil	/	yes	no	yes
MC202	male	MCL	48	**MC202P**	Diagnostic	0	/	This study	/	LN	LN	/	/	/
				**MC202R**	Relapse	59	ASCT	This study	BM	BM	BM	yes	yes	no
MC203	male	MCL	49	**MC203P1**	Diagnostic	0	/	This study	LN	LN	LN	yes	no	no
				**MC203P2**	Untreated	68	watchful waiting	This study	BM	BM	BM	yes	no	no
MC204	female	MCL	77	**MC204P**	Diagnostic	0	/	This study	/	BM	BM	/	/	/
MC205	male	MCL	59	**MC205P**	Diagnostic	0	/	This study	/	BM	BM	/	/	/
				**MC205R**	Relapse	30	ASCT	This study	/	LN	LN	/	/	/
MC206	female	MCL	78	**MC206P**	Diagnostic	0	/	This study	BM	BM	BM	yes	yes	no

				**MC206R**	Relapse	16	R-FC	This study	BM	BM	BM	yes	yes	no
MC207	male	MCL	65	**MC207R**	Relapse	23	R-FC	This study	LN	LN	LN	yes	yes	yes
MC101	male	MCL	76	**MC101P**	Diagnostic	0	/	This study	LN	/	/	yes	yes	no
				**MC101R**	Relapse	64	R-CHOP, local irradiation	This study	LN	/	/	yes	yes	no
MC102	male	MCL	61	**MC102P**	Diagnostic	0	/	This study	na	/	/	yes	no	no
				**MC102R**	Relapse	23	ASCT	This study	na	/	/	yes	no	no
MC103	male	MCL	69	**MC103P**	Diagnostic	0	/	This study	LN	/	/	yes	no	yes
				**MC103R**	Relapse	56	ASCT	This study	Pleural fluid	/	/	yes	no	yes
MC104	male	MCL	74	**MC104P**	Diagnostic	0	/	This study	BM	/	/	yes	yes	no
MC105	male	MCL	55	**MC105R**	Relapse	7	ASCT	This study	Pleural fluid	/	/	no	no	yes
MC106	male	MCL	67	**MC106P**	Diagnostic	0	/	This study	BM	/	/	yes	no	yes
				**MC106R**	Relapse	28	ASCT	This study	BM	/	/	yes	no	yes
MC5	male	MCL	53	**MC5P**	Diagnostic	0	/	This study	LN	/	/	yes	no	no
				**MC5R1**	Relapse	48	ASCT	This study	LN	/	/	yes	no	no
MC6	female	MCL	44	**MC6R1**	Relapse	30		This study	LN	/	/	yes	yes	no
MC7	male	MCL	41	**MC7P**	Diagnostic	0	ASCT	This study	LN	/	/	yes	yes	no
MC8	male	MCL	62	**MC8R**	Relapse	66	Allotransplant	This study	BM	/	/	yes	no	no
MC301	na	MCL	na	**MC301P**	Diagnostic	0	/	This study	na	/	/	yes	no	yes
				**MC301R**	Relapse	na	na	This study	na	/	/	yes	no	yes
NC01	female	Health	na	**BM_Benrich**	Control	/	/	This study	/	BM	/	/	/	/
NC02	female	Health	66 to 70	**BM1**	Control	/	/	E-MTAB-11536	/	BM	/	/	/	/
NC03	male	Health	51 to 55	**BM2**	Control	/	/	E-MTAB-11536	/	BM	/	/	/	/
NC04	male	Health	71 to 75	**BM3**	Control	/	/	E-MTAB-11536	/	BM	/	/	/	/
NC05	male	Health	61 to 65	**BM4**	Control	/	/	E-MTAB-11536	/	BM	/	/	/	/
NC06	female	Health	56 to 60	**BM5**	Control	/	/	E-MTAB-11536	/	BM	/	/	/	/

NC07	female	Reactive hyperplasia	>60	**RLN1**	Control	/	/	GSE203610	/	LN	/	/	/	/
NC08	male	Reactive hyperplasia	>60	**RLN2**	Control	/	/	GSE203610	/	LN	/	/	/	/
NC09	female	Reactive hyperplasia	>60	**RLN3**	Control	/	/	GSE203610	/	LN	/	/	/	/

*__Sequenced tissue__: BM: bone marrow; LN: lymph node.

** **Treatment:** CHOP: cyclophosphamide, doxorubicin, vincristine, and prednisone; R-CHOP: rituximab plus CHOP; ASCT: Autologous stem cell transplants; BR: bendamustine and rituximab; R-FC: rituximab plus fludarabine and cyclophosphamide.

Table S2. Quality control of WGS samples, related to Figure 2.

Sample	Tumor/Germline type	Clean_Reads	Reads_with_adapter	Reads_with_low_quality	Reads_with_n_rate_exceed	Mapping_Rate	properly_pair_rate	Duplication_Rate	Average_depth(rmdup)	Coverage(≥1X)	Coverage(≥5X)	Coverage(≥10X)	Coverage(≥20X)
MC1P	All tumor	991394838	4317938	0	0	99,88	98,3	13,33	43,71	99,74	99,07	98,12	94,6
MC1R	All tumor	841858370	3616399	0	0	99,85	97,7	11,42	37,87	99,31	98,63	97,88	90,49
MC1PBL	Peripheral blood	626321126	514068	0	0	99,83	96,3	19,72	25,52	99,72	98,93	95,82	74,49
MC2P	All tumor	719916466	430039	0	0	99,86	97	27,4	26,57	99,65	98,84	96,31	77,04
MC2R	All tumor	767707090	3227980	0	0	99,89	97,8	12,4	34,17	99,69	99,06	97,86	88,8
MC2PBL	Peripheral blood	845213088	446265	0	0	99,83	96,3	29,57	30,17	99,74	99,22	97,82	86,05
MC3P1	All tumor	798625856	4266815	0	0	99,89	97,5	10,87	36,09	99,81	99,48	98,73	92,46
MC3P2	All tumor	990784788	915295	0	1,36	99,55	92,3	40,7	27,09	99,87	99,53	97,75	76,63
MC3Ri	All tumor	848574688	2674040	0	0	99,85	98,1	12,12	37,89	99,74	99,26	98,46	92,17
MC3PBL	Peripheral blood	951892200	3332819	0	0	99,9	97	18,46	39,34	99,85	99,56	99,05	94,49
MC4P	All tumor	815060250	2542479	0	0	99,91	98,2	17,62	34,16	99,7	99,11	98,17	90,03
MC4R1	All tumor	742737150	3982850	0	0	99,88	98,5	16,55	31,51	99,75	99,25	97,85	87,18
MC4R2	Sorted B cells	1265715314	1555199	0	1,56	99,63	92,2	44,07	32,67	99,87	99,59	98,49	85,95
MC4PBL	Peripheral blood	935768986	3546578	0	0	99,89	97,9	18,55	38,71	99,8	99,46	98,91	94,19
MC201P1	Sorted B cells	881768976	1038431	0	4,01	99,49	93,8	34,79	26,51	99,87	99,49	96,78	72,87
MC201P2	Sorted B cells	940785716	3426392	0	0,31	99,53	93,9	37,56	27,1	99,87	99,56	97,75	77,31
MC201PBL	Sorted non-B cells	939687274	2835107	0	0,37	99,5	94,2	37,67	27,01	99,87	99,55	97,96	78,59
MC202R	Enriched B cells	909680594	2576935	0	1,97	99,43	96,1	37,91	26,05	99,86	99,5	97,45	74,11
MC202PBL	Enriched non-B cells	928496680	2170380	0	2,31	99,41	94,7	37,2	26,85	99,88	99,56	97,98	78,54
MC203P1	Sorted B cells	974426914	5569223	0	0,23	99,6	94	40,73	26,62	99,85	99,49	97,61	76,1
MC203P2	Sorted B cells	842665164	1101255	0	3,26	99,23	94,9	31,05	26,7	99,84	99,48	97,72	76,91
MC203PBL	Sorted non-B cells	946052700	3326065	0	1,74	99,46	93,8	40,97	25,64	99,86	99,5	97,48	71,85
MC206P	Enriched B cells	919708034	2114878	0	2,39	99,65	95,4	37,77	26,47	99,11	98,76	97,97	76,89
MC206R	Enriched B cells	1028879362	2304553	0	2,51	99,51	95	38,74	29,05	99,15	98,83	98,35	85,99
MC206PBL	Enriched non-B cells	900717276	4646345	0	0,28	99,62	94	35,2	26,99	99,13	98,8	98,24	81,91

MC207R	Sorted B cells	912859940	3151491	0	1,69	99,54	94,1	38,07	26,07	99,87	99,38	96,44	71,81
MC207PBL	Sorted non-B cells	916999674	2092304	0	0,72	99,43	94,3	35,33	27,31	99,86	99,55	98,13	81,12
MC101P	All tumor	887659674	2618748	0	0	99,92	98,3	18,58	36,74	99,81	99,38	98,63	90,29
MC101R	All tumor	713739744	2947838	0	0	99,92	98,1	17,1	30,07	99,76	99,22	97,33	82,5
MC101PBL	Peripheral blood	721520092	2981851	0	0	99,92	97,9	17,3	30,31	99,79	99,34	98,04	86,81
MC102P	All tumor	707503964	3845772	0	0	99,91	96,5	18,33	29,27	99,76	99,3	97,92	85,23
MC102R	All tumor	819936446	4160674	0	0	99,91	97,7	17,6	34,3	99,78	99,4	98,47	90,12
MC102PBL	Peripheral blood	682646346	344587	0	0	99,68	96,7	25,47	25,79	99,69	99,06	96,62	76,74
MC103P	All tumor	704431070	2487475	0	0	99,88	97,1	18,87	28,97	99,82	99,39	97,8	83,55
MC103R	All tumor	739897038	3074849	0	0	99,92	98	15,05	31,92	99,82	99,43	98,23	87,53
MC103PBL	Peripheral blood	782857006	2900301	0	0	99,91	97,8	15,81	33,46	99,82	99,45	98,56	91,09
MC104P	All tumor	796067992	3033111	0	0	99,91	98	16,14	33,92	99,81	99,4	98,56	89,48
MC104PBL	Peripheral blood	822697066	3162232	0	0	99,85	98,1	15,16	35,44	99,8	99,41	98,61	92,28
MC105R	All tumor	829136942	3276072	0	0	99,81	98	16,21	35,27	99,49	98,7	97,55	86,86
MC105PBL	Peripheral blood	719792514	3174538	0	0	99,88	98	14,95	31,09	99,81	99,4	98,17	87,96
MC106P	All tumor	887217388	3113794	0	0	99,91	97,6	18,1	36,89	99,46	98,7	97,97	90,53
MC106R	All tumor	720257970	2755165	0	0	99,91	98,2	16,28	30,64	99,65	98,66	97,31	85,46
MC106PBL	Peripheral blood	874975534	3522182	0	0	99,9	98,4	17,9	36,5	99,8	99,46	98,82	93,23
MC5P	All tumor	959220034	2306575	0	0	99,88	96,2	22,02	37,85	99,8	99,44	98,88	93,98
MC5R1	All tumor	924411966	2405578	0	0	99,9	98,4	19,76	37,72	99,76	99,33	98,7	93,87
MC5PBL	Peripheral blood	1212969312	4506172	0	0	99,91	97,5	21,1	48,57	99,83	99,49	98,95	96,65
MC6R1	All tumor	785024260	2492637	0	0	99,92	98,3	17,7	32,89	99,14	98,49	97,85	92,42
MC6PBL	Peripheral blood	974327506	4020901	0	0	99,92	97,7	20,27	39,47	99,26	98,81	98,46	96,79
MC7P	All tumor	859010296	3693112	0	0	99,89	96,4	19,98	34,81	99,79	99	97,93	89,64
MC7PBL	Peripheral blood	949602548	3544320	0	0	99,89	97,3	22,39	37,38	99,82	99,5	98,9	93,47
MC8R	All tumor	957128152	3887027	0	0	99,92	97,8	18,21	39,77	99,2	98,51	97,96	91,89
MC8PBL	Peripheral blood	846705230	2984525	0	0	99,92	96,9	18,57	34,94	99,81	99,46	98,67	91,6
MC301R	All tumor	812654872	2441239	0	0	99,92	97,2	19,67	33,12	99,79	99,02	97,85	87,19
MC301P	All tumor	736877316	3532554	0	0	99,91	97,2	18,52	30,46	99,51	98,59	96,95	81,17
MC301PBL	Peripheral blood	740857322	4200952	0	0	99,93	97,2	16,61	31,32	99,8	99,4	98,21	87,78
