# Supplementary material for: Tumor evolution and immune microenvironment dynamics in primary and relapsed mantle cell lymphoma
Source: Cell Rep Med. 2025 Aug 27;6(9):102318. doi: 10.1016/j.xcrm.2025.102318 (PMC12490240; doi:10.1016/j.xcrm.2025.102318)
Supplement: Document S2. Article plus supplemental information [file mmc5.pdf]

Cell Reports
Medicine

Article

Tumor evolution and immune microenvironment dynamics in primary and relapsed mantle cell lymphoma

Graphical abstract

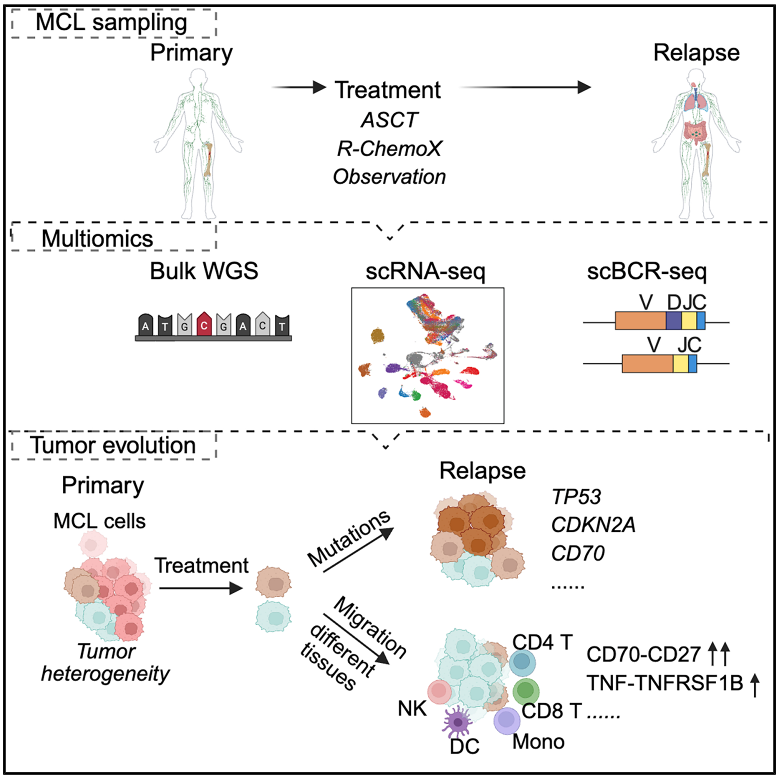

Authors

Hui Wan, Weicheng Ren, Mingyu Yang, ..., Kui Wu, Birgitta Sander, Qiang Pan-Hammarström

Correspondence

wukui@genomics.cn (K.W.),
birgitta.sander@ki.se (B.S.),
qiang.pan-hammarstrom@ki.se (Q.P.-H.)

In brief

Using single-cell RNA sequencing and whole-genome sequencing on paired primary and relapse MCL tumors, Wan et al. demonstrate that genetic evolution, transcriptional heterogeneity, immune interactions, and tumor migration across tissues collectively drive MCL relapse.

Highlights

- Multiomic studies reveal cellular and clonal dynamics in primary and relapsed MCLs
- Significant intratumor heterogeneity is already present at diagnosis
- CD70-mediated signaling may contribute to disease progression and relapse
- Clonal evolution and remodeling of the tumor microenvironment contribute to MCL relapse

Wan et al., 2025, Cell Reports Medicine *6*, 102318
September 16, 2025 © 2025 The Author(s). Published by Elsevier Inc.
https://doi.org/10.1016/j.xcrm.2025.102318

CellPress

Cell Reports Medicine

CellPress
OPEN ACCESS

Article

Tumor evolution and immune microenvironment dynamics in primary and relapsed mantle cell lymphoma

Hui Wan,[1,13] Weicheng Ren,[1,13] Mingyu Yang,[1,2] Man Nie,[3] Agata M. Wasik,[4] Likun Du,[1] Leire de Campos-Mata,[1] Rui Sun,[1] Zhiliang Bai,[5] Archibald Enninful,[5] Yating Wang,[1] Mattias Berglund,[6] Rose-Marie Amini,[6] Xiaobo Li,[2] Chunli Yang,[7,8] Xiaofei Ye,[9] Zhi-Zhang Yang,[10] Stephen M. Ansell,[10] Dongbing Liu,[2,11] Mirjam van der Burg,[12] Rong Fan,[5] Kui Wu,[2,11,*] Birgitta Sander,[4,*] and Qiang Pan-Hammarström[1,14,*]

[1]Division of Immunology, Department of Medical Biochemistry and Biophysics, Karolinska Institutet, Stockholm, Sweden
[2]Guangdong Provincial Key Laboratory of Human Disease Genomic, Shenzhen Key Laboratory of Genomics, BGI Research, Shenzhen, China
[3]Department of Medical Oncology, State Key Laboratory of Oncology in South China, Sun Yat-sen University Cancer Center, Guangzhou, China
[4]Division of Pathology, Department of Laboratory Medicine, Karolinska Institutet and Karolinska University Hospital, Stockholm, Sweden
[5]Department of Biomedical Engineering, Yale University, New Haven, CT, USA
[6]Department of Immunology, Genetics and Pathology, Uppsala University, Uppsala, Sweden
[7]Department of Biotherapy, Cancer Center and State Key Laboratory of Biotherapy, West China Hospital, Sichuan University, Chengdu, China
[8]Department of Medical Oncology, West China Hospital, Sichuan University, Chengdu, China
[9]Kindstar Global Precision Medicine Institute, Wuhan, China
[10]Division of Hematology and Internal Medicine, Mayo Clinic, Rochester, MN, USA
[11]BGI Genomics, Shenzhen, China
[12]Laboratory for Pediatric Immunology, Department of Pediatrics, Willem Alexander Children's Hospital, Leiden University Medical Center, Leiden, the Netherlands
[13]These authors contributed equally
[14]Lead contact
*Correspondence: wukui@genomics.cn (K.W.), birgitta.sander@ki.se (B.S.), qiang.pan-hammarstrom@ki.se (Q.P.-H.)
https://doi.org/10.1016/j.xcrm.2025.102318

SUMMARY

Mantle cell lymphoma (MCL) is a rare but often aggressive type of B cell lymphoma with a high risk of relapse. To explore intratumoral clonal diversity and tumor evolution related to disease relapse, we integrate single-cell RNA and B cell receptor sequencing with whole-genome sequencing in 20 diagnosed/untreated and/or relapsed samples from 11 MCL patients. Our results reveal significant intratumor heterogeneity in MCL already at diagnosis. We further show that the evolutionary paths during disease progression for each patient are unique, where minor clones present at diagnosis may acquire different mutations and copy-number variations and/or migrate to various microenvironments. Despite significant interpatient heterogeneity, recurrent genetic and transcriptomic changes in tumor cells affecting key signaling pathways, along with alterations involved in the tumor microenvironment, are also observed during disease progression. Taken together, our findings elucidate the diverse and dynamic tumor-immune evolution processes associated with disease progression and relapse in MCL.

INTRODUCTION

Mantle cell lymphoma (MCL) is a relatively uncommon yet frequently aggressive type of B cell lymphoma, comprising ∼6% of non-Hodgkin lymphomas. MCL patients typically present with enlarged lymph nodes (LNs) and involvement of the skin, gastrointestinal tract, and bone marrow (BM).[1] The common first-line therapy for young and fit patients is chemotherapy regimens with or without rituximab (R), followed by autologous stem-cell transplantation (ASCT) and R maintenance,[2–4] whereas chemoimmunotherapy followed by R maintenance is commonly used in older patients.[5] Recently, novel therapies, including chimeric antigen receptor (CAR)-T cell therapy,[6] Bruton's tyrosine kinase (BTK) inhibitors,[7] BCL2 inhibitors,[8] immunomodulators,[9] and proteasome inhibitors,[10] have been tested, and BTK inhibitors are being added in frontline treatment in some patients.[11] However, most patients still experience disease relapse following almost all therapeutic interventions.[5,12,13] The cellular and molecular mechanisms underlying relapse or resistance to current first-line and additional therapies remain largely unexplored.

MCLs exhibit high genomic instability and genetic heterogeneity. More than 95% of MCL patients exhibit a characteristic *IGH-CCND1* translocation, leading to cyclin D1 overexpression.

© 2025 The Author(s). Published by Elsevier Inc.
This is an open access article under the CC BY license (http://creativecommons.org/licenses/by/4.0/).

Other common alterations in MCL include loss-of-function mutations in *ATM* and *TP53*.[14,15] Additionally, recurrent mutations in genes involved in epigenetic modification (*KMT2D* and *TERT*), the cell cycle (*CDKN2A* and *MYC*), apoptosis (*BIRC3* and *BCL2*), B-cell receptor (BCR)/nuclear factor κB (NF-κB) signaling (*CARD11* and *NFKBIE*), NOTCH signaling (*NOTCH1* and *NOTCH2*), and other genes, such as *UBR5* and *S1PR1*, have been identified.[14,16–22] Other types of genetic alterations, including copy-number variations (CNVs), structural variations, chromothripsis, and chromoplexy, have also been frequently identified.[23,24] For example, copy-number losses of chr17p/*TP53*, chr9p/*CDKN2A*, and chr11q/*ATM* are common and may contribute to the development of MCL.[25]

MCL patients have a high rate of relapse after first-line therapies (40%–60% within 5 years).[26,27] Genetic alterations such as *TP53*/del17p, *NOTCH1/2*, or *CDKN2A*/del9p are linked to a higher relapse risk and inferior outcomes.[28,29] Additionally, somatic mutations in *CARD11* and *S1PR1* are enriched in relapsed samples, although they are nonrecurrent.[16,22] Comparisons of somatic mutations in paired primary-relapsed MCL tumors suggested two scenarios: linear evolution with accumulation of additional mutations in the initial clone[24] and branching evolution where the primary clone may persist and distinct subclones emerge during relapse.[16,30] Some MCL clones may develop drug resistance by overexpressing certain cell-cycle control or antiapoptotic genes[31] or through acquired mutations, such as *BTK* mutations in response to BTK inhibitor treatment.[32]

Beyond intrinsic tumor abnormalities, the tumor microenvironment (TME) is also important in tumor progression and treatment resistance. *Ex vivo* co-culture systems have shown that MCL cells interact with T cells, macrophages, and stromal cells, promoting cell-cycle activation, apoptosis inhibition, and drug resistance.[33] While single-cell RNA sequencing (scRNA-seq) has been used to study tumor heterogeneity in MCLs, previous studies either lacked TME information[34] or analyzed only single relapsed case.[31] Recently, two scRNA-seq studies have investigated poor responses to ibrutinib or CAR-T-cell treatments in MCL patients.[35,36] However, the clonal evolution, molecular mechanisms, and TME underlying disease relapse after current first-line treatments remain largely unexplored at the single-cell level.

To further investigate mechanisms underlying disease heterogeneity, progression, and relapse in MCL, we integrated whole-genome sequencing (WGS), scRNA-seq, and matched single-cell B cell receptor sequencing (scBCR-seq) in paired primary-relapsed samples from patients treated with several first-line therapies, including immunochemotherapy, chemotherapy, and ASCT. We dissected the tumor clone architecture, inferred clonal evolutionary trajectories for each tumor pair, and tracked dynamic changes in tumor-infiltrating immune cells during disease progression and relapse.

RESULTS

Generation of a single-cell atlas for MCL

We recruited 11 patients with conventional MCL (cMCL) for single-cell analysis (Figure 1A). Among them, four (MC3, MC4, MC202, and MC205) received ASCT, four (MC2, MC204, MC206, and MC207) underwent R-chemotherapy, one (MC1) received chemotherapy combined with local radiotherapy, and two (MC201 and MC203) were under watchful waiting (Figure 1A). All treated patients experienced disease progression or relapse within 10 years after initial therapy, with an average relapse time of 4.2 years. In total, 20 tumor samples were collected from different tissues and/or at different time points and subjected to scRNA-seq, scBCR-seq, and matched WGS on bulk tissues (Figure 1A; Tables S1, S2, and S3). Importantly, paired samples of diagnosed/untreated (hereafter referred to as primary) and/or relapsed MCL were collected from seven patients (Figure 1A). As controls, we included scRNA-seq data from one B cell-enriched BM sample from a healthy donor, as well as publicly available data from five BM samples[37] and three reactive lymph node (RLN) samples from normal individuals.[38]

We obtained 98,990 high-quality cells from 20 MCL samples, of which 39,280 had matched BCR data from scBCR-seq (Table S4). For comparison, we integrated these data with control scRNA-seq datasets, generating a total of 122,963 high-quality cells. Next, major cell types were identified, including B cells (51.8%, malignant and nonmalignant), T cells (36.8%), natural killer (NK) cells (5.9%), myeloid cells (4%), and others (<1%) (Figure 1B; Table S4).

To distinguish malignant B cells from nonmalignant B cells, we reclustered all B cells from each patient and annotated subclusters based on the expected features of tumor cells, including clonal BCR expression, a consistent ratio of immunoglobulin (Ig) kappa to lambda light chain (IGK/IGL), and *CCND1* overexpression (exemplified by MC206, Figures S1A–S1E). Subsequently, the CNVs detected from matched WGS data were used for cross-validation, allowing robust characterization of malignant B cells (Figure S1F). This strategy identified 50,054 malignant B cells and 1,981 nonmalignant B cells (Figure 1C). Notably, the inferred CNVs in malignant B cells exhibited distinct patterns among patients (Figure 1D), reflecting interpatient heterogeneity. Overall, MCL tumors comprised diverse cell types, primarily malignant B cells, along with T cells, NK cells, myeloid cells, and nonmalignant B cells (Figure 1E).

Inter- and intrapatient heterogeneity of malignant B cells in MCL

To explore the molecular features of malignant and nonmalignant B cells in MCL, we pooled B cells from all patients and reclustered them, identifying 19 malignant B cell subclusters and three nonmalignant B cell subclusters (Figure 2A). Malignant subclusters were patient specific and further stratified by sampling time (Figure 2A), suggesting substantial tumor heterogeneity both among patients and across longitudinal samples from the same individuals. Removing the human leukocyte antigen and Ig genes resulted in consistent patient-specific clusters (Figure S2A), indicating that these genes did not introduce any bias in the analysis. In contrast, nonmalignant B cells from various patients were closely clustered into three subclusters: C17 with plasma cell features (*CD38* and *XBP1*) and C16/C21 with mixed features of naive (*IGHD* and *TCL1A*), memory (*CD27*, *IGHG1*, and *IGHA1*), and germinal center (GC) (*BCL6*, *MEF2C*, and *LTB*) B cells (Figures S2B and S2C).

CellPress
OPEN ACCESS

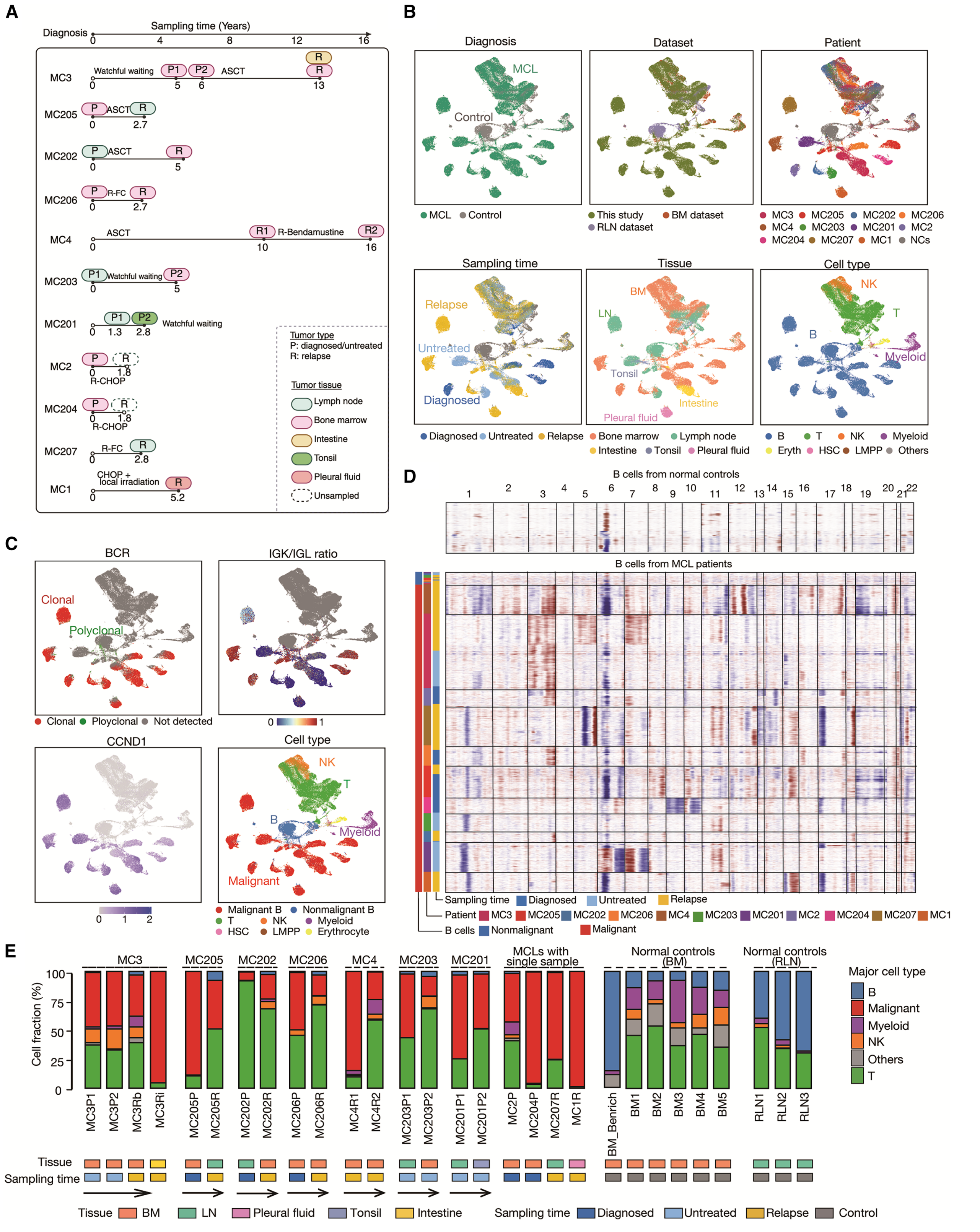

(legend on next page)

To further characterize the transcriptomic heterogeneity of malignant B cells, we performed differential gene expression analysis among malignant B cells from various samples after removing the Ig genes. A set of highly variable genes (HVGs) was identified as characteristic of malignant B cells from individual samples; for example, *MEF2B* was highly expressed in MC3Ri cells and *PIM1* and *MYC* were highly expressed in MC205P cells, whereas *BRAF* and *BCL6* were highly expressed in MC4R1 cells (Figure 2B). Unsupervised clustering of HVGs revealed tissue-associated expression patterns: BM-derived tumor cells presented increased expression of genes related to the cell cycle (*CCND3*, *MYC*, and *CDK14*), BCR/NF-κB signaling (*NFKB2*, *NFKBIA/B/D/Z*, *CARD11*, and *REL*) and B cell development (*BCL6*, *FOXO1*, *IRF4*, *IRF8*, and *GNA13*), whereas tumor cells from other tissues exhibited elevated expression levels of genes linked to DNA repair (*ATM* and *PARP1*), histone modifications (*MEF2B*, *POU2F2*, and *HIST1H2AC*), and BCR/NF-κB signaling (*CD79B*, *PLCG2,* and *SYK*) (Figure S2D). These findings suggest that tumor cells may exhibit distinct transcriptional activities depending on their location, reflecting potential tissue-specific signaling pathways.

Common molecular characteristics of MCLs

To identify common transcriptomic changes in MCL while minimizing tissue-specific effects, we compared the gene expression profiles of malignant cells from individual tumors with those of normal B cells identified from matched tissues—BM or LN (Figure S2E). A total of 1,642 differentially expressed genes (DEGs) were identified, with over 80% showing consistent trends of upregulation or downregulation in tumors derived from both BM and LN samples (Figure S2F). Several genes, including *CCND1*, *BCL2*, *FOXO1*, *CXCR4*, *HIST1H1E*, *CD81*, *ETV6*, *CDK14*, *PRKCB*, *CARD11*, and *GNA12*, were commonly (>5 patients) upregulated in malignant cells.

Next, nonnegative matrix factorization (NMF) was performed to determine the expression programs of malignant B cells from each tumor. Ninety-one expression programs were extracted from 20 MCL samples, with an average of 4.6 programs per sample (range: 2–6) (Table S5). Unsupervised clustering of shared top-scoring genes between programs identified seven common expression programs (meta-programs) involving ribosomes, NF-κB signaling, MYC targets, immune response, cell-cycle control, E2F, and B cell development (Figure 2C; Table S5). A comparison of these meta-programs between paired samples from the same patients revealed that downregulation of NF-κB signaling (meta-program 2) was associated with tumor relapse following ASCT or R-chemotherapy, whereas the MYC-targeted program (meta-program 3) consistently showed a trend of downregulation after ASCT (Figure 2D). Notably, gene set variation analysis (GSVA) of a public microarray dataset (GEO: GSE93291[39]; 123 R-CHOP-treated MCL cases) revealed that decreased NF-κB signaling was significantly associated with poorer patient outcomes, while elevated MYC signaling showed a similar trend (Figure 2E).

To identify potential therapeutic targets related to treatment resistance, we applied a pseudobulk RNA sequencing (RNA-seq) approach to compare gene expression profiles across the four primary-relapse pairs, identifying 15 common DEGs in at least three pairs (Figure S2G). These DEGs are involved in several pathways, including apoptosis (*MYC*, *GADD45B*, and *HSP90AB1*), immune response modulation (*B2M*, *CD52*, and *CD83*), cell migration and invasion (*CRIP1* and *TMSB10*), and transcriptional regulation (*ZFP36L1*, *YBX1*, *DDX21*, and *NCL*). In the GEO: GSE93291 dataset, higher *MYC* and *CD52* expression correlated with poorer patient outcomes, whereas *NCL* and *HSP90AB1* were linked to better survival (Figure S2H).

Although somatic mutation profiles vary among MCLs, *TP53* deletions and *ATM* mutations were frequently observed (Figure 2F). We next investigated the impact of these genetic alterations on gene expression using the pseudobulk RNA-seq method, which revealed that *ATM* alterations were associated with slightly reduced *ATM* gene expression and linked to dysregulation of NF-κB signaling (meta-program 2) (Figures S2I and S2J). To further identify mutations associated with disease progression, we incorporated WGS data from additional 17 tumor samples from a second cohort of MCL patients (*N* = 11; without available scRNA-seq) and compared the mutational frequencies between the primary and relapse groups (Figure S2K). Recurrent alterations (>10%) in primary MCLs included *IGH-CCND1*, *ATM*, *TP53*, *UBR5*, *MEF2B*, *SMARCA4*, *KMT2D*, *NOTCH1*, and *S1PR1*. In contrast, relapse MCLs exhibited higher mutation frequencies in *NOTCH2* (13% vs. 0% in primary) and *BIRC3* (13% vs. 0%). Notably, *NOTCH1* mutations (0% in relapse vs. 20% in primary) were detected in three primary tumors treated with ASCT or CHOP but absent in paired relapse tumors and other relapse cases, suggesting that *NOTCH1*-mutant clones may be more sensitive to chemotherapy.

cMCLs are believed to originate from naive-like B cells, which have not undergone GC reactions and thus have few somatic hypermutations (SHMs) in their variable (V) regions. Indeed, malignant B cells from all MCL patients expressed the IgM isotype and presented a low SHM rate (<2%), except for two patients (MC205: 3.5% and MC2: 2.7%). While different patients had varying VDJ combinations, each displayed consistent VDJ gene usage during disease progression (Table S4), suggesting that clonal expansion arose from a single ancestor cell. Notably, three out of 11 patients expressed the same V genes of both heavy and light chains (*IGHV3-21:IGLV3-19*) in their tumor clones, with slightly longer complementarity determining region

Figure 1. Single-cell atlas of MCL

(A) Overview of the patient cohort with a schematic of treatment and time for sample collection for scRNA-seq.

(B) UMAPs of sequenced single cells. The cells are colored by diagnosis, donor, sampling time, tissue, and major cell type.

(C) Malignant cells were inferred based on clonal BCR, a consistent IGK/IGL ratio, and *CCND1* expression.

(D) Heatmap of the inferred copy-number variations (CNVs) from patients' B cells (middle) across 22 chromosomes compared with B cells from normal controls (top). The color represents the CNV gain (red) and loss (blue).

(E) Fractions of cells from major cell types for each sample. The arrows represent the sampling timeline for the patients. See also Figure S1 and Tables S1 and S4.

CellPress
OPEN ACCESS

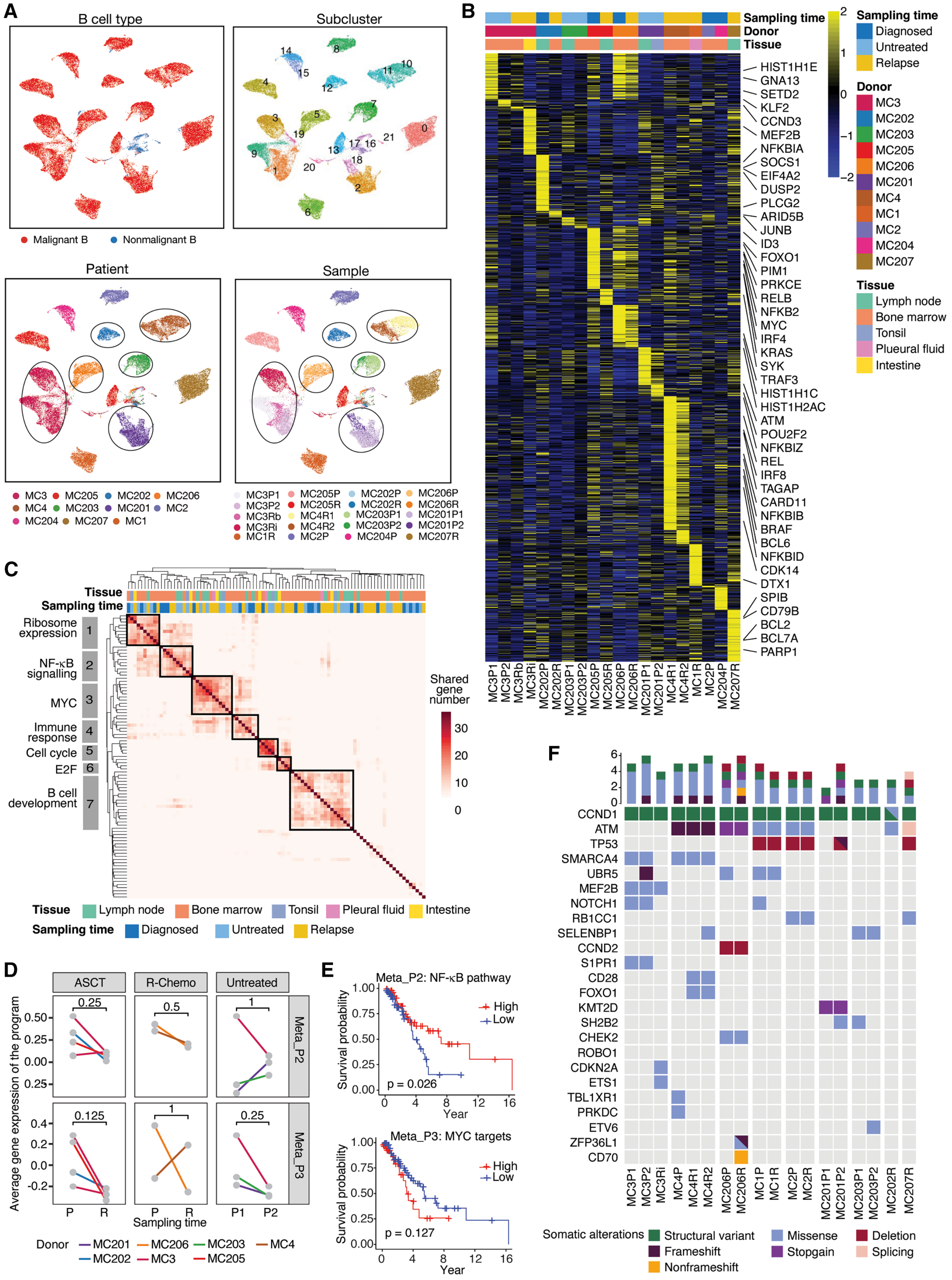

Figure 2. Inter- and intrapatient heterogeneity of MCL

(A) Uniform manifold approximation and projection (UMAPs) of B cells sequenced from all MCL patients, colored by malignant cell type, subcluster, patient, and sample. Tumor cells in the same cluster but from different tumor samples of the same patient are highlighted by circles.

(B) Highly variable genes of malignant cells between tumor samples. The selected lymphoma-related genes are labeled.

(legend continued on next page)

(CDR3) peptides, indicating potential common antigen-driven lymphomagenesis.

In summary, despite significant interpatient heterogeneity in MCLs, common molecular features were identified, including shared transcriptomic changes, frequently mutated genes, and stereotyped BCRs in a subset of tumors. Recurrently mutated genes and dysregulation of key genes and signaling pathways (e.g., MYC-targeted and NF-κB) were also identified during disease relapse.

The TME in MCL

To explore the TME of MCL, we reclustered T cells and NK cells from both the MCL and control samples, totaling 52,507 cells, and identified four major cell types: CD8$^+$ T, CD4$^+$ T, γδ T (gdT), and NK cells (Figure 3A). Using the canonical markers described previously,[40,41] we further annotated the subclusters within these major cell types, identifying 12 cell subtypes. These include naive (CD4.Tn) and memory (CD4.Tm) CD4$^+$ T cells; regulatory (CD4.Treg) and follicular helper (CD4.Tfh) T cells; naive (CD8.Tn), memory (CD8.Tm), effector (CD8.Teff), exhausted (CD8.Tex), and proliferating (CD8.Tprolif) CD8$^+$ T cells; and NK. CD56^{dim}CD16^{hi} (CD16.NK) and NK.CD56^{bright}CD16^{lo} (CD56. NK) cells (Figures 3A and S3A). Similarly, we reclustered the 4,866 myeloid cells and identified seven subclusters, representing CD14$^+$ monocytes (CD14 Mono), CD16$^+$ (FCGR3A$^+$) monocytes (CD16 Mono), conventional dendritic cells (cDCs), plasmacytoid dendritic cells (pDCs), macrophages (Macro), granulocyte monocyte progenitors (GMPs), and proliferating GMPs (Figures 3B and S3B).

We subsequently analyzed the TME composition at the sample level by integrating the nonmalignant B cells identified earlier. Overall, the cell composition of the TME varied among tissues (Figure 3C). BM-derived tumors were enriched in CD8.Teff, CD4.Tm, and CD16.NK cells, while LN-derived tumors mainly consisted of CD4.Tm and CD8.Teff cells but with reduced NK cell infiltration and expanded CD8.Tex cells (Figures 3C and S3C). Notably, the majority (>95%) of the identified myeloid and NK cells were derived from BM samples. Compared with tissue-matched controls, both BM-derived MCLs and LN-derived MCLs presented a significantly increased proportion of CD8. Teff and CD4.Tm cells relative to normal BM and RLN (Figures S3C and S3D), suggesting an immune-modulated TME in MCL.

To further understand the regulatory relationships among different cell subtypes, we investigated ligand-receptor (L−R) pair interactions related to the immune response using previously described methods.[42–45] We identified 32 significantly enriched L-R pairs mediating 1,198 interactions in MCL. Focusing on the interactions involving malignant cells, 14 significantly enriched L-R pairs connected malignant cells with other cell subtypes, including costimulatory CD70-CD27, CD40LG-CD40, TNFSF13B (BAFF)-TNFRSF13B/C, and CXCL13-CXCR5 and coinhibitory LAGLS9-HAVCR2 interactions (Figure 3D). Malignant cells are likely to receive survival signals from T cells via CD40-CD40LG (predominantly with CD4$^+$ T cells) and CD70-CD27 (CD4$^+$ and CD8$^+$ T cells) interactions and from myeloid cells via BAFF-mediated interactions (Figure 3D). These costimulation interactions were significantly stronger in malignant cells than in tissue-matched normal controls (Figure S3E). Indeed, *in vitro* proliferation assays using CD70-expressing and CD70-knockout MCL cells demonstrated that the loss of CD70 reduced MCL cell proliferation (Figures S4A and S4B). Conversely, malignant cells may suppress effector cells (CD8$^+$ T and NK cells) through coinhibitory LGALS9-HAVCR2 (TIM3) interactions (Figure 3D). Notably, increased CD70-CD27 interactions between malignant cells and T cells were predicted in the relapse samples, with varying levels observed across different tumor tissues (Figures 3E and S3F). Additionally, specific interactions, such as interactions with various TME cells via TNF-TNFRSF1B signaling and interactions with myeloid cells via the TNF-TNFRSF1A complex, were exclusively observed in relapsed tumor samples (Figure 3E).

In summary, the TME in MCL predominantly comprises T cells, NK cells, myeloid cells, and B cells, with variations in proportions across different tissues. TME cells potentially support tumor cell survival through CD40- and BAFF-mediated signaling, while tumor cells may induce T cell and NK exhaustion via TIM3 coinhibitory signaling. A significantly increased CD70-CD27 interaction was noted in relapsed samples.

Intratumor transcriptional heterogeneity from diagnosis to relapse

To explore tumor clonal evolution during disease progression, we reclustered malignant B cells from paired samples from each patient. Patient MC202 was excluded from this analysis because of the limited number of malignant cells (n = 66) in the primary sample. As depicted in Figure 4A, within each tumor of an individual patient, malignant B cells were annotated to several different subclusters, representing transcriptomic variations within a tumor. A similarity index (SI)[46] was subsequently calculated by comparing the relative composition of tumor subclusters among different samples within each patient. Five patients, untreated or treated with ASCT or R-chemotherapy, presented a low SI (0.001–0.052), indicating a significant difference in gene expression patterns between tumors during disease progression. In contrast, MC206 treated with R-chemotherapy had a

(C) Identification of common cellular programs in malignant cells. The rows and columns are mirrored and represent the expression programs. The color represents the number of top genes shared between programs. The annotation of meta-programs identified from hierarchical clustering is shown on the left.
(D) Changes in the average gene expression levels in the meta-programs between paired samples from the same patient. The p value was calculated from Wilcoxon signed-rank test.
(E) Kaplan-Meier survival analysis of NF-κB and MYC pathway activity in a public MCL cohort (GEO: GSE93291). The NF-κB pathway gene set was derived from BioCarta, and the MYC pathway gene set was sourced from the Hallmark collection. The p value was calculated via the log rank test.
(F) Nonsilent somatic gene mutations detected by whole-genome sequencing were either present in at least two patients or previously reported in other lymphoma studies. The color indicates the mutation type. Structural variants in *CCND1* represent *IGH-CCND1* translocations. Meta-P, meta-program. See also Figure S2 and Tables S2, S3, S4, and S5.

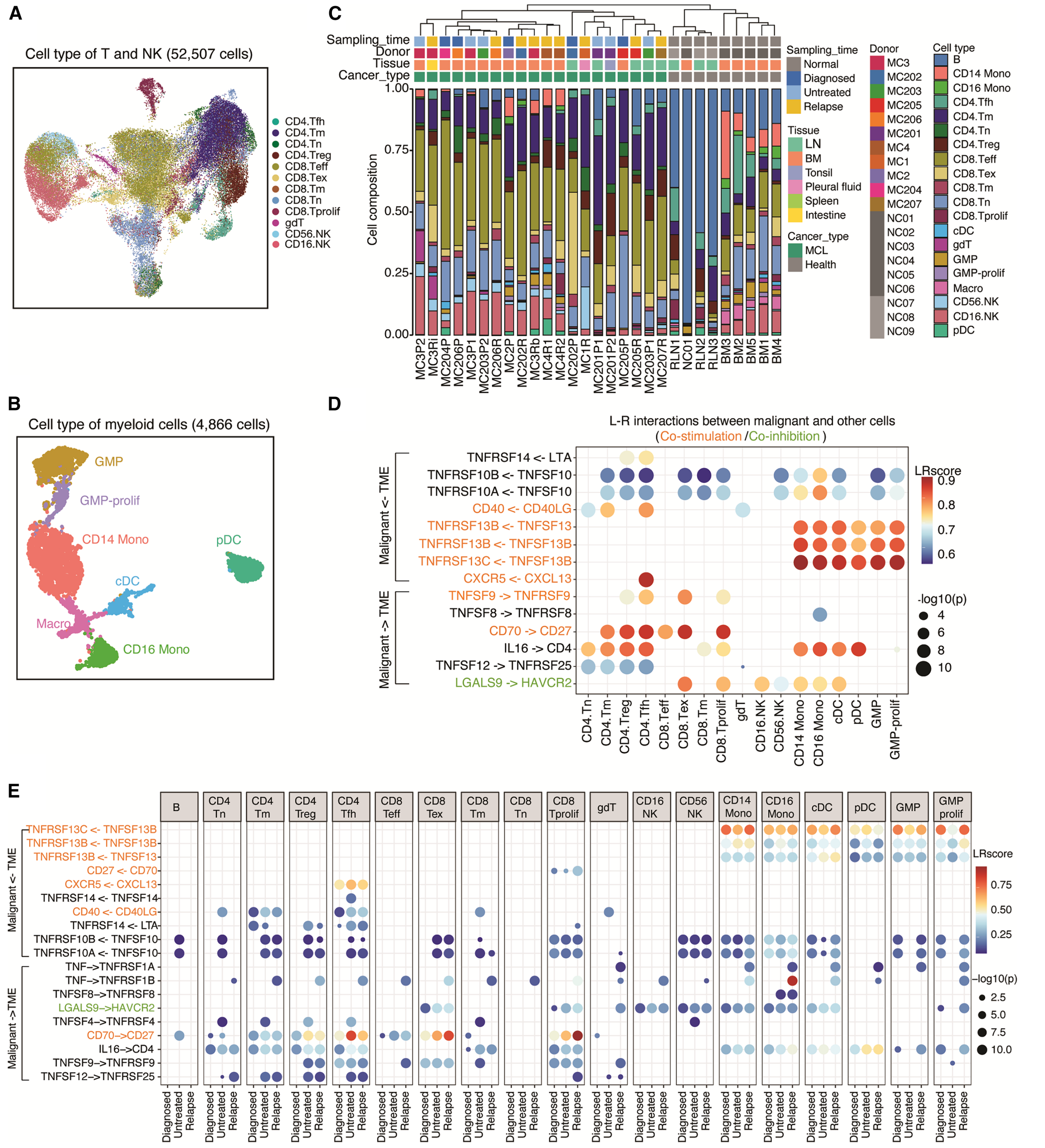

Figure 3. Tumor microenvironment in MCL

(A) UMAPs of T and NK cell subtypes.

(B) UMAPs of myeloid cell subtypes.

(C) TME composition of each sample. The color is the scaled proportion in each sample.

(D) Ligand (L)-receptor (R) interactions between malignant cells and TME cells in MCL patients. L-R pairs are labeled on the left and colored according to their costimulation or coinhibition features.

(E) Interactions between malignant cells and TME cells at different sampling times. *p* values for inferred L-R interactions (D and E) were calculated by CellPhoneDB using permutation testing. See also Figures S3 and S4.

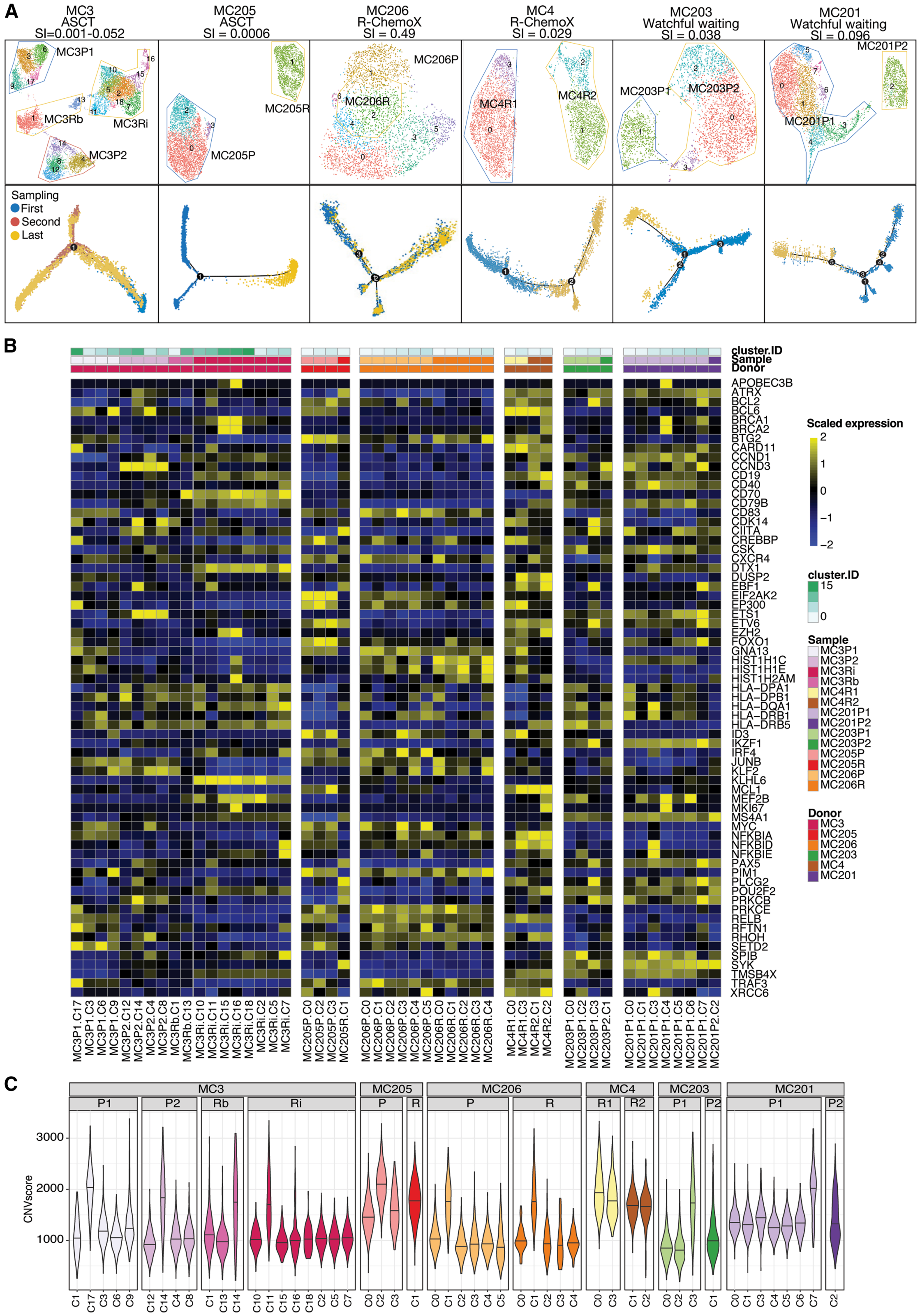

(legend on next page)

relatively high SI (0.49) between the primary and relapsed tumors, suggesting limited changes in the transcriptomic phenotype during disease relapse. Additional pseudotime analysis and trajectory reconstruction of malignant cells for each patient using Monocle2[47] revealed various forms of branch-shaped trajectories (Figure 4A).

Next, we compared the expression levels of key oncogenes across intratumor subclusters to understand the transcriptional heterogeneity within individual tumors. Overall, most of these genes exhibited varying expression levels across individual subclusters within a single patient (Figure 4B). For example, *CDK14* expression varied across subclusters derived from individual tumors (MC203P1, MC3P1, MC3P2, and MC3Rb). *CARD11* was highly expressed in C17 of MC3P1 and C11 of MC3Rb but not in the remaining subclusters of these two tumors. *MYC* exhibited high expression in certain subclusters (C0, C3, and C5) of MC206P. Additionally, intratumor subclusters from each patient displayed different CNV scores (Figure 4C), further highlighting significant intratumor heterogeneity.

Dissecting tumor clone evolution by multilayer data integration in patient MC3

MC3 was initially diagnosed with indolent MCL and did not receive any therapy. Tumor samples were collected from BM and LN biopsies during watchful waiting (MC3P1 and MC3P1_LN), from BM biopsy during disease progression (MC3P2), and from tumor-involved intestine and BM biopsies at relapse (MC3Ri and MC3Rb) (Figure 1A). MC3P1, MC3P2, MC3Ri, and MC3Rb were analyzed by scRNA-seq.

Clonal BCR analysis revealed three unique clonotypes (SHM0, SHM1, and SHM2) with 0–2 mutations in the *IGHV* gene, notably varying in proportion over time (Figures 5A and 5B). SHM0 (MC3P1: 34% and MC3P2: 22%) and SHM1 (MC3P1: 66% and MC3P2: 78%) predominated before treatment, but SHM0 almost disappeared during relapse, whereas SHM1 showed some residual amounts in BM tumors (MC3Rb: 32% and MC3Ri: 0%). Moreover, SHM2, initially constituting a minor fraction (<0.1%), expanded to become the dominant clonotype in the two relapsed tumors (MC3Rb: 67% and MC3Ri: 100%) (Figure 5B). Transcriptional profiling analysis revealed 18 subclusters (C1–C18) across the four samples, each with clear separation by sample and distinct BCR clonotypes (Figure 5C). Specifically, SHM0 was found mainly in C6 and C12; SHM1 was found mainly in C3, C9, C4, C8, and C1; and SHM2 was predominant in relapse-associated subclusters (C13 and most of the subclusters of MC3Ri) (Figure 5D). This correlation analysis provides insights into the evolutionary relationships among these subclusters. For example, since both C6 from the 1st untreated tumor and C12 from the 2nd untreated tumor shared the same BCR clonotype, C12 may have originated from C6, especially considering that C6 was the sole cluster with the SHM0 clonotype in the 1st primary tumor.

We compared the CNV patterns between subclusters from different sampling times and found distinct features in certain subclusters (Figure 5D). Specifically, −chr10p was found in C3, C8, and C1, which share the SHM1 clonotype, whereas −chr15p was found in C9 and C4, which also share the SHM1 clonotype (Figure 5D), suggesting the presence of at least two subclones within the SHM1 clonotype. Moreover, all the subclusters sharing the SHM2 clonotype carried +chr5q and +chr7 but not −chr10p or −chr15p, indicating that cells with the SHM2 clonotype may have originated from an undetected subclone with the SHM1 clonotype by acquiring an additional *IGHV* mutation and amplification of +chr5q and +chr7 (Figure 5D). Additionally, although the BCR clonotype was not detectable (marked in gray in Figure 5D), C17 from the untreated sample (MC3P1), C14 from the progressed tumor (MC3P2), and C11 from the relapsed tumor (MC3Ri) shared the same chr19 deletion, indicating that they may have originated from the same original clone (Figure 5D).

Analyzing the somatic mutational pattern allowed us to further delineate the clonal structure and evolution patterns in longitudinal samples.[48–50] Using the genomic alterations derived from three matched bulk WGS samples (MC3P1_LN, MC3P2, and MC3Ri; MC3Rb, not available), we inferred the clonal population structure based on cancer cell fraction of mutations. We identified a potential founding clone with *IGH-CCND1* translocations and *MEF2B* mutations (marked in gray) and three descending subclones with additional mutations (shown in purple, orange, and red) (Figure 5E). The purple subclone carrying *NOTCH1* and *S1PR1* mutations initially dominated, while the orange subclone, which originated from the purple subclone, further acquired additional mutations (*SMARCA4* and *UBR5)* and expanded during disease progression. However, both subclones were eradicated by therapy and not detected in the relapse sample collected in the intestine (MC3Ri), mirroring the dynamics observed in the transcriptional subclusters with SHM0 and SHM1 clonotypes. The MC3Ri tumor consisted primarily of the red subclone, which acquired *CDKN2A* and *ETS1* mutations from the founding clone, possibly correlating with the emergence of the SHM2 clonotype (Figure 5E).

Overall, we inferred tumor evolution in patient MC3 by incorporating somatic mutations into transcriptional and BCR changes (Figure 5F). The ancestor tumor cells harbored *IGH-CCND1* translocations and *MEF2B* mutations, which gave rise to divergent clones via the acquisition of additional mutations. One lineage developed the SHM0 clonotype harboring *NOTCH1* and *S1PR1* mutations, which was present in the untreated sample at time point 1 (MC3P1, subcluster C6),

Figure 4. Intratumor heterogeneity and tumor evolution during disease progression

(A) UMAPs of tumor cells in each patient with available paired samples (upper) and trajectory analysis between different sampling times (lower). The similarity index (SI) between paired samples is shown in the subtitle.

(B) Heatmap showing the scaled average expression of MCL-related key genes in malignant cells at the intrasample subcluster level. Clusters are first grouped by donor, followed by samples.

(C) CNV scores of the transcriptional clusters in each sample. CNV scores were calculated by quantifying the number of genes with CNVs inferred from the scRNA-seq data, using a threshold of less than 0.95 or greater than 1.05. Each violin represents the distribution of CNV scores for the respective groups, with a line indicating the median. The color represents different samples.

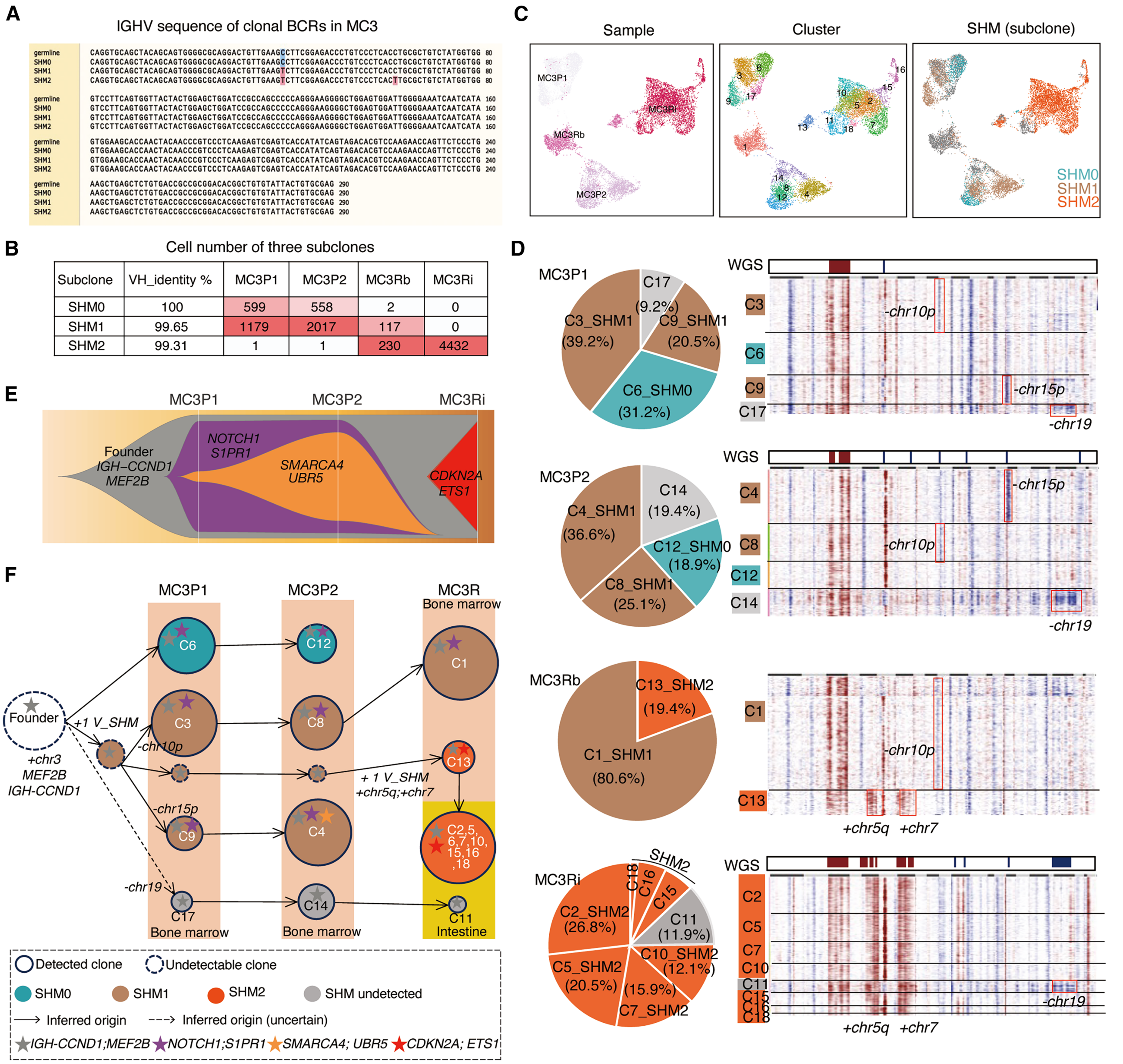

Figure 5. Intratumor heterogeneity and tumor evolution from primary to relapsed tumors in MC3

(A) Alignment of IGHV nucleotide sequences identified from the clonal BCR. The unique clone sequences were named by the SHM number compared with the germline sequence *IGHV4-34* derived from the international ImMunoGeneTics information system (IMGT) database.

(B) The cell numbers of three unique clonotypes in each sample according to unfiltered scBCR-seq data. The cell number is indicated by the percentage of cells in each sample.

(C) UMAPs of malignant cells colored by sample, transcriptional subclusters, and BCR clonotypes.

(D) The composition and CNV features of transcriptional subclusters were identified in each sample. The left pie chart shows the composition of subclusters, and the background of the subcluster is colored according to the BCR clonotype. The right panel shows inferred CNVs (middle) and matched bulk WGS CNVs (top). Red represents copy-number gain, and blue represents copy-number loss. The distinct CNV features are highlighted for each subcluster.

(E) Fish plot showing patterns of tumor clone evolution inferred based on somatic mutations according to WGS data.

(F) Inferred clonal evolution in MC3. One dot represents one subcluster, its size reflects the relative proportion in each stage, and its color represents the BCR clonotype. See also Figures S5–S9 for clonal evolution analysis in other patients.

decreased during progression (MC3P2, subcluster C12), and disappeared during relapse, indicating its eradication by ASCT. Another lineage gained a mutation (SHM1) in the *IGHV* and accumulated mutations in the genome, producing at least three subclones. Two subclones exhibited distinct CNVs with features of either −chr10p or −chr15p, with the first persisting in the

CellPress
OPEN ACCESS

BM-relapsed sample and the latter acquiring *SMARCA4* and *UBR5* mutations during progression and possibly responding to therapy. A third subclone emerged during relapse, acquiring additional mutations (*IGHV* and *CDKN2A*) and amplifications (chr5q and chr7) and became the dominant clone in the relapsed intestine tumor. Additionally, a minor clone of unknown SHM status with a chr19 deletion persisted across all stages (Figure 5F).

The TME compositions of the three MC3 BM-derived samples were largely similar and dominated by CD8.Teff, CD16.NK, CD4. Tm, and CD8.Tn cells, whereas the intestinal sample was composed of >95% malignant cells with minimal immune infiltration (Figure S4C). Despite this, the interaction patterns differed among the samples (Figures S4D and S4E). Four malignant subclusters in the untreated sample MC3P1 exhibited limited interactions with immune cells, whereas those in the progressed tumor MC3P2 showed increased interactions, particularly with CD14^{+} Mono cells via BAFF signaling. In relapsed tumor MC3Rb, malignant cells seemed to receive increased survival support from both CD14^{+} monocytes and myeloid cells through BAFF.

While different malignant subclusters within the same tumor exhibited largely similar cell crosstalk patterns, we observed notable exceptions in MC3Rb. Specifically, the malignant subcluster C13 (SHM2) showed significantly stronger CD70-CD27-mediated interactions with CD4.Treg and CD8.Tex cells compared with subcluster C1 (SHM1) and other malignant subclusters in two primary tumors (Figure 6A). This may reflect elevated *CD70* expression in relapse samples, as the SHM2 clonotype in MC3Rb and MC3Ri tumors exhibited higher *CD70* expression than other clonotypes did (Figure 6B). Consistent with this finding, UMAP visualization revealed that *CD70* was expressed predominantly in malignant cells—with higher levels in MC3Ri—while *CD27* was expressed in both malignant cells and CD4^{+}/CD8^{+} T cells (Figure 6C). Protein-level validation by immunohistochemistry further revealed increased CD70 expression in the relapsed tumor (MC3Ri) relative to the primary tumor (Figure 6D). Furthermore, immunofluorescence staining confirmed high CD70 expression in MC3Ri tumor cells and its colocalization with CD4.Treg and CD8.Tex cells, particularly in perivascular regions (Figure 6E).

In summary, multiple tumor clones coexisted in patient MC3 at different disease stages, and these clones evolved through the acquisition of additional mutations, CNVs, and dynamic interactions with immune cells in the TME.

Dissecting tumor clone evolution during disease progression in MCL patients

We applied the same analytical approach to investigate tumor clonal evolution and tumor-immune cell interactions in the remaining five patients, with detailed findings described in the corresponding figure legends (Figures S5–S9). In one ASCT-treated patient (MC205), the primary tumor harbored two subclones distinguished by one mutation in the CDR3 region and CNVs in chr14q22-q32 and chr10q21-q23 (Figure S5). This pattern of multiple preexisting subclones before treatment resembles that observed in another ASCT-treated patient (MC3). However, MC3 showed the emergence of a dominant subclone at relapse, while both subclones in MC205 persisted in the relapse tumor, albeit with shifted proportions. Notably, both clones exhibited significant changes in transcriptional activity and interactions with the TME from diagnosis to relapse, potentially due to the locations of the tumors. These findings suggest that preexisting subclones were not eliminated by ASCT and subsequently acquired additional mutations or underwent transcriptomic reprogramming upon migrating to different tissues, ultimately contributing to tumor relapse. Among the two patients treated with R-chemotherapeutic agents, MC206 exhibited branching evolution, with the relapse clone originating from ancestral founder cells rather than the dominant pretreatment clones (Figure S6). In contrast, MC4 exhibited linear evolution, in which the second relapse clone emerged from the first through the acquisition of additional mutations (Figure S7). In the two untreated patients (MC201 and MC203), multiple preexisting subclones were identified, further demonstrating distinct evolutionary trajectories across different tissues over time (Figures S8 and S9).

In summary, our analysis demonstrated that MCL tumors harbor multiple subclones at initial diagnosis. While certain clones may be eliminated by first-line treatments, residual tumor cells—either from the same clone or from distinct subclones—may enter a quiescent state. Subsequent acquisition of genetic alterations or favorable changes in the TME enables these residual cell populations to re-enter the cell cycle, ultimately driving disease progression or relapse.

DISCUSSION

In this study, we utilized scRNA-seq and WGS technologies to elucidate tumor heterogeneity, track clonal evolution, and characterize the tissue microenvironment in primary-relapse paired MCL patients following initial first-line treatments. Our results revealed high interpatient, intrapatient, and intratumor heterogeneity, with more than 90 gene expression programs identified in MCL cells. Additionally, we characterized various nonmalignant B cell subclusters and immune cell subclusters from the TME. More than 1,198 potential cell-cell interactions were further predicted, indicating the existence of a complex and dynamic TME in MCL. Furthermore, we elucidated tumor clone structures and clonal evolution by integrating BCR phylogeny, genetic alterations, and transcriptional changes, demonstrating that each patient may follow a unique evolutionary path during disease progression and relapse.

Although patients expressed different tumor BCR clones, the *IGHV3-21:IGLV3-19* combination was notably prevalent in our cohort. This restricted IGHV:IGLV pairing was also commonly found (19%) in other MCL cohorts and was associated with better survival and younger age at diagnosis,[51] suggesting the possible involvement of common antigens in the lymphomagenesis of these patients. Notably, *IGHV3-21* is also enriched in patients with chronic lymphocytic leukemia[52,53] or autoimmune diseases.[54,55] Further analysis of the potentially auto- or polyreactive BCR clones identified in the subset of MCL patients, along with mapping the cross-reactive antigen(s), may help to clarify their roles in MCL development.

Tumor heterogeneity at different levels has significant clinical implications. First, a notable degree of interpatient tumor heterogeneity, characterized by distinct BCR clonotypes, CNV patterns, and transcriptional activities among patients, underscores

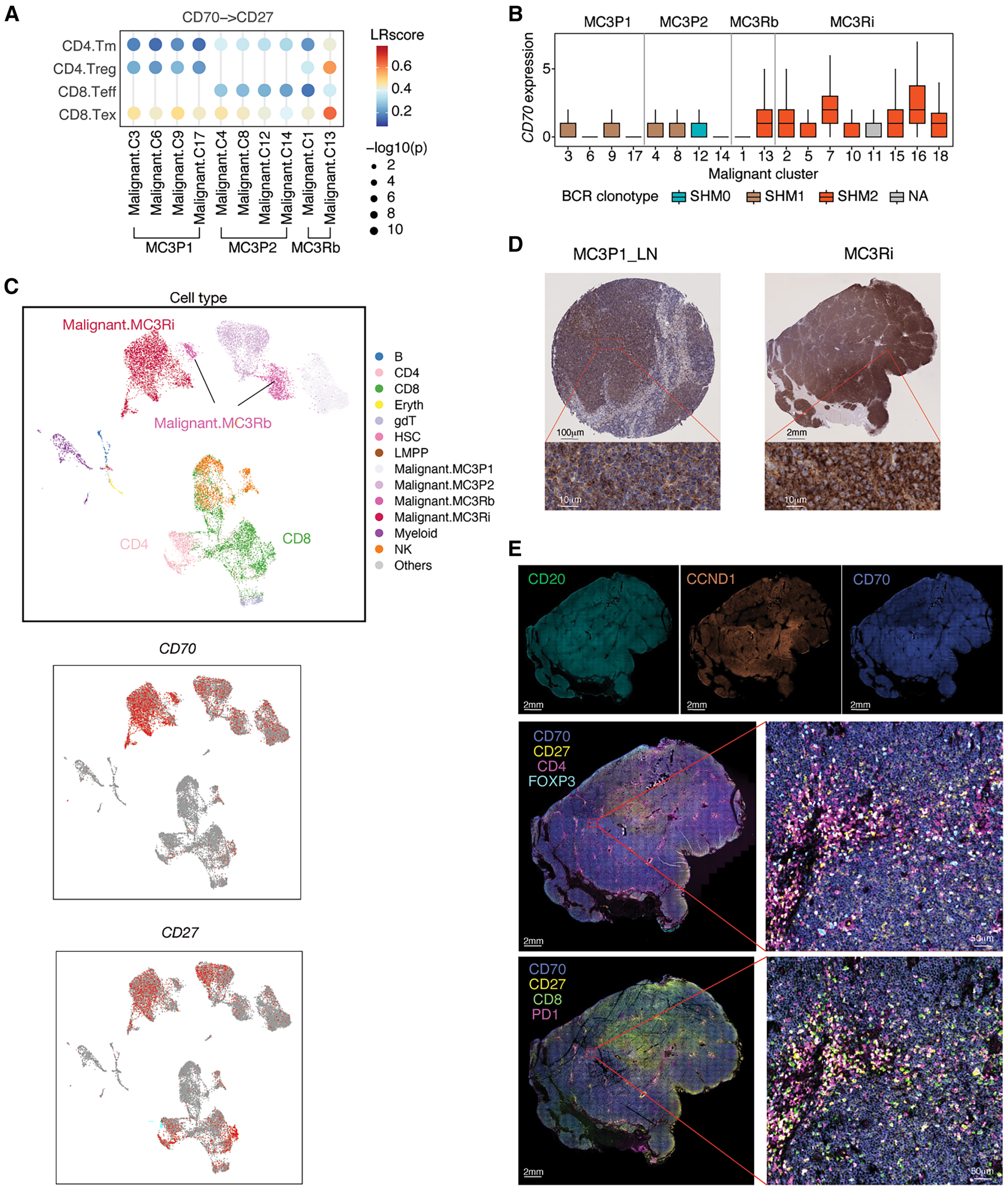

Figure 6. High CD70 expression and strong CD70-CD27 interaction in MC3 relapse tumors

(A) CD70-CD27 interaction between malignant subclusters and T subsets.

(B) *CD70* expression levels across MC3 malignant subclusters. Each box represents the interquartile range (IQR) with a line indicating the median. Whiskers extend to the minimum and maximum values within 1.5 times the IQR, excluding outliers.

(C) UMAP of sequenced single cells in MC3 samples colored by cell type and *CD70* and *CD27* expression.

(D) Immunohistochemistry images of MC3 primary and relapsed tumors stained with an anti-CD70 antibody. Scale bars, 100 μm (top left micrograph), 2 mm (top right micrograph), 100 μm (bottom zoomed-in micrographs).

(E) Multiplex immunofluorescence staining of the MC3Ri sample showing high CD70 expression in malignant cells (row 1: CD20, CCND1, and CD70) and the presence of $CD4^{+}FOXP3^{+}$ and $CD8^{+}PD\text{-}1^{+}$ cells in the tumor microenvironment. Scale bars, 2 mm (overview micrographs) and 50 μm (right zoomed-in micrographs). See also Figure S4.

CellPress
OPEN ACCESS

the necessity for precision medicine. For example, patient MC207, exhibiting increased *PARP1* expression and harboring *ATM* mutations and *TP53* deletions, may benefit from PARP1 inhibitor therapy.[56] In addition, intrapatient tumor heterogeneity, which varied over time and/or between tissue sites, even when samples were collected simultaneously, highlights the limitations of relying on a single tumor biopsy for disease assessment. Furthermore, divergent intratumor subclusters reliant on different genes or signaling pathways may display varying sensitivities to treatments. Therefore, advanced technologies such as panel sequencing, WGS, and single-cell sequencing should be considered for clinical testing to account for tumor features in prognosis and treatment response. For instance, in patient MC3, the predominant tumor subclone (SHM2) in the relapsed tumors showed high expression of *CD70*, which indicates potential responsiveness to CD70-targeted therapies.[57,58] Finally, the significant intratumor heterogeneity at diagnosis, combined with the highly variable evolution trajectories in each patient, may explain why MCL remains difficult to cure with current therapies.

Our study represents a comprehensive single-cell level analysis of the MCL TME, characterizing over 40,000 infiltrating immune cells, with variation observed among tissues. Consistent with previous findings in diffuse large B cell lymphoma (DLBCL) and follicular lymphoma (FL),[42,46] we identified CD40-CD40LG interactions between MCL cells and $CD4^+$ T cells. One notable finding in our study was the strong CD70-CD27 connection between malignant cells and exhausted and proliferating $CD8^+$ T cells as well as between $CD4^+$ regulatory T cells and follicular helper cells, and these interactions increased during disease progression in several MCL patients. The CD27/CD70 pathway is involved in immune regulation and homeostasis, including inducing regulatory functions in $CD4^+$ T cells and contributing to exhaustion in $CD8^+$ T cells.[59–62] High CD70 expression has been linked to increased proliferative capacity and aggressive clinical behavior, particularly in $SOX11^+$ MCL.[63] Moreover, blockade of the CD70/CD27 interaction has been associated with the downregulation of stemness-associated genes in acute myeloid leukemia blasts, suggesting that CD70/CD27 signaling contributes to a more undifferentiated and malignant state.[64] Therefore, targeting the CD27/CD70 signaling pathway or CD70-expressing malignant cells using CAR-T-cell therapy or antibodies represents a promising strategy for therapeutic intervention to address T cell exhaustion and suppression in MCL.[58,60,65] In MC206 patient, however, we observed *CD70* mutations and reduced CD70-CD27 interaction during relapse. This is reminiscent of the findings in DLBCL, where both overexpression of wild-type CD70 and loss-of-function *CD70* genetic alterations were associated with poorer overall survival, but through different immune escape mechanisms (inducing T cell exhaustion or disrupting T cell priming).[66] In the latter case, strategies that restore adequate T cell priming or antibodies targeting CD27 can be considered. Additionally, compared with DLBCL (14% nonmalignant B cells out of all B cells)[42] and FL (7%–16%),[67,68] MCL exhibits relatively low infiltration of nonmalignant B cells (3.8%), potentially limiting the efficacy of PD-1/PD-L1 therapies.[69–71]

Finally, we elucidated the clonal evolution throughout the disease course of MCL. Our multidimensional evidence demonstrated the coexistence of linear and branching evolution during MCL progression. Various tumor subclones may carry distinct mutations across various tumor sites in untreated patients, suggesting that tumor clones may gain survival advantages in different tissues by acquiring additional mutations. Furthermore, not all tumor cells/clones are eliminated by ASCT or R-chemotherapy. The residual tumor cells/clones may evolve by gaining distinct mutations (e.g., *CDKN2A*) or migrate to different tissues/TMEs, leading to tumor growth and disease relapse. These divergent tumor evolutionary trajectories highlight the importance of targeting early, or "truncal," mutations, such as *ATM* and *TP53*, in MCL.

Tumor clones may respond differently to different therapies. In the two patients who underwent ASCT (MC3 and MC205), genetically and transcriptionally different subclones were present before treatment, and some subclones expanded in the relapse tumor through the acquisition of additional mutations (*CDKN2A*, exemplified by MC3) or altering their transcriptomic expression after migrating into different tissues (exemplified by MC205). Among the two patients who received R-chemotherapy, one (MC4) gained additional mutations (*SELENBP1*) in relapse based on pretreatment dominant tumor clones, whereas the other (MC206) acquired mutations (*CD70* and *ZFP36L1*) from a minor and undetected pretreatment tumor clone. A single-cell study of ibrutinib-treated MCL patients also found transcriptionally heterogeneous subpopulations within the same resistant tumor, which gained chr17q amplifications and resulted in elevating *BIRC5* expression, which may lead to tumor cell proliferation.[35] Additionally, fewer effector $CD8^+$ T cells and increased LGALS1-CXCR4/CD69 and TGFB1-CXCR4 interactions between tumor cells and other TMEs are linked to ibrutinib resistance.[35] Another CAR-T-cell therapy study revealed that decreased cytotoxic T cells, increased myeloid-derived suppressor cells, and *TIGIT* overexpression in tumor cells and T cells were associated with MCL relapse after CAR-T-cell therapy.[36] Therefore, dissecting intratumor heterogeneity, identifying key gene alterations/expressions, and examining the aberrant TME are essential for understanding the diverse mechanisms underlying disease relapse in MCL and can guide the development of better and more precise therapeutic strategies.

Limitations of the study

Our single-cell analysis of matched primary and relapsed MCL samples provides a comprehensive view, with longitudinal sampling across diverse first-line treatments offering a representative snapshot of relapse scenarios following conventional therapies. However, several limitations should be acknowledged. First, the relatively small cohort size limits the ability to identify common evolutionary patterns. Second, variations in tissue origin may introduce cofounding tissue-associated signatures that complicate the interpretation of paired primary-relapse comparisons. Third, the heterogeneity of treatment regimens restricts our ability to dissect treatment-specific selective pressures during relapse. Fourth, although our study focuses on conventional regimens, the current primary therapy increasingly incorporates BTK inhibitors, and other targeted therapies and immunotherapies are being evaluated. Expanding future analyses to include these additional regimens will provide more

comprehensive insights into resistance mechanisms and clonal evolution. Lastly, while our data suggested a role for CD70-CD27 interaction in MCL progression, further functional studies, such as co-culturing CD70$^+$ tumor cells with CD27$^+$ T cells along with blockade or stimulation, are needed to validate its mechanistic relevance.

RESOURCE AVAILABILITY

Lead contact

Further information and requests for resources and reagents should be directed to and will be fulfilled by the lead contact, Qiang Pan-Hammarström (qiang.pan-hammarstrom@ki.se).

Materials availability

This study did not generate new unique reagents.

Data and code availability

- The raw scRNA-seq and WGS data have been deposited in the Swedish National Data Service (SND): https://doi.org/10.48723/kfy8-5r05. The data are not publicly available due to Swedish and EU data protection regulations concerning sensitive human data. To request access to the raw data, please submit a request for access via SND and contact the lead contact. Upon receiving a request, the lead contact will coordinate with the research data management office at Karolinska Institutet to review the request and, if approved, notify SND to release the data to the requestor. The requestor must describe the objectives of the research project for which the data will be used. Data access will be considered for research purposes and non-commercial use only. In order to ensure patient privacy, access to personally identifiable information or sensitive clinical information will not be provided, and requests for data access must rigorously adhere to the consent agreements established with study participants. The processed scRNA-seq data have been deposited in GEO under the accession number GEO: GSE303064, and the processed WGS data (all identified somatic mutations) have been deposited in Zenodo : https://doi.org/10.5281/zenodo.16098829.
- The analysis code has been deposited on GitHub (https://github.com/wanhui5867/MCL-scRNAseq).
- Any additional information required to reanalyze the data reported in this paper is available from the lead contact upon request.

ACKNOWLEDGMENTS

This work was supported by the Swedish Cancer Society (Cancerfonden), the Swedish Research Council, Radiumhemmets, the Knut and Alice Wallenberg Foundation, the KI-Mayo Clinic collaborative grant, and the O. E. och Edla Johanssons Vetenskapliga Stiftelse. The scRNA-seq computations were enabled by resources from projects naiss2024-22-1443 and sens2020008 provided by the National Academic Infrastructure for Supercomputing in Sweden (NAISS) at UPPMAX, funded by the Swedish Research Council through grant agreement no. 2022-06725. The WGS computations were enabled by resources provided by the China National GeneBank (CNGB).

AUTHOR CONTRIBUTIONS

Conceptualization: Q.P.-H.; sample collection and resource provision: B.S., A. M.W., M.B., R.F., Z.-Z.Y., and S.M.A.; clinical information interpretation: B.S.; sequencing library: D.L., L.d.C.-M., and A.E.; bioinformatic analysis: H.W., M. Y., Z.B., X.L., and X.Y.; experiments: R.S., Y.W., M.N., M.B., and R.-M.A.; data interpretation and visualization: H.W., W.R., B.S., and Q.P.-H.; writing – original draft preparation: H.W. and W.R.; writing – review and editing: Q.P.-H., W.R., and H.W.; supervision: Q.P.-H., B.S., and K.W.; funding acquisition: B. S. and Q.P.-H. All the authors have read and agreed to the published version of the manuscript.

DECLARATION OF INTERESTS

The authors declare no competing interests.

STAR★METHODS

Detailed methods are provided in the online version of this paper and include the following:

- KEY RESOURCES TABLE
- EXPERIMENTAL MODEL AND STUDY PARTICIPANT DETAILS
  - Patient samples
- METHOD DETAILS
  - Single-cell preparation and fluorescence-activated cell sorting (FACS)
  - scRNA-seq and scBCR-seq library preparation and sequencing
  - scRNA-seq and scBCR-seq data processing and analysis
  - Whole-genome sequencing (WGS) and somatic alteration analysis
  - Bulk transcriptomic sequencing (RNA-seq) and analysis
  - Immunohistochemical (IHC) and multiplex immunofluorescence (mIF) staining
  - CD70 knockout in the JVM2 cell line and MCL cell proliferation assay
- QUANTIFICATION AND STATISTICAL ANALYSIS

SUPPLEMENTAL INFORMATION

Supplemental information can be found online at https://doi.org/10.1016/j.xcrm.2025.102318.

Received: September 30, 2024
Revised: May 15, 2025
Accepted: July 30, 2025
Published: August 27, 2025

REFERENCES

1. Veloza, L., Ribera-Cortada, I., and Campo, E. (2019). Mantle cell lymphoma pathology update in the 2016 WHO classification. Ann. Lymphoma *3*, 3. https://doi.org/10.21037/aol.2019.03.01.
2. Vose, J.M. (2017). Mantle cell lymphoma: 2017 update on diagnosis, risk-stratification, and clinical management. Am. J. Hematol. *92*, 806–813. https://doi.org/10.1002/ajh.24797.
3. Campo, E., and Rule, S. (2015). Mantle cell lymphoma: evolving management strategies. Blood *125*, 48–55. https://doi.org/10.1182/blood-2014-05-521898.
4. Schieber, M., Gordon, L.I., and Karmali, R. (2018). Current overview and treatment of mantle cell lymphoma [version 1; peer review: 3 approved]. F1000Research *7*, F1000 Faculty Rev-1136. https://doi.org/10.12688/f1000research.14122.1.
5. Armitage, J.O., and Longo, D.L. (2022). Mantle-Cell Lymphoma. N. Engl. J. Med. *386*, 2495–2506. https://doi.org/10.1056/NEJMra2202672.
6. Iacoboni, G., Dietrich, S., and Liebers, N. (2023). CAR T-cell therapy versus allogeneic HSCT for relapsed or refractory mantle cell lymphoma. Lancet. Haematol. *10*, e951–e952. https://doi.org/10.1016/S2352-3026(23)00335-6.
7. Song, Y., Zhou, K., Zou, D., Zhou, J., Hu, J., Yang, H., Zhang, H., Ji, J., Xu, W., Jin, J., et al. (2022). Zanubrutinib in relapsed/refractory mantle cell lymphoma: long-term efficacy and safety results from a phase 2 study. Blood *139*, 3148–3158. https://doi.org/10.1182/BLOOD.2021014162.
8. Lin, V.S., Anderson, M.A., Huang, D.C.S., Roberts, A.W., Seymour, J.F., and Tam, C.S.L. (2019). Venetoclax for the treatment of mantle cell lymphoma. Ann. Lymphoma *3*, 4. https://doi.org/10.21037/AOL.2019.03.02.
9. Trneny, M., Lamy, T., Walewski, J., Jurczak, W., Belada, D., Mayer, J., Radford, J., Alexeeva, J., Osmanov, D., Biyukov, T., et al. (2014). Phase II Randomized, Multicenter Study of Lenalidomide Vs Best Investigator's

Choice in Relapsed/Refractory Mantle Cell Lymphoma: Results of the MCL-002 (SPRINT) Study. Blood *124*, 626. https://doi.org/10.1182/blood.v124.21.626.626.

10. Goy, A., Bernstein, S.H., Kahl, B.S., Djulbegovic, B., Robertson, M.J., de Vos, S., Epner, E., Krishnan, A., Leonard, J.P., Lonial, S., et al. (2009). Bortezomib in patients with relapsed or refractory mantle cell lymphoma: updated time-to-event analyses of the multicenter phase 2 PINNACLE study. Ann. Oncol. *20*, 520–525. https://doi.org/10.1093/annonc/mdn656.
11. Dreyling, M., Doorduijn, J., Giné, E., Jerkeman, M., Walewski, J., Hutchings, M., Mey, U., Riise, J., Trneny, M., Vergote, V., et al. (2024). Ibrutinib combined with immunochemotherapy with or without autologous stem-cell transplantation versus immunochemotherapy and autologous stem-cell transplantation in previously untreated patients with mantle cell lymphoma (TRIANGLE): a three-arm, randomised, open-label, phase 3 superiority trial of the European Mantle Cell Lymphoma Network. Lancet *403*, 2293–2306. https://doi.org/10.1016/S0140-6736(24)00184-3.
12. Avivi, I., and Goy, A. (2015). Refining the mantle cell lymphoma paradigm: impact of novel therapies on current practice. Clin. Cancer Res. *21*, 3853–3861. https://doi.org/10.1158/1078-0432.CCR-15-0488.
13. Maddocks, K. (2018). Update on mantle cell lymphoma. Blood *132*, 1647–1656. https://doi.org/10.1182/BLOOD-2018-03-791392.
14. Zhang, J., Jima, D., Moffitt, A.B., Liu, Q., Czader, M., Hsi, E.D., Fedoriw, Y., Dunphy, C.H., Richards, K.L., Gill, J.I., et al. (2014). The genomic landscape of mantle cell lymphoma is related to the epigenetically determined chromatin state of normal B cells. Blood *123*, 2988–2996. https://doi.org/10.1182/BLOOD-2013-07-517177.
15. Mareckova, A., Malcikova, J., Tom, N., Pal, K., Radova, L., Salek, D., Janikova, A., Moulis, M., Smardova, J., Kren, L., et al. (2019). ATM and TP53 mutations show mutual exclusivity but distinct clinical impact in mantle cell lymphoma patients. Leuk. Lymphoma *60*, 1420–1428. https://doi.org/10.1080/10428194.2018.1542144.
16. Wu, C., de Miranda, N.F., Chen, L., Wasik, A.M., Mansouri, L., Jurczak, W., Galazka, K., Dlugosz-Danecka, M., Machaczka, M., Zhang, H., et al. (2016). Genetic heterogeneity in primary and relapsed mantle cell lymphomas: Impact of recurrent CARD11 mutations. Oncotarget *7*, 38180–38190. https://doi.org/10.18632/oncotarget.9500.
17. Rosenquist, R., Beà, S., Du, M.Q., Nadel, B., and Pan-Hammarström, Q. (2017). Genetic landscape and deregulated pathways in B-cell lymphoid malignancies. J. Intern. Med. *282*, 371–394. https://doi.org/10.1111/joim.12633.
18. Pararajalingam, P., Coyle, K.M., Arthur, S.E., Thomas, N., Alcaide, M., Meissner, B., Boyle, M., Qureshi, Q., Grande, B.M., Rushton, C., et al. (2020). Coding and noncoding drivers of mantle cell lymphoma identified through exome and genome sequencing. Blood *136*, 572–584. https://doi.org/10.1182/BLOOD.2019002385.
19. Hill, H.A., Qi, X., Jain, P., Nomie, K., Wang, Y., Zhou, S., and Wang, M.L. (2020). Genetic mutations and features of mantle cell lymphoma: A systematic review and meta-analysis. Blood Adv. *4*, 2927–2938. https://doi.org/10.1182/bloodadvances.2019001350.
20. Bühler, M.M., Martin-Subero, J.I., Pan-Hammarström, Q., Campo, E., and Rosenquist, R. (2022). Towards precision medicine in lymphoid malignancies. J. Intern. Med. *292*, 221–242. https://doi.org/10.1111/JOIM.13423.
21. Meissner, B., Kridel, R., Lim, R.S., Rogic, S., Tse, K., Scott, D.W., Moore, R., Mungall, A.J., Marra, M.A., Connors, J.M., et al. (2013). The E3 ubiquitin ligase UBR5 is recurrently mutated in mantle cell lymphoma. Blood *121*, 3161–3164. https://doi.org/10.1182/BLOOD-2013-01-478834.
22. Wasik, A.M., Wu, C., Mansouri, L., Rosenquist, R., Pan-Hammarström, Q., and Sander, B. (2018). Clinical and functional impact of recurrent S1PR1 mutations in mantle cell lymphoma. Blood Adv. *2*, 621–625. https://doi.org/10.1182/bloodadvances.2017014860.
23. Nadeu, F., Martin-Garcia, D., Clot, G., Díaz-Navarro, A., Díaz-Navarro, A., Navarro, A., Vilarrasa-Blasi, R., Kulis, M., Royo, R., Gutiérrez-Abril, J., et al. (2020). Genomic and epigenomic insights into the origin, pathogenesis and clinical behavior of mantle cell lymphoma subtypes. Blood *136*, 1419–1432. https://doi.org/10.1182/blood.2020005289.
24. Beà, S., Valdés-Mas, R., Navarro, A., Salaverria, I., Martín-Garcia, D., Jares, P., Giné, E., Pinyol, M., Royo, C., Nadeu, F., et al. (2013). Landscape of somatic mutations and clonal evolution in mantle cell lymphoma. Proc. Natl. Acad. Sci. USA *110*, 18250–18255. https://doi.org/10.1073/pnas.1314608110.
25. Royo, C., Salaverria, I., Hartmann, E.M., Rosenwald, A., Campo, E., and Beà, S. (2011). The complex landscape of genetic alterations in mantle cell lymphoma. Semin. Cancer Biol. *21*, 322–334. https://doi.org/10.1016/J.SEMCANCER.2011.09.007.
26. Kumar, A., Sha, F., Toure, A., Dogan, A., Ni, A., Batlevi, C.L., Palomba, M. L.M., Portlock, C., Straus, D.J., Noy, A., et al. (2019). Patterns of survival in patients with recurrent mantle cell lymphoma in the modern era: progressive shortening in response duration and survival after each relapse. Blood Cancer J. *9*, 50. https://doi.org/10.1038/s41408-019-0209-5.
27. Eskelund, C.W., Dimopoulos, K., Kolstad, A., Glimelius, I., Räty, R., Gjerdrum, L.M.R., Sonnevi, K., Josefsson, P., Nilsson-Ehle, H., Bentzen, H.H. N., et al. (2021). Detailed Long-Term Follow-Up of Patients Who Relapsed After the Nordic Mantle Cell Lymphoma Trials: MCL2 and MCL3. HemaSphere *5*, E510. https://doi.org/10.1097/HS9.0000000000000510.
28. Eskelund, C.W., Dahl, C., Hansen, J.W., Westman, M., Kolstad, A., Pedersen, L.B., Montano-Almendras, C.P., Husby, S., Freiburghaus, C., Ek, S., et al. (2017). TP53 mutations identify younger mantle cell lymphoma patients who do not benefit from intensive chemoimmunotherapy. Blood *130*, 1903–1910. https://doi.org/10.1182/BLOOD-2017-04-779736.
29. Delfau-Larue, M.H., Klapper, W., Berger, F., Jardin, F., Briere, J., Salles, G., Casasnovas, O., Feugier, P., Haioun, C., Ribrag, V., et al. (2015). High-dose cytarabine does not overcome the adverse prognostic value of CDKN2A and TP53 deletions in mantle cell lymphoma. Blood *126*, 604–611. https://doi.org/10.1182/blood-2015-02-628792.
30. Yi, S., Yan, Y., Jin, M., Bhattacharya, S., Wang, Y., Wu, Y., Yang, L., Gine, E., Clot, G., Chen, L., et al. (2022). Genomic and transcriptomic profiling reveals distinct molecular subsets associated with outcomes in mantle cell lymphoma. J. Clin. Investig. *132*, e153283. https://doi.org/10.1172/JCI153283.
31. Wang, L., Mo, S., Li, X., He, Y., and Yang, J. (2020). Single-cell RNA-seq reveals the immune escape and drug resistance mechanisms of mantle cell lymphoma. Cancer Biol. Med. *17*, 726–739. https://doi.org/10.20892/j.issn.2095-3941.2020.0073.
32. Tam, C.S., Anderson, M.A., Pott, C., Agarwal, R., Handunnetti, S., Hicks, R.J., Burbury, K., Turner, G., Di Iulio, J., Bressel, M., et al. (2018). Ibrutinib plus Venetoclax for the Treatment of Mantle-Cell Lymphoma. N. Engl. J. Med. *378*, 1211–1223. https://doi.org/10.1056/NEJMoa1715519.
33. Saleh, K., Cheminant, M., Chiron, D., Burroni, B., Ribrag, V., and Sarkozy, C. (2022). Tumor microenvironment and immunotherapy-based approaches in mantle cell lymphoma. Cancers (Basel) *14*, 3229. https://doi.org/10.3390/cancers14133229.
34. Valentin Hansen, S., Høy Hansen, M., Cédile, O., Møller, M.B., Haaber, J., Abildgaard, N., and Guldborg Nyvold, C. (2021). Detailed characterization of the transcriptome of single B cells in mantle cell lymphoma suggesting a potential use for SOX4. Sci. Rep. *11*, 19092. https://doi.org/10.1038/s41598-021-98560-1.
35. Zhang, S., Jiang, V.C., Han, G., Hao, D., Lian, J., Liu, Y., Cai, Q., Zhang, R., McIntosh, J., Wang, R., et al. (2021). Longitudinal single-cell profiling reveals molecular heterogeneity and tumor-immune evolution in refractory mantle cell lymphoma. Nat. Commun. *12*, 2877. https://doi.org/10.1038/s41467-021-22872-z.
36. Jiang, V.C., Hao, D., Jain, P., Li, Y., Cai, Q., Yao, Y., Nie, L., Liu, Y., Jin, J., Wang, W., et al. (2022). TIGIT is the central player in T-cell suppression associated with CAR T-cell relapse in mantle cell lymphoma. Mol. Cancer *21*, 185. https://doi.org/10.1186/s12943-022-01655-0.
37. Domínguez Conde, C., Xu, C., Jarvis, L.B., Rainbow, D.B., Wells, S.B., Gomes, T., Howlett, S.K., Suchanek, O., Polanski, K., King, H.W., et al.

(2022). Cross-tissue immune cell analysis reveals tissue-specific features in humans. Science *376*, eabl5197. https://doi.org/10.1126/SCIENCE.ABL5197.

38. Han, G., Deng, Q., Marques-Piubelli, M.L., Dai, E., Dang, M., Ma, M.C.J., Li, X., Yang, H., Henderson, J., Kudryashova, O., et al. (2022). Follicular Lymphoma Microenvironment Characteristics Associated with Tumor Cell Mutations and MHC Class II Expression. Blood Cancer Discov. *3*, 428–443. https://doi.org/10.1158/2643-3230.bcd-21-0075.
39. Scott, D.W., Abrisqueta, P., Wright, G.W., Slack, G.W., Mottok, A., Villa, D., Jares, P., Rauert-Wunderlich, H., Royo, C., Clot, G., et al. (2017). New molecular assay for the proliferation signature in mantle cell lymphoma applicable to formalin-fixed paraffin-embedded biopsies. J. Clin. Oncol. *35*, 1668–1677. https://doi.org/10.1200/JCO.2016.70.7901.
40. Zheng, L., Qin, S., Si, W., Wang, A., Xing, B., Gao, R., Ren, X., Wang, L., Wu, X., Zhang, J., et al. (2021). Pan-cancer single-cell landscape of tumor-infiltrating T cells. Science *374*, abe6474. https://doi.org/10.1126/science.abe6474.
41. Tang, F., Li, J., Qi, L., Liu, D., Bo, Y., Qin, S., Miao, Y., Yu, K., Hou, W., Li, J., et al. (2023). A pan-cancer single-cell panorama of human natural killer cells. Cell *186*, 4235–4251.e20. https://doi.org/10.1016/j.cell.2023.07.034.
42. Ye, X., Wang, L., Nie, M., Wang, Y., Dong, S., Ren, W., Li, G., Li, Z.M., Wu, K., and Pan-Hammarström, Q. (2022). A single-cell atlas of diffuse large B cell lymphoma. Cell Rep. *39*, 110713. https://doi.org/10.1016/j.celrep.2022.110713.
43. Zhang, Q., He, Y., Luo, N., Patel, S.J., Han, Y., Gao, R., Modak, M., Carotta, S., Haslinger, C., Kind, D., et al. (2019). Landscape and Dynamics of Single Immune Cells in Hepatocellular Carcinoma. Cell *179*, 829–845.e20. https://doi.org/10.1016/J.CELL.2019.10.003.
44. Efremova, M., Vento-Tormo, M., Teichmann, S.A., and Vento-Tormo, R. (2020). CellPhoneDB: inferring cell–cell communication from combined expression of multi-subunit ligand–receptor complexes. Nat. Protoc. *15*, 1484–1506. https://doi.org/10.1038/s41596-020-0292-x.
45. Chen, L., and Flies, D.B. (2013). Molecular mechanisms of T cell co-stimulation and co-inhibition. Nat. Rev. Immunol. *13*, 227–242. https://doi.org/10.1038/nri3405.
46. Haebe, S., Shree, T., Sathe, A., Day, G., Czerwinski, D.K., Grimes, S.M., Lee, H., Binkley, M.S., Long, S.R., Martin, B., et al. (2021). Single-cell analysis can define distinct evolution of tumor sites in follicular lymphoma. Blood *137*, 2869–2880. https://doi.org/10.1182/blood.2020009855.
47. Qiu, X., Mao, Q., Tang, Y., Wang, L., Chawla, R., Pliner, H.A., and Trapnell, C. (2017). Reversed graph embedding resolves complex single-cell trajectories. Nat. Methods *14*, 979–982. https://doi.org/10.1038/nmeth.4402.
48. Ding, L., Ley, T.J., Larson, D.E., Miller, C.A., Koboldt, D.C., Welch, J.S., Ritchey, J.K., Young, M.A., Lamprecht, T., McLellan, M.D., et al. (2012). Clonal evolution in relapsed acute myeloid leukaemia revealed by whole-genome sequencing. Nature *481*, 506–510. https://doi.org/10.1038/nature10738.
49. Gillis, S., and Roth, A. (2020). PyClone-VI: scalable inference of clonal population structures using whole genome data. BMC Bioinf. *21*, 571. https://doi.org/10.1186/S12859-020-03919-2.
50. Dang, H.X., White, B.S., Foltz, S.M., Miller, C.A., Luo, J., Fields, R.C., and Maher, C.A. (2017). ClonEvol: Clonal ordering and visualization in cancer sequencing. Ann. Oncol. *28*, 3076–3082. https://doi.org/10.1093/annonc/mdx517.
51. Walsh, S.H., Thorsélius, M., Johnson, A., Söderberg, O., Jerkeman, M., Björck, E., Eriksson, I., Thunberg, U., Landgren, O., Ehinger, M., et al. (2003). Mutated VH genes and preferential VH3-21 use define new subsets of mantle cell lymphoma. Blood *101*, 4047–4054. https://doi.org/10.1182/BLOOD-2002-11-3479.
52. Ghia, E.M., Jain, S., Widhopf, G.F., 2nd, Rassenti, L.Z., Keating, M.J., Wierda, W.G., Gribben, J.G., Brown, J.R., Rai, K.R., Byrd, J.C., et al. (2008). Use of IGHV3–21 in chronic lymphocytic leukemia is associated with high-risk disease and reflects antigen-driven, post–germinal center leukemogenic selection. Blood *111*, 5101–5108. https://doi.org/10.1182/BLOOD-2007-12-130229.
53. Kostareli, E., Gounari, M., Agathangelidis, A., and Stamatopoulos, K. (2012). Immunoglobulin gene repertoire in chronic lymphocytic leukemia: insight into antigen selection and microenvironmental interactions. Mediterr. J. Hematol. Infect. Dis. *4*, e2012052. https://doi.org/10.4084/MJHID.2012.052.
54. He, X., Goronzy, J.J., Zhong, W., Xie, C., and Weyand, C.M. (1995). VH3-21 B cells escape from a state of tolerance in rheumatoid arthritis and secrete rheumatoid factor. Mol. Med. *1*, 768–780. https://doi.org/10.1007/bf03401891.
55. Elagib, K.E.E., Tengnér, P., Tengnér, T., Jonsson, R., Thompson, K.M., Natvig, J.B., Wahren-Herlenius, M., and Wahren-Herlenius, M. (1999). Immunoglobulin variable genes and epitope recognition of human monoclonal anti-Ro 52-kd in primary Sjögren's syndrome. Arthritis Rheum. *42*, 2471–2481. https://doi.org/10.1002/1529-0131.
56. Williamson, C.T., Kubota, E., Hamill, J.D., Klimowicz, A., Ye, R., Muzik, H., Dean, M., Tu, L., Gilley, D., Magliocco, A.M., et al. (2012). Enhanced cytotoxicity of PARP inhibition in mantle cell lymphoma harbouring mutations in both ATM and p53. EMBO Mol. Med. *4*, 515–527. https://doi.org/10.1002/emmm.201200229.
57. Wei, W., Grünwald, V., and Herrmann, K. (2025). CD70-targeted cancer theranostics: Progress and challenges. Med, 100671. https://doi.org/10.1016/j.medj.2025.100671.
58. Sauer, T., Parikh, K., Sharma, S., Omer, B., Sedloev, D., Chen, Q., Angenendt, L., Schliemann, C., Schmitt, M., Müller-Tidow, C., et al. (2021). CD70-specific CAR T cells have potent activity against acute myeloid leukemia without HSC toxicity. Blood *138*, 318–330. https://doi.org/10.1182/BLOOD.2020008221.
59. Yang, Z.Z., Grote, D.M., Xiu, B., Ziesmer, S.C., Price-Troska, T.L., Hodge, L.S., Yates, D.M., Novak, A.J., and Ansell, S.M. (2014). TGF-β upregulates CD70 expression and induces exhaustion of effector memory T cells in B-cell non-Hodgkin's lymphoma. Leukemia *28*, 1872–1884. https://doi.org/10.1038/leu.2014.84.
60. Yang, Z.Z., Novak, A.J., Ziesmer, S.C., Witzig, T.E., and Ansell, S.M. (2007). CD70+ non-Hodgkin lymphoma B cells induce Foxp3 expression and regulatory function in intratumoral CD4+CD25 T cells. Blood *110*, 2537–2544. https://doi.org/10.1182/blood-2007-03-082578.
61. Tesselaar, K., Arens, R., van Schijndel, G.M.W., Baars, P.A., van der Valk, M.A., Borst, J., van Oers, M.H.J., and van Lier, R.A.W. (2003). Lethal T cell immunodeficiency induced by chronic costimulation via CD27-CD70 interactions. Nat. Immunol. *4*, 49–54. https://doi.org/10.1038/ni869.
62. Claus, C., Riether, C., Schürch, C., Matter, M.S., Hilmenyuk, T., and Ochsenbein, A.F. (2012). CD27 signaling increases the frequency of regulatory T cells and promotes tumor growth. Cancer Res. *72*, 3664–3676. https://doi.org/10.1158/0008-5472.CAN-11-2791.
63. Balsas, P., Veloza, L., Clot, G., Sureda-Gómez, M., Rodríguez, M.L., Masaoutis, C., Frigola, G., Navarro, A., Beà, S., Nadeu, F., et al. (2021). SOX11, CD70, and Treg cells configure the tumor-immune microenvironment of aggressive mantle cell lymphoma. Blood *138*, 2202–2215. https://doi.org/10.1182/blood.2020010527.
64. Riether, C., Schürch, C.M., Bührer, E.D., Hinterbrandner, M., Huguenin, A. L., Hoepner, S., Zlobec, I., Pabst, T., Radpour, R., and Ochsenbein, A.F. (2017). CD70/CD27 signaling promotes blast stemness and is a viable therapeutic target in acute myeloid leukemia. J. Exp. Med. *214*, 359–380. https://doi.org/10.1084/jem.20152008.
65. Flieswasser, T., Van den Eynde, A., Van Audenaerde, J., De Waele, J., Lardon, F., Riether, C., de Haard, H., Smits, E., Pauwels, P., and Jacobs, J. (2022). The CD70-CD27 axis in oncology: the new kids on the block. J. Exp. Clin. Cancer Res. *41*, 12–15. https://doi.org/10.1186/s13046-021-02215-y.
66. Nie, M., Ren, W., Ye, X., Berglund, M., Wang, X., Fjordén, K., Du, L., Giannoula, Y., Lei, D., Su, W., et al. (2022). The dual role of CD70 in B-cell

CellPress
OPEN ACCESS

lymphomagenesis. Clin. Transl. Med. *12*, e1118. https://doi.org/10.1002/ctm2.1118.

67. Sarkozy, C., Wu, S., Takata, K., Aoki, T., Neriah, S.B., Milne, K., Nelson, B., Weng, A., Scott, D., Craig, J.W., et al. (2024). Integrated single cell analysis reveals co-evolution of malignant B cells and the tumor microenvironment in transformed follicular lymphoma. Cancer Cell *42*, 1003–1017.e1006. https://doi.org/10.1016/j.ccell.2024.05.011.
68. Andor, N., Simonds, E.F., Czerwinski, D.K., Chen, J., Grimes, S.M., Wood-Bouwens, C., Zheng, G.X.Y., Kubit, M.A., Greer, S., Weiss, W.A., et al. (2019). Single-cell RNA-Seq of follicular lymphoma reveals malignant B-cell types and coexpression of T-cell immune checkpoints. Blood *133*, 1119–1129. https://doi.org/10.1182/BLOOD-2018-08-862292.
69. Petitprez, F., de Reyniès, A., Keung, E.Z., Chen, T.W.W., Sun, C.M., Calderaro, J., Jeng, Y.M., Hsiao, L.P., Lacroix, L., Bougoüin, A., et al. (2020). B cells are associated with survival and immunotherapy response in sarcoma. Nature *577*, 556–560. https://doi.org/10.1038/s41586-019-1906-8.
70. Helmink, B.A., Reddy, S.M., Gao, J., Zhang, S., Basar, R., Thakur, R., Yizhak, K., Sade-Feldman, M., Blando, J., Han, G., et al. (2020). B cells and tertiary lymphoid structures promote immunotherapy response. Nature *577*, 549–555. https://doi.org/10.1038/S41586-019-1922-8.
71. Budczies, J., Kirchner, M., Kluck, K., Kazdal, D., Glade, J., Allgäuer, M., Kriegsmann, M., Heußel, C.P., Herth, F.J., Winter, H., et al. (2021). A gene expression signature associated with B cells predicts benefit from immune checkpoint blockade in lung adenocarcinoma. OncoImmunology *10*, 1860586. https://doi.org/10.1080/2162402X.2020.1860586.
72. Bankhead, P., Loughrey, M.B., Fernández, J.A., Dombrowski, Y., Mcart, D. G., Dunne, P.D., McQuaid, S., Gray, R.T., Murray, L.J., Coleman, H.G., et al. (2017). QuPath: Open source software for digital pathology image analysis. Sci. Rep. *7*, 16878. https://doi.org/10.1038/s41598-017-17204-5.
73. Miller, C.A., McMichael, J., Dang, H.X., Maher, C.A., Ding, L., Ley, T.J., Mardis, E.R., and Wilson, R.K. (2016). Visualizing tumor evolution with the fishplot package for R. BMC Genom. *17*, 880–883. https://doi.org/10.1186/S12864-016-3195-Z.
74. Thome, J.J.C., Yudanin, N., Ohmura, Y., Kubota, M., Grinshpun, B., Sathaliyawala, T., Kato, T., Lerner, H., Shen, Y., and Farber, D.L. (2014). Spatial map of human T cell compartmentalization and maintenance over decades of life. Cell *159*, 814–828. https://doi.org/10.1016/j.cell.2014.10.026.
75. Hänzelmann, S., Castelo, R., and Guinney, J. (2013). GSVA: Gene set variation analysis for microarray and RNA-Seq data. BMC Bioinf. *14*, 1–15. https://doi.org/10.1186/1471-2105-14-7/FIGURES/7.
76. Liberzon, A., Birger, C., Thorvaldsdóttir, H., Ghandi, M., Mesirov, J.P., and Tamayo, P. (2015). The Molecular Signatures Database (MsigDB) hallmark gene set collection. Cell Syst. *1*, 417–425. https://doi.org/10.1016/j.cels.2015.12.004.
77. Kotliar, D., Veres, A., Nagy, M.A., Tabrizi, S., Hodis, E., Melton, D.A., and Sabeti, P.C. (2019). Identifying gene expression programs of cell-type identity and cellular activity with single-cell RNA-Seq. eLife *8*, e43803. https://doi.org/10.7554/ELIFE.43803.
78. Dimitrov, D., Türei, D., Garrido-Rodriguez, M., Burmedi, P.L., Nagai, J.S., Boys, C., Ramirez Flores, R.O., Kim, H., Szalai, B., Costa, I.G., et al. (2022). Comparison of methods and resources for cell-cell communication inference from single-cell RNA-Seq data. Nat. Commun. *13*, 3224. https://doi.org/10.1038/s41467-022-30755-0.
79. Turei, D., Korcsmaros, T., and Saez-Rodriguez, J. (2016). OmniPath: guidelines and gateway for literature-curated signaling pathway resources. Nat. Methods *13*, 966–967. https://doi.org/10.1038/nmeth.4077.
80. Li, H., and Durbin, R. (2009). Fast and accurate short read alignment with Burrows-Wheeler transform. Bioinformatics *25*, 1754–1760. https://doi.org/10.1093/bioinformatics/btp324.
81. McKenna, A., Hanna, M., Banks, E., Sivachenko, A., Cibulskis, K., Kernytsky, A., Garimella, K., Altshuler, D., Gabriel, S., Daly, M., and Depristo, M. A. (2010). The Genome Analysis Toolkit: A MapReduce framework for analyzing next-generation DNA sequencing data. Genome Res. *20*, 1297–1303. https://doi.org/10.1101/gr.107524.110.
82. Cibulskis, K., McKenna, A., Fennell, T., Banks, E., DePristo, M., and Getz, G. (2011). ContEst: estimating cross-contamination of human samples in next-generation sequencing data. Bioinformatics *27*, 2601–2602. https://doi.org/10.1093/bioinformatics/btr446.
83. Larson, D.E., Harris, C.C., Chen, K., Koboldt, D.C., Abbott, T.E., Dooling, D.J., Ley, T.J., Mardis, E.R., Wilson, R.K., and Ding, L. (2012). SomaticSniper: identification of somatic point mutations in whole genome sequencing data. Bioinformatics *28*, 311–317. https://doi.org/10.1093/BIOINFORMATICS/BTR665.
84. Kim, S., Scheffler, K., Halpern, A.L., Bekritsky, M.A., Noh, E., Källberg, M., Chen, X., Kim, Y., Beyter, D., Krusche, P., and Saunders, C.T. (2018). Strelka2: fast and accurate calling of germline and somatic variants. Nat. Methods *15*, 591–594. https://doi.org/10.1038/s41592-018-0051-x.
85. Fan, Y., Xi, L., Hughes, D.S.T., Zhang, J., Zhang, J., Futreal, P.A., Wheeler, D.A., and Wang, W. (2016). MuSE: accounting for tumor heterogeneity using a sample-specific error model improves sensitivity and specificity in mutation calling from sequencing data. Genome Biol. *17*, 178. https://doi.org/10.1186/s13059-016-1029-6.
86. Narzisi, G., Corvelo, A., Arora, K., Bergmann, E.A., Shah, M., Musunuri, R., Emde, A.K., Robine, N., Vacic, V., and Zody, M.C. (2018). Genome-wide somatic variant calling using localized colored de Bruijn graphs. Commun. Biol. *1*, 20–29. https://doi.org/10.1038/s42003-018-0023-9.
87. Wala, J.A., Bandopadhayay, P., Greenwald, N.F., O'Rourke, R., Sharpe, T., Stewart, C., Schumacher, S., Li, Y., Weischenfeldt, J., Yao, X., et al. (2018). SvABA: genome-wide detection of structural variants and indels by local assembly. Genome Res. *28*, 581–591. https://doi.org/10.1101/GR.221028.117.
88. Wang, K., Li, M., and Hakonarson, H. (2010). ANNOVAR: functional annotation of genetic variants from high-throughput sequencing data. Nucleic Acids Res. *38*, e164. https://doi.org/10.1093/NAR/GKQ603.
89. Itan, Y., Shang, L., Boisson, B., Patin, E., Bolze, A., Moncada-Vélez, M., Scott, E., Ciancanelli, M.J., Lafaille, F.G., Markle, J.G., et al. (2015). The human gene damage index as a gene-level approach to prioritizing exome variants. Proc. Natl. Acad. Sci. USA *112*, 13615–13620. https://doi.org/10.1073/pnas.1518646112.
90. Ren, W., Ye, X., Su, H., Li, W., Liu, D., Pirmoradian, M., Wang, X., Zhang, B., Zhang, Q., Chen, L., et al. (2018). Genetic landscape of hepatitis B virus-associated diffuse large B-cell lymphoma. Blood *131*, 2670–2681. https://doi.org/10.1182/blood-2017-11-817601.
91. Raine, K.M., Van Loo, P., Wedge, D.C., Jones, D., Menzies, A., Butler, A. P., Teague, J.W., Tarpey, P., Nik-Zainal, S., and Campbell, P.J. (2016). ascatNgs: Identifying somatically acquired copy-number alterations from whole-genome sequencing data. Curr. Protoc. Bioinformatics *56*, 15.9.1–15.9.17. https://doi.org/10.1002/cpbi.17.
92. Mermel, C.H., Schumacher, S.E., Hill, B., Meyerson, M.L., Beroukhim, R., and Getz, G. (2011). GISTIC2.0 facilitates sensitive and confident localization of the targets of focal somatic copy-number alteration in human cancers. Genome Biol. *12*, R41. https://doi.org/10.1186/GB-2011-12-4-R41.
93. Chen, X., Schulz-Trieglaff, O., Shaw, R., Barnes, B., Schlesinger, F., Källberg, M., Cox, A.J., Kruglyak, S., and Saunders, C.T. (2016). Manta: Rapid detection of structural variants and indels for germline and cancer sequencing applications. Bioinformatics *32*, 1220–1222. https://doi.org/10.1093/bioinformatics/btv710.
94. Liang, Y., Qiu, K., Liao, B., Zhu, W., Huang, X., Li, L., Chen, X., and Li, K. (2017). Seeksv: An accurate tool for somatic structural variation and virus integration detection. Bioinformatics *33*, 184–191. https://doi.org/10.1093/bioinformatics/btw591.
95. Ritchie, M.E., Phipson, B., Wu, D., Hu, Y., Law, C.W., Shi, W., and Smyth, G.K. (2015). limma powers differential expression analyses for RNA-sequencing and microarray studies. Nucleic Acids Res. *43*, e47. https://doi.org/10.1093/nar/gkv007.

STAR★METHODS

KEY RESOURCES TABLE

REAGENT or RESOURCE	SOURCE	IDENTIFIER
**Biological samples**		
Fresh tissue samples	Karolinska University Hospital	See Table S1 for details
**Critical commercial assays**		
Chromium Next GEM Single-Cell 5′ Kit v2	10× Genomics	Cat#PN-1000263
Chromium Single Cell Human BCR Amplification Kit	10× Genomics	Cat#PN-1000253
DNeasy Tissue and Blood Kit	Qiagen	Cat#69506
EasySep human B-cell enrichment kit	STEMCELL	Cat#19054
PANO 7-plex IHC kit	Panovue	Cat#0004100100
**Antibodies**		
Anti-CD70 [E3Q1A]	Cell Signaling Technology	Cat#CST69209
Anti-CD20 [EP459Y]	Abcam	Cat#ab78237; RRID:AB_1640323
Anti-CCND1 [SP4]	Abcam	Cat#ab16663; RRID:AB_443423
Anti-CD4	ZSGB-BIO	Cat#ZM0418; RRID:AB_2890106
Anti-FOXP3 [BP6166]	BioLynx	Cat#BX50188
Anti-PD1[D4W2J]	Cell Signaling Technology	Cat#CST86163; RRID:AB_2728833
Anti-CD8A [C8/144B]	Cell Signaling Technology	Cat#CST70306; RRID:AB_2799781
Anti-CD27 [M-T271]	BD Biosciences	Cat#555439; RRID:AB_395832
**Experimental models: Cell lines**		
Human: JVM-2	ATCC	CRL-3002; RRID:CVCL_1319
**Deposited data**		
Raw scRNA-seq and WGS data	This paper	https://doi.org/10.48723/kfy8-5r05
H5 files of processed scRNA-seq data	This paper	GEO: GSE303064
Somatic mutations file of processed WGS data	This paper	Zenodo Data: https://doi.org/10.5281/zenodo.16098829
Code for scRNA-seq analysis	This paper	Github: https://github.com/wanhui5867/MCL-scRNAseq/
Fastq files of scRNA-seq data of normal bone marrow samples	EMBL-EBI	EMBL-EBI: E-MTAB-11536
scRNAseq matrics of gene expression and cell annotation of reactive lymph node samples	Han et al.[38]	https://cellxgene.cziscience.com/collections/968834a0-1895-40df-8720-666029b3bbac
**Software and algorithms**		
QuPath 0.5.1	Bankhead et al.[72]	https://qupath.github.io/
Cell Ranger 7.0.0	10× Genomics	https://www.10xgenomics.com/support/software/cell-ranger/
Seurat 4.3.0	Satija Lab	https://satijalab.org/seurat/
inferCNV 1.16.0	Broad Institute	https://github.com/broadinstitute/infercnv
Monocle 2.28.0	Trapnell Lab	https://cole-trapnell-lab.github.io/monocle-release/
LIANA 0.1.13	Saez Lab	https://github.com/saezlab/liana/
R 4.3.0	R Core Team	https://www.r-project.org/
SHazaM 1.1.2	Immcantation Portal	https://shazam.readthedocs.io/
Djvdj 0.1.0	Sheridan R 2023	https://github.com/rnabioco/djvdj/
Fishplot 0.5.1	Miller et al.[73]	https://github.com/chrisamiller/fishplot/
CloneVol 0.99.11	Dang et al.[50]	https://github.com/hdng/clonevol/
ABSOLUTE	Broad Institute	http://broadinstitute.org/software/ABSOLUTE

EXPERIMENTAL MODEL AND STUDY PARTICIPANT DETAILS

Patient samples

A total of 40 tumor samples from 22 MCL patients were retrospectively collected for this study, with pathological diagnoses established according to the WHO classification. Among them, 11 patients had tumor samples suitable for scRNA-seq, scBCR-seq, and WGS. After quality control, high-quality scRNA-seq data were obtained from all patients, matched scBCR-seq data from 10 patients, and matched WGS data from 9 of these 11 patients. For the remaining 11 patients, tumor samples were only analyzed by WGS. For scRNA-seq and scBCR-seq, viable frozen cells from tumor tissues were used. For WGS, either frozen tumor tissues or viable frozen tumor cells were used, along with matched germline (non-tumor) samples obtained from peripheral blood or sorted/enriched non-B cells. Additionally, one BM sample was obtained from a cancer-free donor and used as a control scRNA-seq dataset. Our MCL patients received various treatments, including watchful waiting, ASCT, radiotherapy, CHOP, and R-CHOP-like regimens. The sample metadata, including donor gender, age, health status, treatment and other clinical factors are summarized in Table S1. Informed consent was obtained from all participants, and the study was approved by the regional ethical review board Stockholm.

METHOD DETAILS

Single-cell preparation and fluorescence-activated cell sorting (FACS)

Sample processing protocols were adapted from protocols described previously.[74] Briefly, mononuclear cells were isolated from BM samples by standard density centrifugation using Lymphoprep (Axis-Shield) following the manufacturer's instructions. Intestinal, tonsillar, and LN tissues were processed into single-cell suspensions using mechanical dissociation followed by filtering through 70 μm containers. Single cells were cryopreserved in fetal bovine serum (FBS) supplemented with 10% dimethyl sulfoxide (DMSO) and stored in liquid nitrogen before use.

Cells used for flow cytometry were thawed in RPMI 1640 cell medium (Gibco). Subsequently, cells were incubated with human FcR Blocking Reagent (Miltenyi Biotec) for 10 min and stained with anti-CD19 BV421 and anti-CD235a FITC in FACS buffer (PBS with 2% FBS) for 20 min on ice. Immediately before sorting, the cells were briefly incubated with propidium iodide staining solution to assess and exclude dead cells. For the MCL samples, live $CD235a^{-}$ cells, $CD235a^{-}CD19^{+}$ cells, and $CD235a^{-}CD19^{-}$ cells were sorted in FACS buffer using a BD FACSAria Fusion Sorter (BD Biosciences). Live $CD235a^{-}$ cells were used for single-cell sequencing, while live $CD235a^{-}$ $CD19^{+}$ and $CD235a^{-}$ $CD19^{-}$ cells were used to extract DNA for WGS of tumor and nontumor cells, respectively. For one control BM sample (BM_Benrich), $CD19^{+}$ cells were enriched for scRNA-seq.

scRNA-seq and scBCR-seq library preparation and sequencing

Using the Chromium Next GEM Single-Cell 5′ Reagent Kit v2 (10× Genomics), cells were loaded onto separate lanes of the Next GEM Chromium Controller (10× Genomics) for encapsulation (target recovery of 10,000 cells for each sample). Single-cell gene expression (GEX) and human B-cell V(D)J libraries were constructed following the manufacturer's protocols. Libraries were sequenced on a NovaSeq 6000 (Illumina) platform.

scRNA-seq and scBCR-seq data processing and analysis

The online scRNA-seq data of normal controls

The raw sequencing data for five normal BMs[37] were downloaded from EMBL-EBI: E-MTAB-11536. The expression and annotation metrics of three RLNs[38] were downloaded from the CZ CELLxGENE: https://cellxgene.cziscience.com/collections/968834a0-1895-40df-8720-666029b3bbac.

Raw sequencing data processing, QC, and data filtering

For each sample, the raw sequencing data were preprocessed using Cell Ranger (version 7.0.0) with default parameters, and the filtered gene count matrix and filtered VDJ annotation were used for downstream analysis. Seurat (version 4.3.0) was used to process the filtered gene count matrix and generate QC metrics. Genes detected in fewer than three cells were removed, and cells expressing fewer than 500 genes were removed. To remove potential doublets or multiplets, cells expressing more than 5000 genes or with sequencing counts exceeding 40,000 were excluded from further analysis. In addition, cells exhibiting high percentages of mitochondrial transcripts (>20%) or low percentages of ribosomal transcripts (<5%) were removed.

Data processing, normalization, dimensionality reduction, and unsupervised cell clustering

Good-quality cells from all the samples were merged using Seurat, and the count data were normalized using the SCTransform function with default parameters. Dimension reduction was performed using principal component analysis (PCA). Subsequently, the RunHarmony function was applied to correct batch effects among datasets. The top 30 principal components were further used for clustering at a resolution of 0.6. Clusters were identified by an SNN modularity optimization-based clustering algorithm and visualized by UMAP.

Cell type identification

To obtain a relatively accurate cell type, the annotation of various cell types was performed in three rounds. First, the cluster of all good-quality cells was annotated by the expression of classic markers of major cell types, including B cells (*CD19*, *MS4A1*, and *CD79A*), T cells (*CD3D*, *CD3E*, and *CD3G*), NK cells (*XCL2*, *NKG7*, and *GNLY*), myeloid cells (*CD68*, *CD33*, and *CST3*), and

erythrocytes (*HBQ1*, *HBM*, and *GYPA*). Second, B cells were extracted and used to infer malignant cells and doublets in patient-specific subclustering (details are provided in the "identification of malignant cells" section). Finally, subclustering was performed within each major cell type to identify subclusters and refine the cell subtype annotation. Batch effect correction was performed on tumor microenvironment cells among datasets and among samples.

BCR V(D)J sequence assembly, SHM calculation, clonotype definition and integration with scRNA-seq data

V(D)J sequencing data were obtained using Cell Ranger (v7.0.0) and annotated using Cell Ranger VDJ data (CellRanger ensembl vdj GRCh38 version 5.0.0). The filtered sequences and annotation results were used for downstream BCR analysis. The SHM of the IGHV region was calculated by shazam (v1.1.2). The V(D)J information was imported to matched scRNA-seq data using djvdj (v0.1.0) if the chain was classified by Cell Ranger as productive, full length, and with high confidence annotations and clonotypes with a paired heavy and light chain. A dominant clone in each sample was defined if the clonotype (same V gene, J gene, and CDR3 peptide) was expressed in more than 5% of the cells, while cells with different clonotypes were classified as polyclonal.

Identification of malignant cells

B cells were extracted and reclustered to infer malignant cells in each patient. First, in the patient-specific B-cell cohort, we used high-resolution clustering to further identify doublets of T and B cells by classic markers of B cells (*CD19*, *MS4A1*, and *CD79A*) and T cells (*CD3E*, *CD4*, and *CD8A*). Malignant B cells were primarily distinguished from normal B cells based on the features of clonal BCR, the IGK/IGL ratio, *CCND1* expression, and the distribution of clusters. A cluster with the same BCR clonotype, a consistent IGK/IGL ratio and high *CCND1* expression was inferred to be a malignant cell cluster. For B cells lacking scBCR-seq data, the IGK/IGL ratio was calculated based on the gene expression of IGKC and IGLC1-7 (IGK/IGL ratio = IGKC/(IGKC+max(IGLC)). Subsequently, inferCNV was employed to infer the CNV from the scRNA-seq data, providing further validation for the identification of malignant cells. The CNV score for each cell was calculated based on the number of genes exhibiting CNVs lower than 0.95 or greater than 1.05 across the entire genome or within specific regions.

Inferring the cell cycle stage, developmental trajectory inference, and pathway enrichment

Cell cycle progression was assessed using the CellCycleScoring function of the Seurat R package. To reconstruct the differentiation trajectory of malignant cells in individuals, we applied Monocle2 with default parameters to infer the pseudotemporal ordering of cells. For the pseudotime analysis, we selected the B cells at the earliest disease stage as the root of the trajectory. The GSVA score was calculated to estimate pathway activity based on the hallmark gene sets downloaded from the Molecular Signature Database (MSigDB) via the GSVA package.[75,76]

Identification of expression programs

An NMF-based method was used to determine the expression programs of malignant cells in each sample. First, cNMF was applied to the expression matrix (genes × cells) 100 times for different numbers of programs (2–10) for malignant cells from each sample.[77] Second, the number of programs for each sample was determined by taking the first number where the cophenetic coefficient started decreasing. Third, each program consisted of a combination of all genes with different expression levels (scores), and the function of each program was predicted by using 30 top-scoring genes, as shown in Table S5. Finally, the programs were hierarchically clustered by using the number of shared genes as a similarity metric, and the function of the meta-program was predicted by the overlapping genes.

Quantification of similarity between samples

The similarity index (SI) was used to quantify the similarity between two tumor samples from the same patient as described by Haebe et al.[46] The SI is defined as the sum of the minimal fractional abundance of each transcriptional subcluster across tumor A and tumor B, with values closer to 1 (100%) representing greater similarity. As suggested by Haebe et al., an SI less than 0.35 is considered low, while an SI greater than 0.67 is classified as high.

Cell−cell interactions

We employed LIANA[78] to estimate cell−cell communication by analyzing the most likely ligand−receptor interactions between different cell types. We utilized LIANA with the CellPhoneDB method and curated the OminiPath resource to predict potential ligand−receptor pairs.[44,79] For further analysis, we focused on ligand−receptor pairs with *p* values less than 0.05 that were expressed in more than 10% of the cells and those that have been previously reported to be immune related.[42,43,45]

Whole-genome sequencing (WGS) and somatic alteration analysis

For fresh-frozen tissue samples, genomic DNA from tumors and matched blood samples was extracted separately by using a DNeasy Tissue and Blood Kit (Qiagen). For the single-cell suspension MCL samples, FACS ($CD19^+$/$CD19^-$) or an EasySep human B-cell enrichment kit was used to separate B cells and non-B cells to represent the tumor samples and nontumor (germline) controls, respectively. Genomic DNA from the sorted cells was extracted using the DNeasy Tissue and Blood Kit. Detailed tumor/germline sample information is documented in Table S2. WGS was performed on a HiSeq 2000 or HiSeq X10 platform (Illumina).

Sequencing reads that contained sequencing adapters, more than 10% unknown bases and low-quality bases (>50% bases with a base quality<5) were removed. Processed sequencing reads were aligned to the human reference genome (hg38) using Burrow-Wheeler Aligner software[80] and further curated following the GATK Best Practices workflow, which includes indel realignment, duplicate read marking and base quality score recalibration.[81] The cross-sample contamination was evaluated by ContEst,[82] and the samples with a contamination rate greater than 4% were filtered out.

To detect somatic single-nucleotide variants (SNVs), five somatic mutation callers were used: Lancet v1.0.719, Strelka2 v2.9.220, MuSE v1.021, Mutect2 (GATK4.0.6) and SomaticSniper v1.0.5.03.[81,83–86] Somatic indels were detected by using Strelka2, Lancet, Mutect2, and Svaba v0.2.14.[87] Highly confident variants were identified by at least two variant callers, and the variants were annotated by ANNOVAR.[88] To eliminate possible germline-induced artifacts, we extracted high-quality reads (mapping quality>20 and base quality>20), covered the position of mutation candidates, and calculated the variant allele frequency (VAF) from all sequenced tumor samples and control samples. Somatic variants were excluded if they appeared in more than 30% (3/10) of the other germline samples in our cohort, as they were likely to be sequencing artifacts. To obtain curated primary- and relapse-specific mutations, we first recovered mutations in those paired samples that may have been missed by the detection pipeline, often due to low VAF. Subsequently, for these primary- and relapse-specific mutations, we assessed the total number of high-quality reads to ensure that coverage at each position was sufficient for detecting mutations (total reads>2). The genes with a high gene damage index (GDI>2000) were removed, except for *ATM*, a well-known tumor suppressor.[89] In addition, genes with low expression levels, as indicated by bulk RNA-seq of MCL (15 samples, average fragments per kilobase of transcript per million mapped reads (FPKM)<0.5), were also removed. Finally, genes that were mutated in at least two patients or in one patient but previously reported in other studies[16,18,23,30,90] were shown in the mutational profile plots.

To detect large-scale variations, ascatNgs was used to determine copy number profiles[91,] and CNVs located in immunoglobulin loci were not considered. The GISTIC2.0 algorithm was used to infer significantly amplified (≥2.3 copies) or deleted (≤1.7 copies) genomic regions.[92] Additionally, the translocation of *IGH-CCND1* was detected by either Manta or SeekSV[93,94] and manually validated by integrative genomics viewer software.

To infer the clonal architecture among longitudinal samples, the CCF of each mutation was first estimated using ABSOLUTE, which was then utilized to infer clonal clusters using PyClone-VI. The clonal architecture was subsequently inferred by ClonEvol v0.99.11[50] and visualized by the fishplot v0.5.1[73] R package.

Bulk transcriptomic sequencing (RNA-seq) and analysis

Transcriptome sequencing was performed on RNA extracted from MCL bulk tumors using TRIzol reagent (Invitrogen). The libraries were prepared at Macrogen Europe and sequenced on a HiSeq 2000 platform. Sequencing reads were aligned to the reference human genome and transcriptome hg38 with HISAT2. FPKM was used to determine gene expression levels, and log2-transformed FPKM values were normalized to remove batch effects using the limma R package.[95]

Immunohistochemical (IHC) and multiplex immunofluorescence (mIF) staining

Formalin-embedded paraffin-embedded (FFPE) MCL tissues and one multitissue microarray were analyzed for protein expression by IHC and mIF staining of 4 μm thick FFPE tissues.

IHC staining and slide scanning were performed at the FoUU Clinical Pathology Service at Uppsala University Hospital as previously described.[66] The sections were subjected to heat-induced antigen retrieval for 20 min at 97°C in Tris EDTA buffer (Agilent). The samples were incubated with primary antibodies specific for human CD70 (MAB2738, clone 301731, 1:100 dilution, R&D Systems) in a DAKO autostainer Link 48 instrument via an Envision Flex detection kit (DAKO) according to the manufacturer's instructions. Image acquisition was performed on a NanoZoomer S60 (Hamamatsu Photonics K.K. Tokyo).

FFPE sections were analyzed using mIF staining to assess the expression of key tumor and immune markers and their colocalization. Two separate mIF panels were used: panel 1: CD20, CCND1, CD70, CD27, CD4, and FOXP3; panel 2: CD20, CCND1, CD70, CD27, CD8, and PD-1. For each panel, tissue sections were deparaffinized, rehydrated, and subjected to antigen retrieval. Primary antibodies specific to the corresponding panel markers were applied, followed by horseradish peroxidase-conjugated secondary antibody incubation and tyramide signal amplification. The slides were heated in a microwave after each secondary antibody incubation. The sections were also stained with 4′-6′-diamidino-2-phenylindole (DAPI, Sigma−Aldrich) to label the nuclei. Staining was performed using a PANO 7-plex IHC kit (Panovue). The stained sections were scanned via an Olympus VS200 slide scanner in conjunction with an Olympus UPLXAP 20× objective lens. Autofluorescence was removed using OlyVIA software, and image analysis was performed using QuPath software.[72]

CD70 knockout in the JVM2 cell line and MCL cell proliferation assay

Three gRNAs targeting CD70 genomic loci were designed within a 100 bp frame to target exon 3 (sgRNA1-AGCCCGCAGGACG CACCCAT, sgRNA2-AGCGCTGGATGCACACCACG, and sgRNA3-CGCCGCGGCGAtGCCGGAGG) obtained at 60 μM and mixed with 20 μM recombinant *Streptococcus pyogenes* Cas9 (Synthego). RNP formation was performed at room temperature for at least 15 min. RNPs were mixed with 5×10^5 cells/strip JVM2 cells for the nucleofection experiment. Nucleofection was performed in a volume of 20 μL per reaction using Amaxa V buffer. Following the addition of all suspensions to 16-well Nucleocuvette Strips (Lonza), electroporation was performed using the Lonza 4D Nucleofector Core Unit with the DN-100 program. After 10 min, 80 μL of prewarmed culture medium was added to each well. After 3 days, CD70 protein expression was assessed by flow post-electroporation to evaluate knockout efficiency. Transfected cells were stained with L/D FVS575V (BD Biosciences) and anti-CD70 (clone 113-16, BioLegend), followed by sorting into $CD70^-$ populations (knockout) using a BD FACSAria Fusion cell sorter. The proliferation was assessed using the CellTrace Violet proliferation assay (Thermo Fisher). Briefly, the cells were labeled according to the

manufacturer's protocol, cultured for three days, and subsequently harvested for flow cytometry analysis to evaluate cell division. Data acquisition was performed on a Sony ID7000 spectral cell analyzer, and the data were analyzed using FlowJo software.

QUANTIFICATION AND STATISTICAL ANALYSIS

Quantification and statistical analyses were performed using R v4.3.0. The Mann–Whitney U test (two-tailed) was used for comparisons between independent groups, and the Wilcoxon signed-rank test (two-tailed) was used for paired samples. Differences in OS between groups were assessed using the log rank test. For DEG analysis, *p* values were calculated using the Wilcoxon rank-sum test and were adjusted using the Benjamini-Hochberg false discovery rate correction and reported as adj-p values. For inferred ligand–receptor interactions, *p* values were calculated by CellPhoneDB using permutation testing (1000 permutations) to assess statistical significance. Statistical significance was defined as $^{*}p < 0.05$, $^{**}p < 0.01$, $^{***}p < 0.005$ and $^{****}p < 0.001$.

Cell Reports Medicine, Volume *6*

Supplemental information

Tumor evolution and immune microenvironment dynamics in primary and relapsed mantle cell lymphoma

Hui Wan, Weicheng Ren, Mingyu Yang, Man Nie, Agata M. Wasik, Likun Du, Leire de Campos-Mata, Rui Sun, Zhiliang Bai, Archibald Enninful, Yating Wang, Mattias Berglund, Rose-Marie Amini, Xiaobo Li, Chunli Yang, Xiaofei Ye, Zhi-Zhang Yang, Stephen M. Ansell, Dongbing Liu, Mirjam van der Burg, Rong Fan, Kui Wu, Birgitta Sander, and Qiang Pan-Hammarström

SUPPLEMENTARY FIGURES

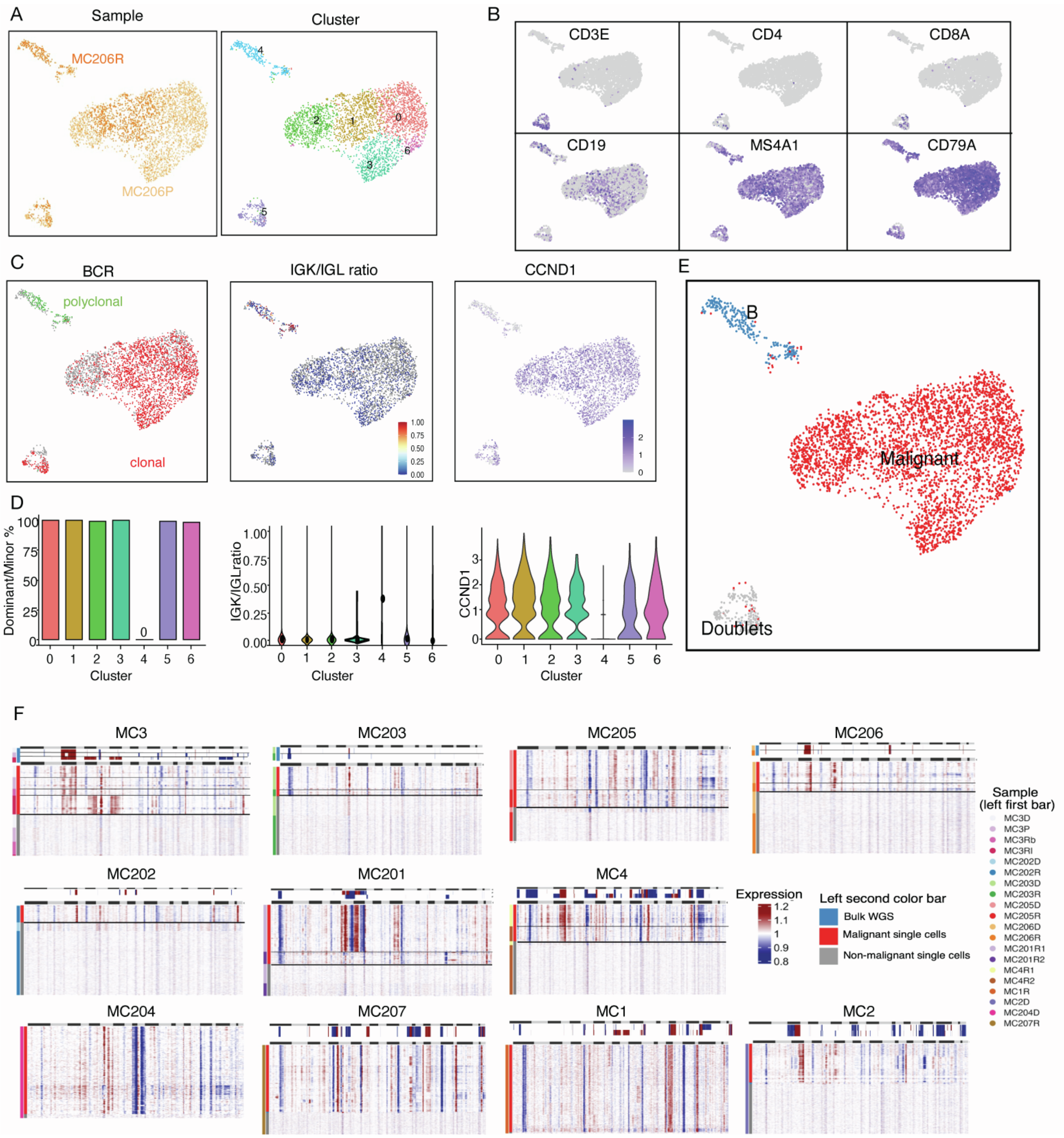

Figure S1. Inferring malignant cells. Related to Figure 1.
Inferring malignant cells using the tumors derived from patient MC206 as an example (A-E). **(A)** UMAP of B cells. Each cell is colored according to the sample origin and clustering number. **(B)** Identification of doublets by checking the gene expression of cell lineage markers. **(C)** UMAP of B cells. Each cell was colored based on the IGK/IGL ratio and clonal BCR. **(D)** Proportion of clonal BCRs and the IGK/IGL ratio for each cell cluster. Each violin plot shows the distribution of IGK/IGL ratio (width represents density), with a central dot indicating the median. **(E)** UMAP of inferred malignant cells and normal B cells. **(F)** Chromosomal landscape CNVs for all MCL samples. The inferred single-cell CNVs are shown with single cells (rows) and chromosomal regions (columns) for each patient. The color in the heatmap represents CNV gain (red) or loss (blue). The cells were grouped into malignant cells and nonmalignant cells, followed by sample origins, which are color-coded on the left. The WGS CNV for matched samples is shown at the top.

We first reclustered all B cells from the two samples derived from this patient and identified seven subclusters (C0-6) (panel A). One subcluster (C5) expressed canonical markers of both B cells and other cell types and was defined as a doublet cluster; therefore, it was excluded from the following analysis (panel B). On the basis of the known features of MCL tumor cells, we annotated five subclusters as malignant cells (C0-3, C6) and one subcluster as nonmalignant cells (C4) (panels C-E). As expected, copy number variants (CNVs), inferred from scRNA-seq data, displayed distinct patterns in malignant cells compared with nonmalignant cells (panel F). Importantly, these CNVs derived from the scRNA-seq data were highly consistent with the CNVs detected from the matched bulk WGS data, confirming the effectiveness of our strategy in identifying malignant B cells.

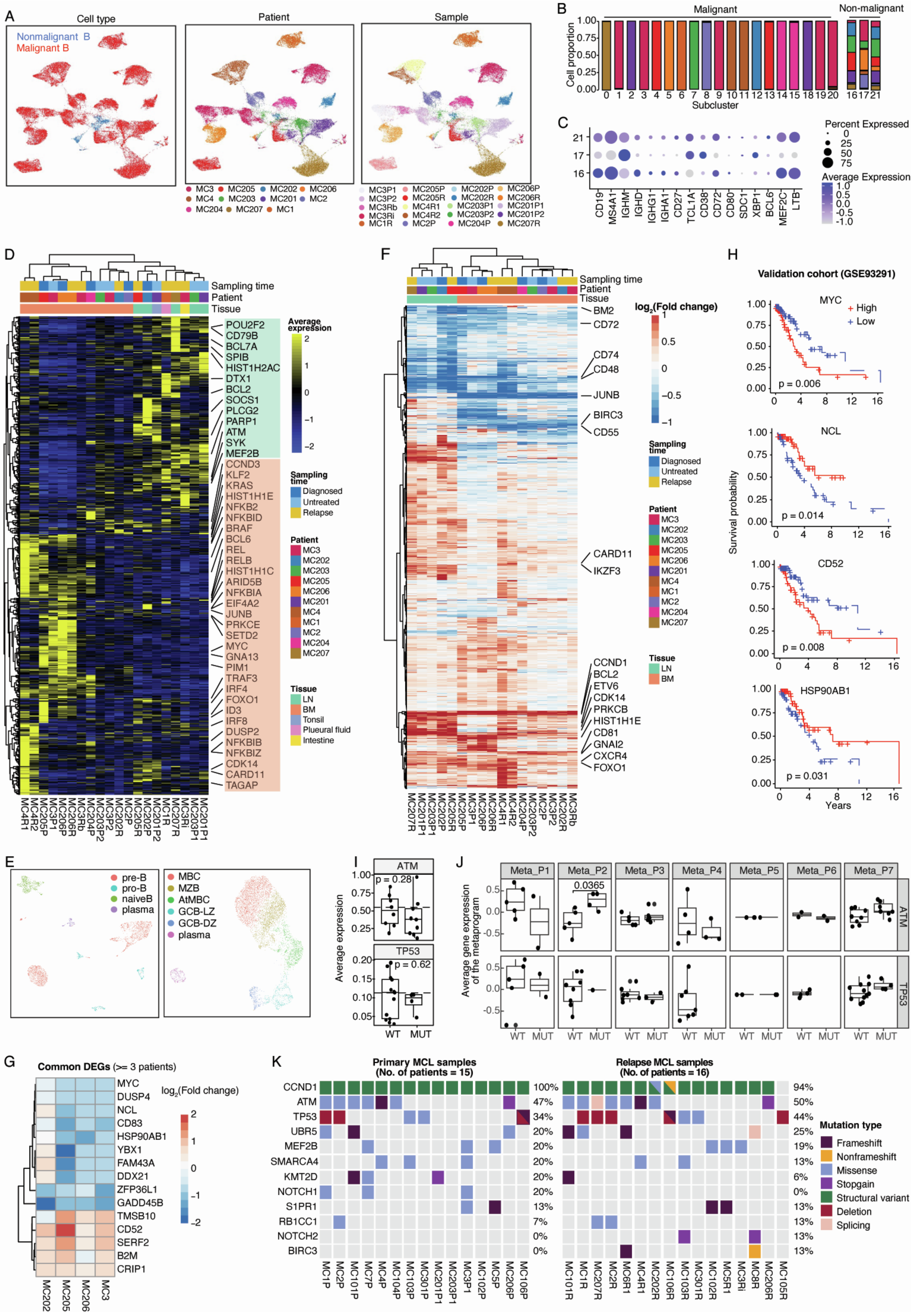
A
Cell type
Nonmalignant B
Malignant B
Patient
MC3 MC205 MC202 MC206
MC4 MC203 MC201 MC2
MC204 MC207 MC1
Sample
MC3P1 MC205P MC202P MC206P
MC3P2 MC205R MC202R MC206R
MC3Rb MC4R1 MC203P1 MC201P1
MC3Ri MC4R2 MC203P2 MC201P2
MC1R MC2P MC204P MC207R
B
Malignant
Non-malignant
Cell proportion
Subcluster
C
Percent Expressed
Average Expression
CD19 MS4A1 IGHM IGHD IGHG1 IGHA1 CD27 TCL1A CD38 CD72 CD80 SDC1 XBP1 BCL6 MEF2C LTB
D
Sampling time
Patient
Tissue
Average expression
POU2F2 CD79B BCL7A SPIB HIST1H2AC DTX1 BCL2 SOCS1 PLCG2 PARP1 ATM SYK MEF2B CCND3 KLF2 KRAS HIST1H1E NFKB2 NFKBID BRAF BCL6 REL RELB HIST1H1C ARID5B NFKBIA EIF4A2 JUNB PRKCE SETD2 MYC GNA13 PIM1 TRAF3 IRF4 FOXO1 ID3 IRF8 DUSP2 NFKBIB NFKBIZ CDK14 CARD11 TAGAP
Diagnosed
Untreated
Relapse
LN
BM
Tonsil
Plueural fluid
Intestine
E
pre-B
pro-B
naiveB
plasma
MBC
MZB
AtMBC
GCB-LZ
GCB-DZ
plasma
F
log2(Fold change)
BM2 CD72 CD74 CD48 JUNB BIRC3 CD55 CARD11 IKZF3 CCND1 BCL2 ETV6 CDK14 PRKCB HIST1H1E CD81 GNAI2 CXCR4 FOXO1
G
Common DEGs (>= 3 patients)
MYC DUSP4 NCL CD83 HSP90AB1 YBX1 FAM43A DDX21 ZFP36L1 GADD45B TMSB10 CD52 SERF2 B2M CRIP1
MC202 MC205 MC206 MC3
H
Validation cohort (GSE93291)
MYC
High
Low
p = 0.006
NCL
p = 0.014
CD52
p = 0.008
HSP90AB1
p = 0.031
Survival probability
Years
I
ATM
p = 0.28
TP53
p = 0.62
Average expression
WT MUT
J
Meta_P1 Meta_P2 Meta_P3 Meta_P4 Meta_P5 Meta_P6 Meta_P7
0.0365
Average gene expression of the metaprogram
ATM
TP53
K
Primary MCL samples (No. of patients = 15)
Relapse MCL samples (No. of patients = 16)
CCND1 ATM TP53 UBR5 MEF2B SMARCA4 KMT2D NOTCH1 S1PR1 RB1CC1 NOTCH2 BIRC3
100% 47% 34% 20% 20% 20% 20% 20% 13% 7% 0% 0%
94% 50% 44% 25% 19% 13% 6% 0% 13% 13% 13% 13%
Mutation type
Frameshift
Nonframeshift
Missense
Stopgain
Structural variant
Deletion
Splicing
MC1P MC2P MC101P MC7P MC4P MC104P MC103P MC301P MC201P1 MC203P1 MC3P1 MC102P MC5P MC206P MC106P
MC101R MC1R MC207R MC2R MC6R1 MC4R1 MC202R MC106R MC103R MC301R MC102R MC5R1 MC3Ri MC8R MC206R MC105R

Figure S2. B cells in MCL patients. Related to Figure 2.
(**A**) UMAP of B cells after removing the human leukocyte antigen and immunoglobulin genes, colored according to the malignant type (left), patient (middle), and sample (right). (**B**) Patient origin of each subcluster. Colors indicate individual patients, consistent with the legend in the middle panel of A. (**C**) The average expression of B-cell markers in nonmalignant B subclusters. **(D)** Highly variable genes (HGVs) across tumors with unsupervised clustering of samples. FindAllMarkers was used to identify HVGs with the following criteria: minimal cell percentage > 0.1, fold change > 1.5 and p.adjust < 0.05. Each column represents a sample, and all the samples were clustered. Each row represents a gene, and the genes related to lymphomagenesis are labeled and colored according to enriched tissues (pink represents bone marrow, green represents non-bone marrow). **(E)** Identification of B-cell subtypes in normal bone marrow and reactive lymph node samples. (**F**) Differentially expressed genes (DEGs) between malignant B cells and normal B cells from matched tissues. Given that MCL tumor cells are thought to originate from naïve B cells or memory B cells, we performed tissue-specific comparisons: malignant cells in BM samples were analyzed against normal BM-derived naïve B cells, while LN tumor cells were compared with normal B cells from RLN (germinal center B cell and plasma cells were excluded). Red indicates upregulated genes in malignant cells, and blue indicates downregulated genes in malignant cells (fold change > 1.5 and p.adjust < 0.05). The common upregulated DEGs and downregulated genes are labeled. **(G)** DEGs were identified between paired primary and relapse samples from individual patients using pseudobulk RNA-seq data. **(H)** Kaplan–Meier survival analysis of DEGs in an independent MCL validation cohort. The p value was calculated from the Log-rank test. (**I**) Comparison of gene expression between wild-type (WT) and mutant (MUT) high-frequency mutated genes in pseudobulk MCL samples. The dashed line represents the average expression of nonmalignant B cells in MCL samples. The p value was calculated from the Mann–Whitney U test. (**J**) Association between gene alterations and meta-program expression in MCL. The p value was calculated from the Mann–Whitney U test. (**K)** Genes with recurrent structural variants or mutations in primary or relapsed MCL tumors. We compared the mutational frequencies between the primary and relapse groups by including initial samples taken from primary and relapse patients. The genes mutated in at least two patients with primary MCL or with relapsed MCL are displayed. The percentage was the frequency of nonsilent mutations in the primary or relapse patient groups. Structural variants in *CCND1* represent *IGH-CCND1* translocations.

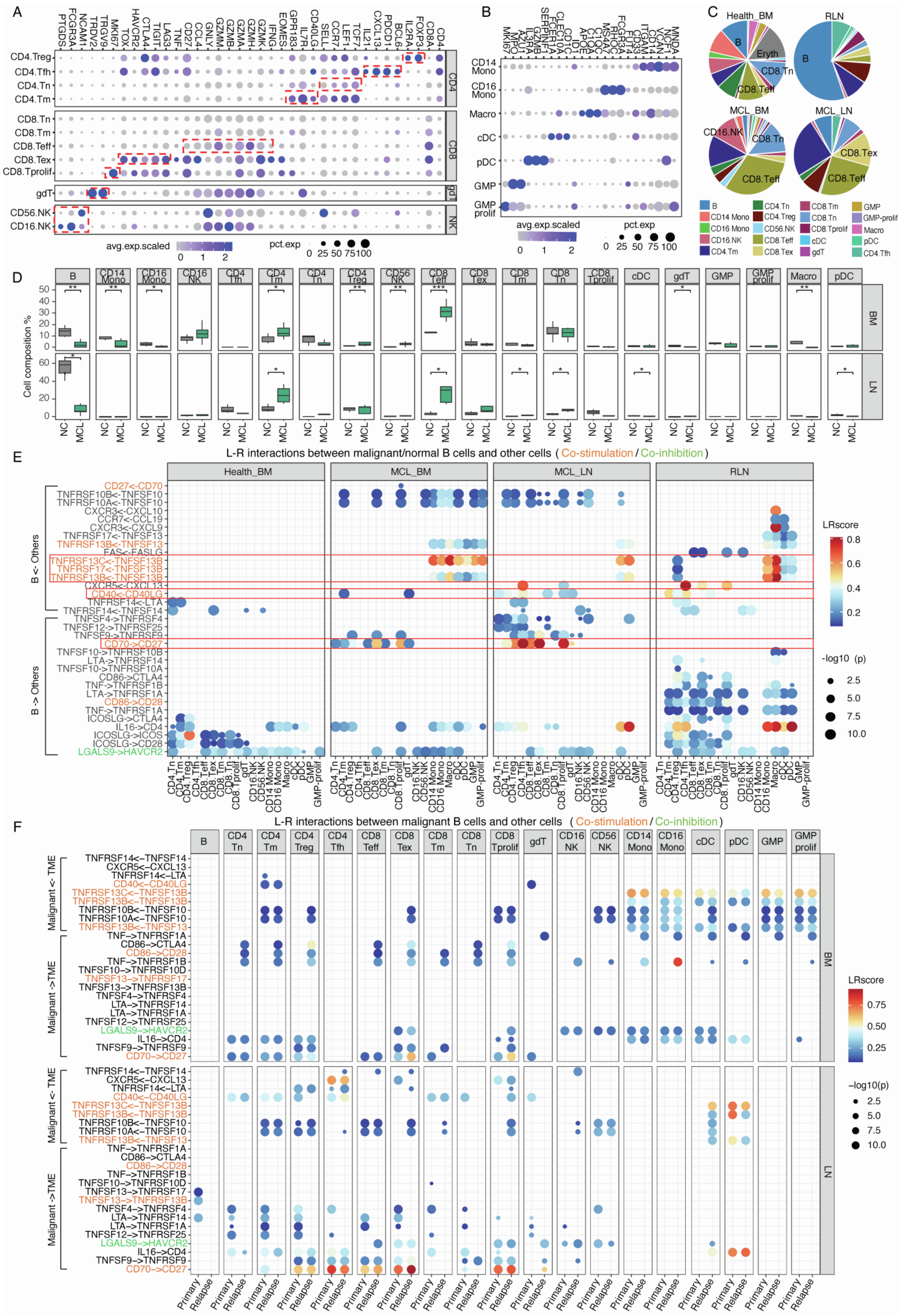

Figure S3. The tumor microenvironment in MCLs. Related to Figure 3.

(A) Dot plot showing marker gene expression across defined T and NK cell clusters. **(B)** Dot plot showing marker gene expression across defined myeloid cell clusters. **(C)** Comparison of the cell population in normal controls and the TME composition in MCLs. Cell populations exceeding 10% of the total composition are labeled. **(D)** Comparison of the relative abundance of each cell subtype between normal controls and MCLs. The p value was calculated from the Mann–Whitney U test. **(E)** Interactions between malignant cells and TMEs in MCL tumors and interactions between B cells and other cells in normal controls. **(F)** Interactions between malignant cells and TME cells at different sampling times in different tumor tissues. One bone marrow control enriched with B cells was excluded from the analysis of cell composition.

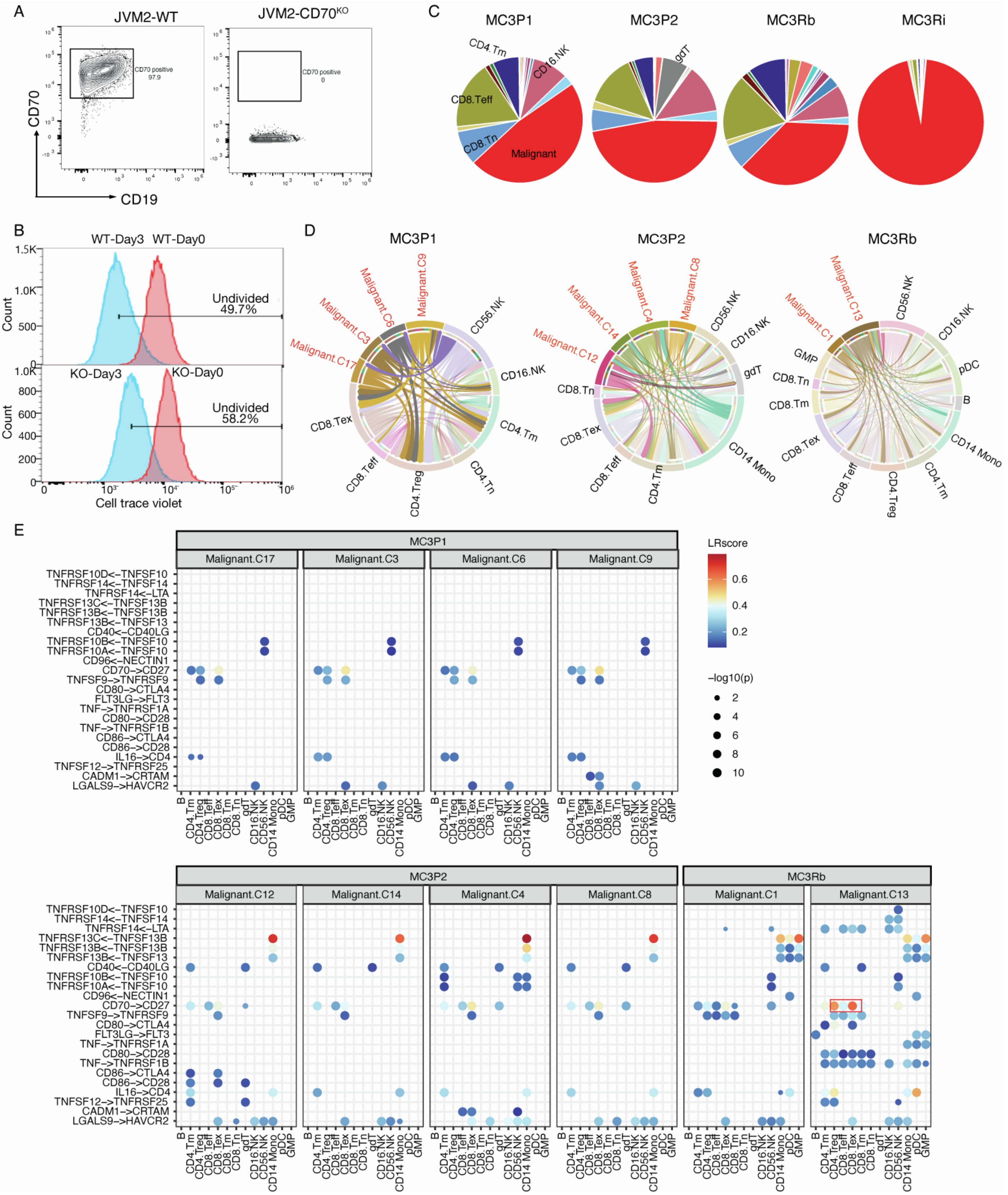

Figure S4. Impact of CD70 expression on MCL proliferation and increased CD70-CD27 interaction between MCL and TME cells in the relapsed tumor of patient MC3. Related to Figure 3 and Figure 6.

(A) Flow cytometry plots of CD70 expression in JVM2 wild-type (JVM2-WT) cells and JVM2-CD70^{KO} cells. (**B**) Proliferation of JVM2-WT and JVM2-CD70^{KO} cells after 3 days in culture. Cell proliferation was assessed using a CellTrace Violet fluorescent dye-based assay, with day 0 undivided cells serving as the baseline control. **(C)** Pie chart showing the cell type composition of each tumor sample. **(D)** Chord diagram showing the crosstalk between cells in each sample. The link width reflects the frequency of interaction between communicating cell types. Only cell subtypes with a cell proportion exceeding 1% are shown. **(E)** Interactions between malignant subclusters and infiltrating immune cells.

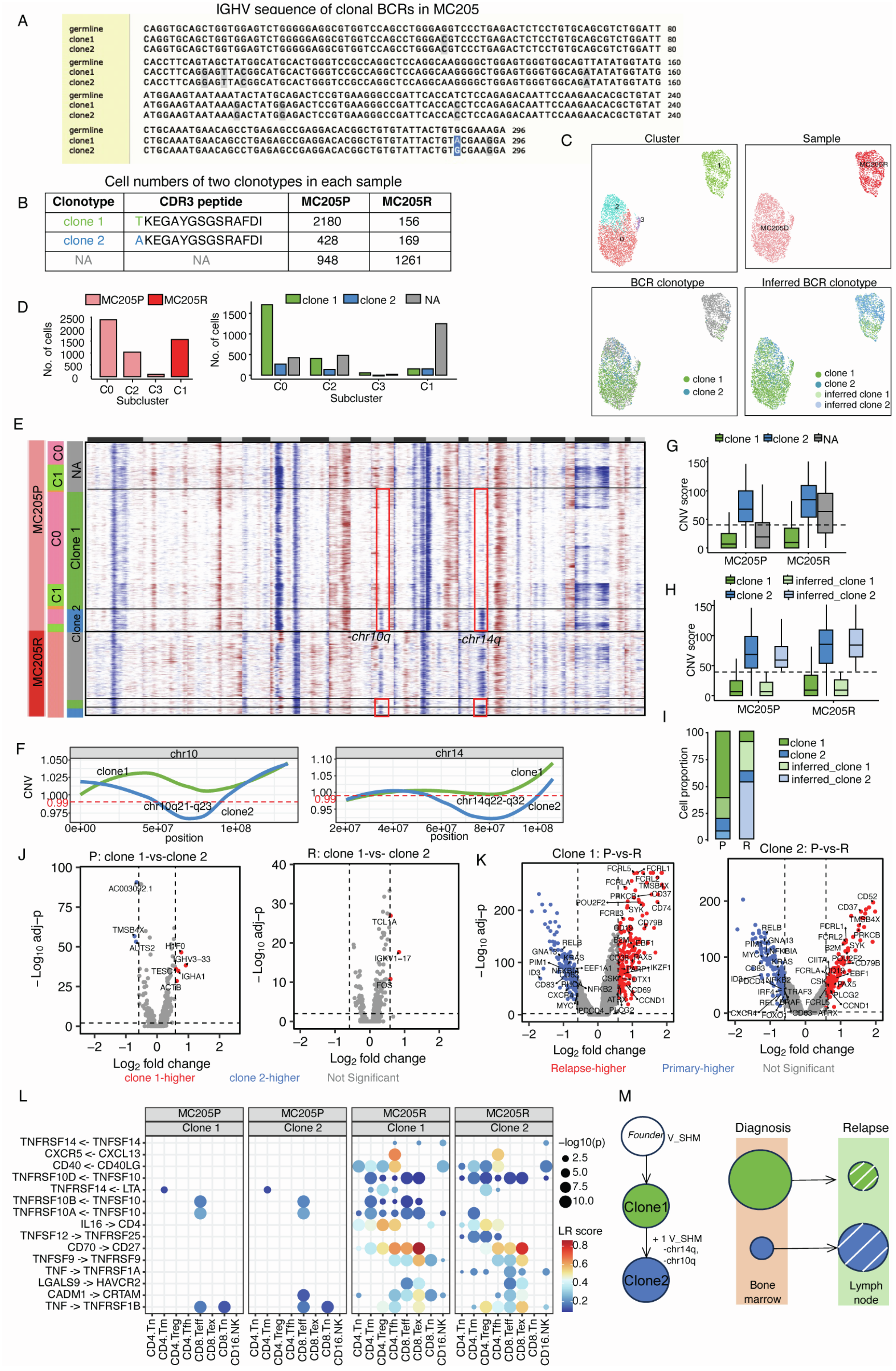

A
IGHV sequence of clonal BCRs in MC205
B
Cell numbers of two clonotypes in each sample
Clonotype | CDR3 peptide | MC205P | MC205R
clone 1 | TKEGAYGSGSRAFDI | 2180 | 156
clone 2 | AKEGAYGSGSRAFDI | 428 | 169
NA | NA | 948 | 1261
C
Cluster
Sample
BCR clonotype
Inferred BCR clonotype
D
MC205P MC205R
No. of cells
Subcluster
clone 1 clone 2 NA
E
MC205P
MC205R
Clone 1
Clone 2
-chr10q
-chr14q
F
chr10
chr14
chr10q21-q23
chr14q22-q32
CNV
position
G
CNV score
H
clone 1 inferred_clone 1
clone 2 inferred_clone 2
I
Cell proportion
J
P: clone 1-vs-clone 2
R: clone 1-vs- clone 2
Log2 fold change
−Log10 adj−p
clone 1-higher
clone 2-higher
Not Significant
K
Clone 1: P-vs-R
Clone 2: P-vs-R
Relapse-higher
Primary-higher
Not Significant
L
−log10(p)
LR score
M
Founder
V_SHM
Clone1
+ 1 V_SHM
-chr14q,
-chr10q
Clone2
Diagnosis
Relapse
Bone marrow
Lymph node

Figure S5. Intratumor heterogeneity and tumor evolution from primary to relapsed tumors in MC205. Related to Figure 5.

(**A**) Alignment of IGHV nucleotide sequences identified from the clonal BCR. (**B**) The number of BCR clonotype cells in each sample. (**C**) UMAPs of malignant cells colored according to transcriptional subclusters, samples, detected BCR clonotypes and inferred BCR clonotypes. (**D**) The cell number (left) and the composition of subclones (right) in each transcriptional subcluster. (**E**) The inferred CNV of malignant cells. The cell identities (samples, subclusters, and subclones) are annotated on the left. (**F**) The CNV changed along chr10 and chr14 in the two clones. CNV=0.99 was used to identify the deletion region in clone 2. (**G**) CNV score of the deletion region in each tumor clone. A CNV score = 40 was used as a threshold to separate clone 1 and clone 2. (**H**) CNV score of the deletion region after inferring the clone type of cells without BCR detection. (**I**) The composition of subclones (right) in each sample. (**J**) Volcano plot of DEGs from clone 1 versus clone 2 in the primary (left) and relapse (right) samples. (**K**) Volcano plot of DEGs between primary and relapse samples in clone 1 (left) and clone 2 (right). (**L**) L-R interaction between malignant subclones and TME cells. (**M**) The inferred clone evolution from diagnosis to relapse. Shading indicates transcriptional changes.

In patient MC205 (treated with ASCT), approximately 73% and 20% of malignant cells were successfully sequenced with BCR clonotypes in primary and relapse samples, respectively. Two BCR clonotypes (clone 1 and clone 2) were detected, distinguished by one mutation in the CDR3 region that generated peptide changes (panel A-B). At the transcriptional level, four distinct subclusters were identified: three (C0, C2, and C3) exclusive to the primary tumor (from the bone marrow) and one (C1) solely present in the relapsed tumor (from the lymph node) (panel C-D). Both BCR clonotypes were found across all subclusters (panel D, right). Interestingly, clone 2 consistently showed deletions of chr14q22-q32 and chr10q21-q23 in both the primary and relapse samples (panels E-F), suggesting a distinct feature of this clone. To further distinguish clone 2 from clone 1, the CNV score of the two deletion regions in each cell was calculated: 86% of clone 1 had a low CNV score (<40), whereas 81% of clone 2 exhibited a higher CNV score (>40) (panel G). Using this approach, we inferred the clonotypes of malignant cells lacking BCR information (panel H), revealing that clone 1 was predominant in the primary tumor, while the proportion of clone 2 increased from 20% in the primary sample to 64% in the relapse sample (panel I). However, these two clones exhibited relatively similar expression patterns, with only minor differences, including decreased expression of BCR (*IGHV3-33*, *IGKV1-17*), *TCL1A*, and *FOS* in clone 2 in both primary and relapsed samples (panel J). We then compared the transcriptional changes in clones 1 and 2 between the primary and relapsed tumors. In the relapsed tumors, both clone 1 and clone 2 exhibited increased expression of genes involved in BCR signaling (*CD19, CD69, CD79B, SYK, FCRL1, FCRL2*), the IFN-γ response (*SP110*, *CD74*), and antigen presentation (*HLA-DQA1, B2M, CIITA*) and downregulated expression of genes involved in the NF-κB (*RELB, NFKB2*) and MYC (*MYC*) signaling pathways, with clone 2 additionally showing downregulation of G2M checkpoint genes (*DMD, HIF1A, NOLC1, YTHDC1, SMAD3*) (panel 6K). Similarly, cell–cell crosstalk analysis revealed greater differences between primary and relapse samples than between clone 1 and clone 2 samples (panel L). Notably, malignant cells in the relapse samples strongly interacted with CD8.Tex cells via CD70–CD27, which might promote T-cell exhaustion. In summary, MC205 tumors displayed two subclones with different genetic alterations but without significant transcriptional changes. However, both subclones exhibited convergent changes in transcriptional activity and interactions with the TME from diagnosis to relapse, indicating that tumor cells may migrate into a favorable TME, altering their cell state and leading to disease relapse (panel M).

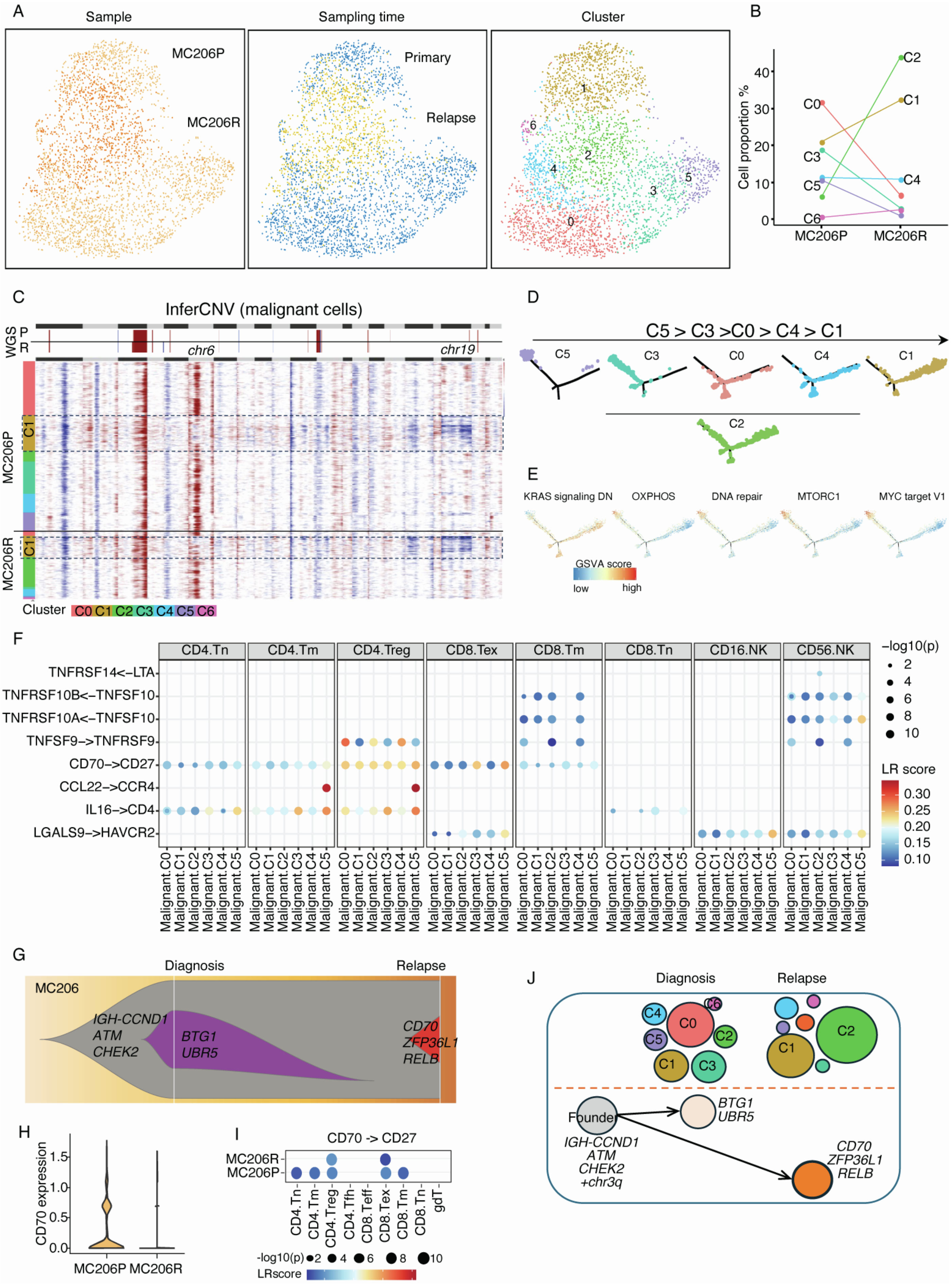

Figure S6. Tumor evolution from diagnosis to relapse in MC206. Related to Figure 5.
(**A**) UMAP of malignant cells colored according to samples, sampling times, and transcriptional subclusters. (**B**) Changes in the malignant subcluster composition between primary and relapsed tumors. (**C**) Large-scale CNVs inferred from WGS (top) and

scRNA-seq (middle) data. Red indicates copy number gain, and blue indicates copy number loss. The root was inferred by starting with primary cells. (**D**) Developmental trajectory representation of malignant cell subclusters inferred by Monocle2. (**E**) The trajectories of malignant cells are colored according to the GSVA score of hallmark pathways. (**F**) L–R interactions between malignant subcluster cells and TME cells. (**G**) Fish plot showing patterns of tumor clonal evolution inferred based on somatic mutations according to WGS data. The representative mutated genes are labeled. (**H**) *CD70* expression in primary and relapse samples. (**I**) CD70–CD27 interaction between malignant cells and T cells in primary and relapse samples. (**J**) The inferred clonal evolution from diagnosis to relapse. The top panel illustrates the changes in the transcriptional subclusters, while the bottom panel shows the clonal evolution based on genetic alterations.

MC206 (treated with R-chemotherapy) presented only one unique BCR clonotype and showed similar gene expression patterns between primary and relapsed tumors. This was also supported by a high SI and non-sample-specific cluster (panel A). Seven transcriptional subclusters (C0-C6) were, however, identified from both samples, with an increased prevalence of C1 and C2 in the relapse sample (panel B). Subcluster C1 displayed additional chr19 deletions and no chr6p amplifications, which is distinct from the other six subclusters (panel C). Trajectory analysis revealed subclusters aligning along a path, starting from C5 and sequentially passing through C3, C0, and C4 and ending at C1 (panel D). Additionally, we observed increased *KRAS* signaling in relapsed tumors and decreased activity of the MYC-targeted, mTORC1, OXPHOS, and DNA repair signaling pathways (panel E). Most subclusters exhibited similar cell crosstalk with TME cells, with C5 showing additional interactions with CD4.Treg and CD4.Tm cells via the CCL22–CCR4 complex (panel F). Clonal evolution analysis based on DNA mutations revealed a founding clone with *IGH-CCND1* translocation, and mutations in *ATM* and *CHEK2*, along with a therapy-eradicated subclone carrying *BTG1* and *UBR5* mutations in the primary tumor, and a subclone with *CD70*, *ZFP36L1*, and *RELB* mutations emerged and expanded in the relapsed tumor (panel G). The relapse sample harbored two small insertions in the coding region of *CD70*, which might contribute to decreased CD70 expression and a reduction in the CD70-CD27 interaction in the relapse sample (panel H-I). Combining genetic and transcriptional data suggested that MC206 tumors carried *IGH-CCND1* translocation and mutations in *ATM* and *CHEK2* and developed into different tumor clones with various mutations (*BTG1* and *UBR5*) and/or transcriptional activities. After treatment, some residual tumor cells acquired additional mutations (*CD70, RELB* and *ZFP36L1*), leading to disease relapse (panel J).

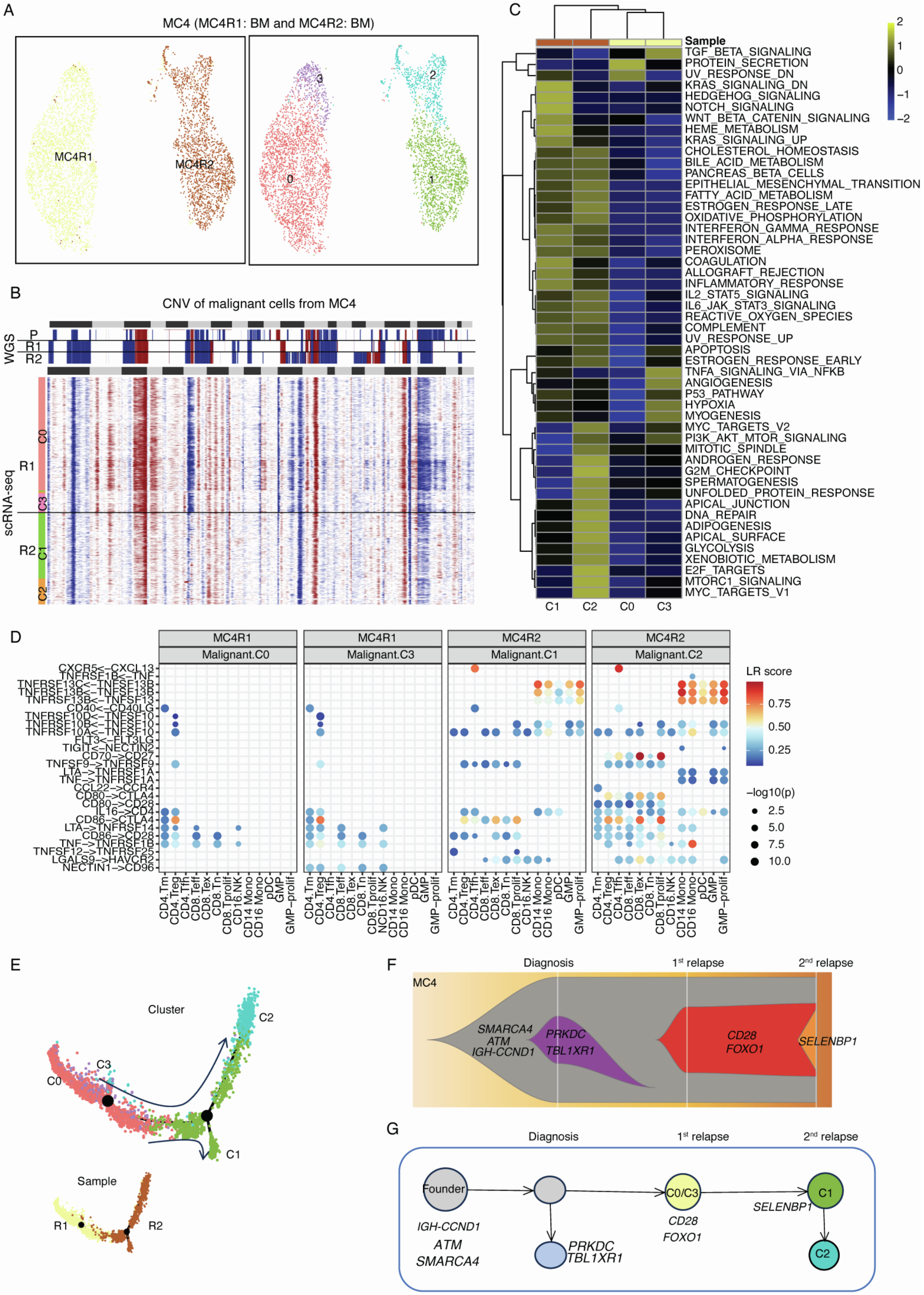

Figure S7. Tumor evolution from the 1st relapse to the 2nd relapse in MC4. Related to Figure 5.

(**A**) UMAP of malignant cells colored according to samples and transcriptional subclusters. (**B**) The inferred CNVs from WGS (top) and scRNA-seq (middle) data. Red indicates copy number gain, and blue indicates copy number loss. (**C**) GSVA of hallmark pathways in each transcriptional subcluster. (**D**) L–R interactions between malignant subcluster cells and TME cells. (**E**) Trajectories of malignant cells, which are colored according to subclusters and samples. The root was inferred from the 1st relapse tumor cells. (**F**) Fish plot showing patterns of tumor clonal evolution inferred based on somatic mutations by WGS data. The representative mutated genes are labeled. (**G**) Inferred clonal evolution in MC4.

In addition to primary-relapse pairs, we analyzed two relapse samples from MC4: one ten years after initial ASCT (MC4R1) and another 6 years after subsequent treatment with R-Bendamustine (MC4R2). Despite both being obtained from bone marrow biopsies, the tumor cells exhibited distinct transcriptional clusters, with four transcriptional subclusters identified (panel A). These subclusters presented similar CNV patterns but exhibited different gene expression pathway enrichment profiles (panel B-C). Cell–cell interaction analysis revealed that subclusters from the same tumor had similar cell crosstalk patterns, with both interacting with CD4.Treg cells via the CD86-CTLA4 axis, while the 2nd relapse led to additional survival support being gained from myeloid cells via BAFF signaling and Tfh cells via CXCR5-CXCL13 interactions (panel D). To infer clonal evolution, trajectory analysis was first performed and showed that the tumor cells from the 1st relapse evolved into C1 tumor cells in the 2nd relapse, which further developed into C2 cells that interacted more with T cells via CD70–CD27 interactions (panel D-E). Moreover, we were able to perform WGS on an additional DNA sample collected at diagnosis and showed that the primary clone, which carried *PRKDC* and *TBL1XR1*, was eliminated by ASCT treatment (panel F). However, some of the founder clone cells developed into relapsed tumors via the acquisition of mutations in *CD28* and *FOXO1*. Subsequently, the tumor clone accumulated the *SELENBP1* mutation, gained survival signals from myeloid cells, and differentiated into a subclone that triggered T-cell exhaustion via CD70 (panel F-G). These data suggest that in MC4, the inferred dominant clone in the primary tumor was eliminated by the treatment, but a subclone surged during the first relapse and developed in the second relapse via linear clonal evolution.

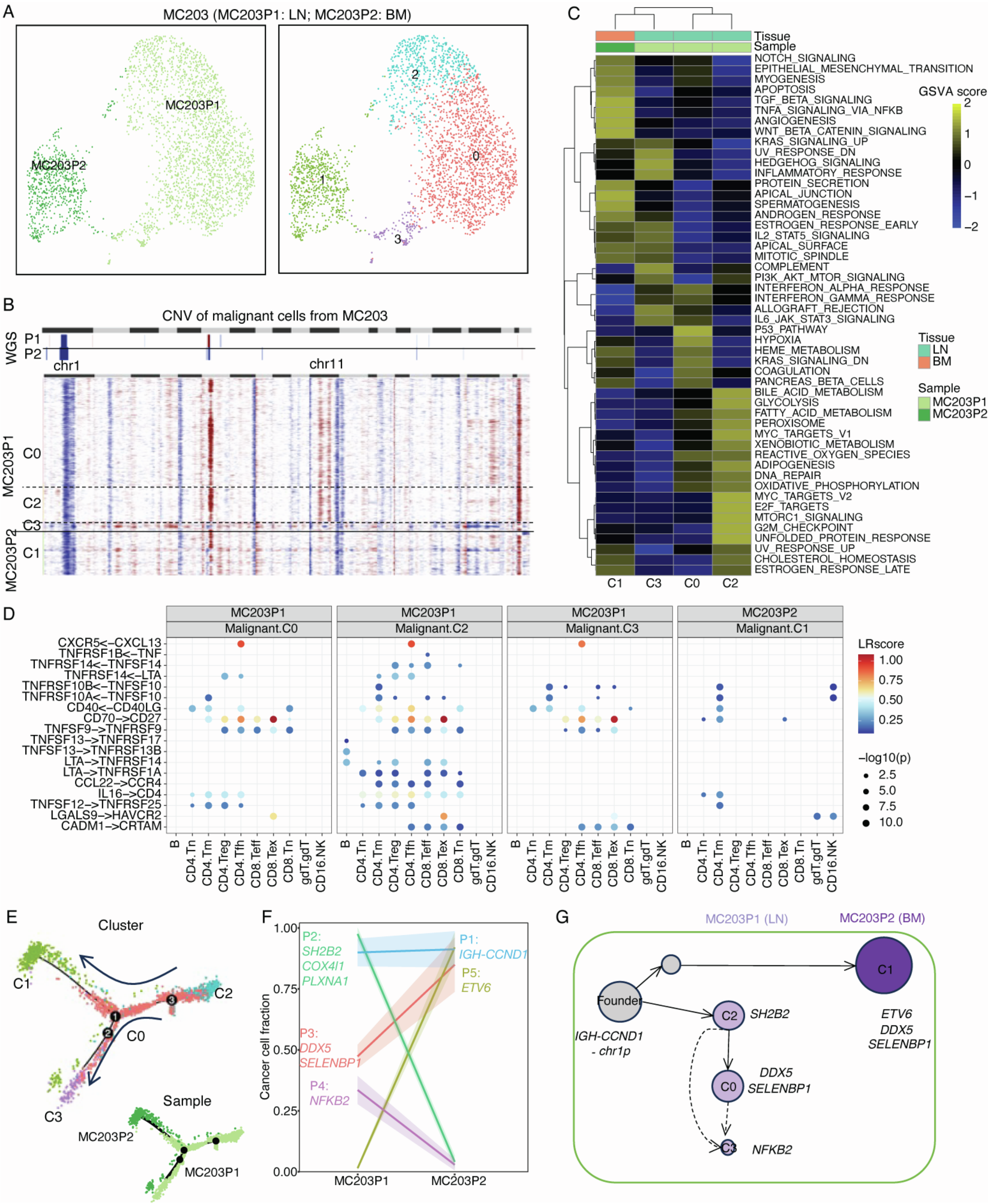

Figure S8. Tumor evolution in the untreated patient MC203. Related to Figure 5.
(A) UMAP of malignant cells identified from the tumors of patient MC203. (**B**) The inferred CNVs from WGS (top) and scRNA-seq (middle) in MC203 samples. Red indicates copy number gain, and blue indicates copy number loss. (**C**) GSVA of hallmark pathways in each transcriptional subcluster. (**D**) L–R interactions between malignant subcluster cells and TME cells. **(E)** Trajectory of malignant cells in MC203, which are colored according to subclusters. The root was inferred from the first sampling of tumor cells. (**F**) Clustering of somatic mutations by cancer cell fraction between two tumor samples was performed by PyClone-VI. The representative mutated genes are labeled. (**G**) Inferred clonal evolution in MC203. The light purple and dark purple represent the subclusters detected in the two tumor samples. The gray represents subclusters undetected by scRNA-seq.

In patient MC203, two tumors were sequentially sampled from the lymph nodes (MC203P1) and bone marrow (MC203P2) at five-year intervals. Four transcriptional subclusters were identified from the two samples, comprising three subclusters (C0, C2, and C3) that were dominant in the lymph node tumor and one subcluster (C1) in the bone marrow tumor (panel A). Subclusters C0 and C2 showed similar CNV patterns, with additional amplifications on chr11 compared with C1, and were distinct from those of subcluster C3 (panel B). These subclusters also presented distinct transcriptional activities: the P53 pathway was enriched in C0, the MYC-targeted and MTORC1 pathways were enriched in C2, the inflammatory response pathway was enriched in C3, and the NOTCH and NF-κB signaling pathways were enriched in C1 (panel C). In addition, subclusters in the MC203P1 tumors interacted with CD8.Tex and CD4.Treg cells via the CD70-CD27 signaling, and interacted with CD4.Tfh cells via CXCR5-CXCL13, while subcluster C2 cells interacted more strongly with T cells via *TNFSF13* and *LTA* interactions (panel D). Trajectory analysis revealed a path starting from C2, branching into C3 or C1, with C0 acting as an intermediate stage (panel E). However, when we further explored clonal evolution using genetic mutations, ClonEvol failed to infer the clonal architecture, indicating that the two tumors may have evolved from separate subclone populations. According to the PyClone-VI results, five clone populations were identified (panel F). All cells from the 1st lymph node tumor (MC203P1) had *IGH-CCND1* (P1) and *SHM2B2*/*COX4I1*/*PLXNA1* (P2) mutations with a CCF of 1, whereas the 2nd bone marrow tumor cells had *IGH-CCND1* (P1) plus *ETV6* (P5) and *DDX5/SELENBP1* (P3) mutations but lacked P2 mutations (panel F). We then correlated the genetic subclones with transcriptional subclusters and observed that genetic clonal evolution (P1+P2 > P3 > P4) mirrored the trajectory branch (C2 > C0 > C3) in the MC203P1 tumor (panel G). These findings suggest the presence of multiple subclones in the primary tumor, with some evolving in the 1st lymph node tumor and others acquiring additional mutations and expanding in the 2nd bone marrow tumor.

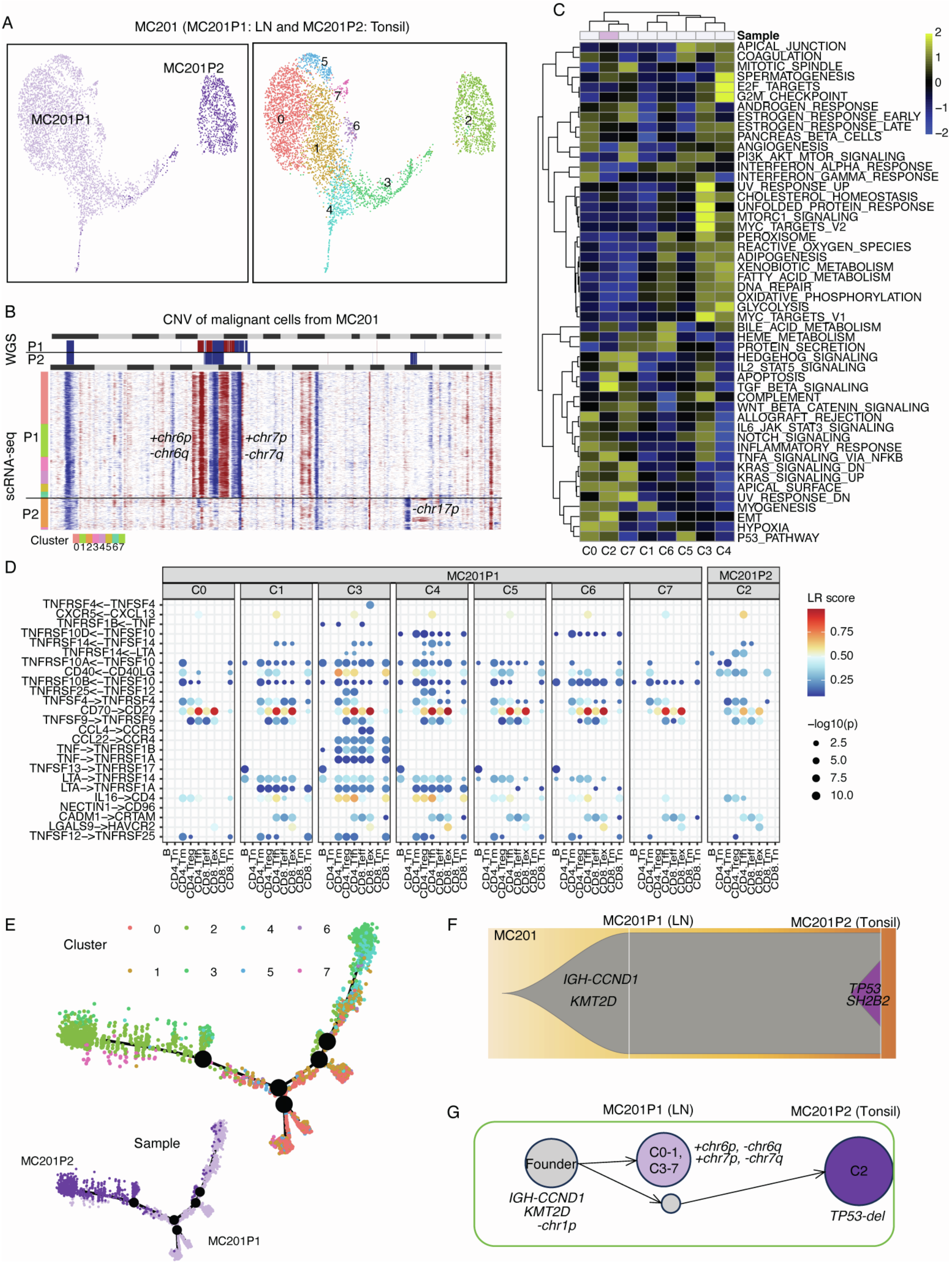

Figure S9. Tumor evolution in the untreated patient MC201. Related to Figure 5.
(**A**) UMAP of malignant cells. (**B**) The inferred CNVs from WGS (top) and scRNA-seq (middle) data. Red indicates copy number gain, and blue indicates copy number loss. (**C**) GSVA of hallmark pathways in each transcriptional subcluster. (**D**) L–R interactions between malignant subcluster cells and TME cells. (**E**) Trajectory of malignant cells, which are colored according to

subclusters and samples. (**F**) Fish plot showing patterns of tumor clonal evolution inferred based on somatic mutations from WGS data. The representative mutated genes are labeled. (**G**) Inferred clonal evolution in MC201.

MC201, without treatment, also exhibited distinct transcriptional subclusters between the two tumor samples. The 2nd tonsil tumor (MC201P2, collected 1.5 years after the 1st lymph node tumor (MC201P1)) was dominated by subcluster C2, whereas the other six subclusters were in the 1st lymph node tumor (panel A). Interestingly, there was a significant difference in the CNV patterns between the two tumors in both the bulk WGS data and the scRNA-seq data. Specifically, +chr6p, -chr6q, +chr7p, and -chr7q were detected in the 1st lymph node tumor, while -chr17q was detected in the 2nd tonsil tumor (panel B). Moreover, subclusters from different tumors also exhibited different enrichments of hallmark pathways (panel C). Further cell crosstalk analysis revealed similar cell–cell interactions among most subclusters, with strong interactions with T cells via the CD70–CD27, IL16–CD4, and CD40–CD40LG complexes (panel D). The trajectory analysis revealed multiple branches in the 1st lymph node tumor and a distinct branch in the 2nd tonsil tumor (panel E). Genetically, the tumors derived from tonsils exhibited additional *TP53* deletions (-chr17p) and *SH2B2* mutations (panel F). This finding suggested that the 2nd tonsil tumor may not have evolved from the 1st lymph node-dominant tumor clone. Instead, it likely evolved from the founding tumor cells that existed before clinical diagnosis, possibly owing to additional *TP53* deletions acquired during progression (panel G).

Table S1. Sample information, sequencing tissue and methods, and classical mutations for each sample, related to Figure 1.

Patient and sample information									Tumor tissue source*			Highgly frequent somatic alterations		
**DonorID**	**Gender**	**Health status**	**Age at Diagnosis**	**Sample**	**Type**	**Time to diagnosis (months)**	**Treatment****	**Data source**	**WGS**	**scRNAseq**	**scBCRseq**	**IGH-CCND1_SV**	**ATM_mut**	**TP53_mut/del**
MC1	male	MCL	77	**MC1P**	Diagnostic	0	/	This study	Tongue base	/	/	yes	yes	yes
				**MC1R**	Relapse	48	CHOP + RadioX	This study	Pleural fluid	Pleural fluid	Pleural fluid	yes	yes	yes
MC2	male	MCL	82	**MC2P**	Diagnostic	0	/	This study	LN	LN	LN	yes	yes	yes
				**MC2R**	Relapse	22	R-CHOP	This study	Tonsil	/	/	yes	yes	yes
MC3	male	MCL	56	**MC3P1**	Untreated	68	watchful waiting	This study	LN	BM	BM	yes	no	no
				**MC3P2**	Untreated	78	watchful waiting	This study	BM	BM	BM	yes	no	no
				**MC3Ri**	Relapse	164	ASCT	This study	Intestine	Intestine	Intestine	yes	no	no
				**MC3Rb**	Relapse	164	ASCT	This study	/	BM	BM	/	/	/
MC4	male	MCL	52	**MC4P**	Diagnostic	0	/	This study	LN	/	/	yes	yes	no
				**MC4R1**	Relapse	121	ASCT	This study	LN	BM	BM	yes	yes	no
				**MC4R2**	Relapse	179	BR	This study	BM	BM	BM	yes	yes	no
MC201	male	MCL	51	**MC201P1**	Untreated	16	watchful waiting	This study	LN	LN	/	yes	no	no
				**MC201P2**	Untreated	33	watchful waiting	This study	Tonsil	Tonsil	/	yes	no	yes
MC202	male	MCL	48	**MC202P**	Diagnostic	0	/	This study	/	LN	LN	/	/	/
				**MC202R**	Relapse	59	ASCT	This study	BM	BM	BM	yes	yes	no
MC203	male	MCL	49	**MC203P1**	Diagnostic	0	/	This study	LN	LN	LN	yes	no	no
				**MC203P2**	Untreated	68	watchful waiting	This study	BM	BM	BM	yes	no	no
MC204	female	MCL	77	**MC204P**	Diagnostic	0	/	This study	/	BM	BM	/	/	/
MC205	male	MCL	59	**MC205P**	Diagnostic	0	/	This study	/	BM	BM	/	/	/
				**MC205R**	Relapse	30	ASCT	This study	/	LN	LN	/	/	/
MC206	female	MCL	78	**MC206P**	Diagnostic	0	/	This study	BM	BM	BM	yes	yes	no

				**MC206R**	Relapse	16	R-FC	This study	BM	BM	BM	yes	yes	no
MC207	male	MCL	65	**MC207R**	Relapse	23	R-FC	This study	LN	LN	LN	yes	yes	yes
MC101	male	MCL	76	**MC101P**	Diagnostic	0	/	This study	LN	/	/	yes	yes	no
				**MC101R**	Relapse	64	R-CHOP, local irradiation	This study	LN	/	/	yes	yes	no
MC102	male	MCL	61	**MC102P**	Diagnostic	0	/	This study	na	/	/	yes	no	no
				**MC102R**	Relapse	23	ASCT	This study	na	/	/	yes	no	no
MC103	male	MCL	69	**MC103P**	Diagnostic	0	/	This study	LN	/	/	yes	no	yes
				**MC103R**	Relapse	56	ASCT	This study	Pleural fluid	/	/	yes	no	yes
MC104	male	MCL	74	**MC104P**	Diagnostic	0	/	This study	BM	/	/	yes	yes	no
MC105	male	MCL	55	**MC105R**	Relapse	7	ASCT	This study	Pleural fluid	/	/	no	no	yes
MC106	male	MCL	67	**MC106P**	Diagnostic	0	/	This study	BM	/	/	yes	no	yes
				**MC106R**	Relapse	28	ASCT	This study	BM	/	/	yes	no	yes
MC5	male	MCL	53	**MC5P**	Diagnostic	0	/	This study	LN	/	/	yes	no	no
				**MC5R1**	Relapse	48	ASCT	This study	LN	/	/	yes	no	no
MC6	female	MCL	44	**MC6R1**	Relapse	30		This study	LN	/	/	yes	yes	no
MC7	male	MCL	41	**MC7P**	Diagnostic	0	ASCT	This study	LN	/	/	yes	yes	no
MC8	male	MCL	62	**MC8R**	Relapse	66	Allotransplant	This study	BM	/	/	yes	no	no
MC301	na	MCL	na	**MC301P**	Diagnostic	0	/	This study	na	/	/	yes	no	yes
				**MC301R**	Relapse	na	na	This study	na	/	/	yes	no	yes
NC01	female	Health	na	**BM_Benrich**	Control	/	/	This study	/	BM	/	/	/	/
NC02	female	Health	66 to 70	**BM1**	Control	/	/	E-MTAB-11536	/	BM	/	/	/	/
NC03	male	Health	51 to 55	**BM2**	Control	/	/	E-MTAB-11536	/	BM	/	/	/	/
NC04	male	Health	71 to 75	**BM3**	Control	/	/	E-MTAB-11536	/	BM	/	/	/	/
NC05	male	Health	61 to 65	**BM4**	Control	/	/	E-MTAB-11536	/	BM	/	/	/	/
NC06	female	Health	56 to 60	**BM5**	Control	/	/	E-MTAB-11536	/	BM	/	/	/	/

NC07	female	Reactive hyperplasia	>60	**RLN1**	Control	/	/	GSE203610	/	LN	/	/	/	/
NC08	male	Reactive hyperplasia	>60	**RLN2**	Control	/	/	GSE203610	/	LN	/	/	/	/
NC09	female	Reactive hyperplasia	>60	**RLN3**	Control	/	/	GSE203610	/	LN	/	/	/	/

*__Sequenced tissue__: BM: bone marrow; LN: lymph node.

** **Treatment:** CHOP: cyclophosphamide, doxorubicin, vincristine, and prednisone; R-CHOP: rituximab plus CHOP; ASCT: Autologous stem cell transplants; BR: bendamustine and rituximab; R-FC: rituximab plus fludarabine and cyclophosphamide.

Table S2. Quality control of WGS samples, related to Figure 2.

Sample	Tumor/Germline type	Clean_Reads	Reads_with_adapter	Reads_with_low_quality	Reads_with_n_rate_exceed	Mapping_Rate	properly_pair_rate	Duplication_Rate	Average_depth(rmdup)	Coverage(≥1X)	Coverage(≥5X)	Coverage(≥10X)	Coverage(≥20X)
MC1P	All tumor	991394838	4317938	0	0	99,88	98,3	13,33	43,71	99,74	99,07	98,12	94,6
MC1R	All tumor	841858370	3616399	0	0	99,85	97,7	11,42	37,87	99,31	98,63	97,88	90,49
MC1PBL	Peripheral blood	626321126	514068	0	0	99,83	96,3	19,72	25,52	99,72	98,93	95,82	74,49
MC2P	All tumor	719916466	430039	0	0	99,86	97	27,4	26,57	99,65	98,84	96,31	77,04
MC2R	All tumor	767707090	3227980	0	0	99,89	97,8	12,4	34,17	99,69	99,06	97,86	88,8
MC2PBL	Peripheral blood	845213088	446265	0	0	99,83	96,3	29,57	30,17	99,74	99,22	97,82	86,05
MC3P1	All tumor	798625856	4266815	0	0	99,89	97,5	10,87	36,09	99,81	99,48	98,73	92,46
MC3P2	All tumor	990784788	915295	0	1,36	99,55	92,3	40,7	27,09	99,87	99,53	97,75	76,63
MC3Ri	All tumor	848574688	2674040	0	0	99,85	98,1	12,12	37,89	99,74	99,26	98,46	92,17
MC3PBL	Peripheral blood	951892200	3332819	0	0	99,9	97	18,46	39,34	99,85	99,56	99,05	94,49
MC4P	All tumor	815060250	2542479	0	0	99,91	98,2	17,62	34,16	99,7	99,11	98,17	90,03
MC4R1	All tumor	742737150	3982850	0	0	99,88	98,5	16,55	31,51	99,75	99,25	97,85	87,18
MC4R2	Sorted B cells	1265715314	1555199	0	1,56	99,63	92,2	44,07	32,67	99,87	99,59	98,49	85,95
MC4PBL	Peripheral blood	935768986	3546578	0	0	99,89	97,9	18,55	38,71	99,8	99,46	98,91	94,19
MC201P1	Sorted B cells	881768976	1038431	0	4,01	99,49	93,8	34,79	26,51	99,87	99,49	96,78	72,87
MC201P2	Sorted B cells	940785716	3426392	0	0,31	99,53	93,9	37,56	27,1	99,87	99,56	97,75	77,31
MC201PBL	Sorted non-B cells	939687274	2835107	0	0,37	99,5	94,2	37,67	27,01	99,87	99,55	97,96	78,59
MC202R	Enriched B cells	909680594	2576935	0	1,97	99,43	96,1	37,91	26,05	99,86	99,5	97,45	74,11
MC202PBL	Enriched non-B cells	928496680	2170380	0	2,31	99,41	94,7	37,2	26,85	99,88	99,56	97,98	78,54
MC203P1	Sorted B cells	974426914	5569223	0	0,23	99,6	94	40,73	26,62	99,85	99,49	97,61	76,1
MC203P2	Sorted B cells	842665164	1101255	0	3,26	99,23	94,9	31,05	26,7	99,84	99,48	97,72	76,91
MC203PBL	Sorted non-B cells	946052700	3326065	0	1,74	99,46	93,8	40,97	25,64	99,86	99,5	97,48	71,85
MC206P	Enriched B cells	919708034	2114878	0	2,39	99,65	95,4	37,77	26,47	99,11	98,76	97,97	76,89
MC206R	Enriched B cells	1028879362	2304553	0	2,51	99,51	95	38,74	29,05	99,15	98,83	98,35	85,99
MC206PBL	Enriched non-B cells	900717276	4646345	0	0,28	99,62	94	35,2	26,99	99,13	98,8	98,24	81,91

MC207R	Sorted B cells	912859940	3151491	0	1,69	99,54	94,1	38,07	26,07	99,87	99,38	96,44	71,81
MC207PBL	Sorted non-B cells	916999674	2092304	0	0,72	99,43	94,3	35,33	27,31	99,86	99,55	98,13	81,12
MC101P	All tumor	887659674	2618748	0	0	99,92	98,3	18,58	36,74	99,81	99,38	98,63	90,29
MC101R	All tumor	713739744	2947838	0	0	99,92	98,1	17,1	30,07	99,76	99,22	97,33	82,5
MC101PBL	Peripheral blood	721520092	2981851	0	0	99,92	97,9	17,3	30,31	99,79	99,34	98,04	86,81
MC102P	All tumor	707503964	3845772	0	0	99,91	96,5	18,33	29,27	99,76	99,3	97,92	85,23
MC102R	All tumor	819936446	4160674	0	0	99,91	97,7	17,6	34,3	99,78	99,4	98,47	90,12
MC102PBL	Peripheral blood	682646346	344587	0	0	99,68	96,7	25,47	25,79	99,69	99,06	96,62	76,74
MC103P	All tumor	704431070	2487475	0	0	99,88	97,1	18,87	28,97	99,82	99,39	97,8	83,55
MC103R	All tumor	739897038	3074849	0	0	99,92	98	15,05	31,92	99,82	99,43	98,23	87,53
MC103PBL	Peripheral blood	782857006	2900301	0	0	99,91	97,8	15,81	33,46	99,82	99,45	98,56	91,09
MC104P	All tumor	796067992	3033111	0	0	99,91	98	16,14	33,92	99,81	99,4	98,56	89,48
MC104PBL	Peripheral blood	822697066	3162232	0	0	99,85	98,1	15,16	35,44	99,8	99,41	98,61	92,28
MC105R	All tumor	829136942	3276072	0	0	99,81	98	16,21	35,27	99,49	98,7	97,55	86,86
MC105PBL	Peripheral blood	719792514	3174538	0	0	99,88	98	14,95	31,09	99,81	99,4	98,17	87,96
MC106P	All tumor	887217388	3113794	0	0	99,91	97,6	18,1	36,89	99,46	98,7	97,97	90,53
MC106R	All tumor	720257970	2755165	0	0	99,91	98,2	16,28	30,64	99,65	98,66	97,31	85,46
MC106PBL	Peripheral blood	874975534	3522182	0	0	99,9	98,4	17,9	36,5	99,8	99,46	98,82	93,23
MC5P	All tumor	959220034	2306575	0	0	99,88	96,2	22,02	37,85	99,8	99,44	98,88	93,98
MC5R1	All tumor	924411966	2405578	0	0	99,9	98,4	19,76	37,72	99,76	99,33	98,7	93,87
MC5PBL	Peripheral blood	1212969312	4506172	0	0	99,91	97,5	21,1	48,57	99,83	99,49	98,95	96,65
MC6R1	All tumor	785024260	2492637	0	0	99,92	98,3	17,7	32,89	99,14	98,49	97,85	92,42
MC6PBL	Peripheral blood	974327506	4020901	0	0	99,92	97,7	20,27	39,47	99,26	98,81	98,46	96,79
MC7P	All tumor	859010296	3693112	0	0	99,89	96,4	19,98	34,81	99,79	99	97,93	89,64
MC7PBL	Peripheral blood	949602548	3544320	0	0	99,89	97,3	22,39	37,38	99,82	99,5	98,9	93,47
MC8R	All tumor	957128152	3887027	0	0	99,92	97,8	18,21	39,77	99,2	98,51	97,96	91,89
MC8PBL	Peripheral blood	846705230	2984525	0	0	99,92	96,9	18,57	34,94	99,81	99,46	98,67	91,6
MC301R	All tumor	812654872	2441239	0	0	99,92	97,2	19,67	33,12	99,79	99,02	97,85	87,19
MC301P	All tumor	736877316	3532554	0	0	99,91	97,2	18,52	30,46	99,51	98,59	96,95	81,17
MC301PBL	Peripheral blood	740857322	4200952	0	0	99,93	97,2	16,61	31,32	99,8	99,4	98,21	87,78
